# Results from ten years of post-market environmental monitoring of genetically modified MON 810 maize in the European Union

**DOI:** 10.1371/journal.pone.0217272

**Published:** 2020-04-24

**Authors:** Lieselot Bertho, Kerstin Schmidt, Jörg Schmidtke, Ivo Brants, Rocío Fernández Cantón, Conchi Novillo, Graham Head

**Affiliations:** 1 Monsanto Company, Saint Louis, Missouri, United States of America; 2 BioMath, Rostock-Warnemünde, Germany; 3 Monsanto Company, Brussels, Belgium; 4 Monsanto Company, Madrid, Spain; University of Tennessee, UNITED STATES

## Abstract

In European regulations for the deliberate release into the environment of genetically modified organisms (GMO), the objective of General Surveillance in Post-Market Environmental Monitoring is defined as the identification of the occurrence of adverse effects of the GMO or its use which were not anticipated in the environmental risk assessment (ERA). Accompanying the commercial cultivation in the EU of maize event MON 810, General Surveillance was implemented by Monsanto, the authorization holder, on a voluntary basis. We carried out a statistical analysis on the pooled results of ten years of farmer questionnaires, which were a part of this General Surveillance, amounting to 2,627 farmer fields in eight European countries in the period 2006–2015. This analysis did not reveal any unexpected adverse effects associated with the cultivation of MON 810. Results from farmer questionnaires confirmed that the cultivation of MON 810 resulted in a significant reduction in the use of pesticides, efficient protection against the target pests, and healthier, higher yielding crops compared to conventional maize. MON 810 also had reduced susceptibility to disease and pests when compared to conventional maize. Monitoring characteristics related to environment and wildlife revealed no significant differences between MON 810 and conventional maize. Literature searches, that were also conducted as part of General Surveillance, identified a comprehensive set of publications addressing environmental safety as well as food and feed safety aspects of MON 810. None of the publications indicated any adverse effect of MON 810 that was not anticipated in the initial ERA, nor did they lead to a change in the conclusions of the initial risk assessment that demonstrated the safety of MON 810. The development of resistance by the target pests (*Ostrinia nubilalis*, ECB and *Sesamia nonagrioides*, MCB) was the only potential adverse effect identified in the ERA of MON 810 cultivation in the EU. The extensive safety data package for MON 810, the robust weight of evidence demonstrating both its safety and benefits, and the history of safe use of MON 810 for 15 years in the EU, indicates that focussing the General Surveillance of MON 810 on literature searches and farmer complaint systems would be appropriately protective. This will allow the identification of potential adverse effect not anticipated in the initial ERA without the intensive effort and organizational challenges of farmer questionnaires.

## Introduction

The European corn borer (*Ostrinia nubilalis*, ECB) and the Mediterranean corn borer (*Sesamia nonagrioides*, MCB) severely damage European maize production by foliage feeding and stalk tunneling in the field, and via secondary effects such as increased mycotoxin levels [1]. Conventional practices—not involving genetically modified (GM) hybrids—for controlling these pests include the use of synthetic insecticides in Integrated Pest Management (IPM) programs but these have variable efficacy.

In the 1990’s, Monsanto developed the GM maize event MON 810, expressing the naturally occurring *Bacillus thuringiensis* (*Bt*) protein Cry1Ab, which provides protection against certain lepidopteran insect pests, including both ECB and MCB. In 1998, an authorization for commercial cultivation of MON 810 in the EU was obtained for a period of 10 years [2]. In 2007, the renewal application for MON 810 cultivation was submitted and now, more than a decade later, it is still pending a decision at the European Commission.

European Directive 2001/18/EC [3] introduced the obligation to implement a monitoring plan to trace and identify any direct or indirect, immediate, delayed or unforeseen effects on human or animal health or the environment of GM organisms or products after they have been placed on the market. The objectives of a Post-Market Environmental Monitoring (PMEM) plan were defined as 1) confirming that assumptions in the environmental risk assessment (ERA) regarding the occurrence and impact of potential adverse effects of the GMO or its use are correct (“Case Specific” or CS), and 2) identifying any adverse effects of the GMO or its use on human or animal health or the environment which were not anticipated in the ERA (“General Surveillance” or GS). Since then, additional guidelines on PMEM and GS have been published [4–6].

The initial authorization for MON 810 cultivation in the EU was granted before Directive 2001/18/EC entered into force and included the requirement to conduct CS monitoring [2]. The development of resistance to the expressed Cry1Ab protein in the target pests was the only potential adverse effect identified in the ERA of MON 810 cultivation in the EU. While it has been debated whether development of resistance in insects is an agronomic/economic effect rather than an environmental one, CS monitoring focused on detecting resistant insect populations and on the implementation of, and compliance with, the Insect Resistance Management (IRM) plan. The results of the CS monitoring show no indication of reduced Cry1Ab susceptibility after 15 years of commercial planting for the two target pests in the EU, indicating that the established IRM plan for MON 810 has been a conservative, pro-active and effective tool in sustaining the efficacy of the product [8, 25–34]. It should be noted that several product characteristics and aspects of pest biology contribute to a low likelihood of changes in susceptibility of the target pests against the MON 810 Cry1Ab protein in the EU (e.g. use of a high-dose product and the mobility of ECB and MCB populations). Furthermore, resistance is more likely to develop when there is high adoption of the trait and, even though there are certain areas where the MON 810 adoption rate is high, in general it is cultivated on a relatively small-scale in the EU [7, 8].

The initial MON 810 authorization did not include a requirement to conduct GS. Nevertheless, the authorization holder, Monsanto, has implemented GS since 2006 on a voluntary basis following an approach developed by industry that combines farmer questionnaires, searches of relevant scientific publications, company stewardship programs, and surveillance networks where appropriate [9]. Questionnaires directed at farmers growing GM crops are considered one of the possible approaches for collecting first hand data on the performance and impacts of GM crops in comparison with conventional crop cultivation [10–14]. At the same time, searches of peer-reviewed literature can support and provide new context to the original ERA by identifying new publications related to MON 810 conducted worldwide including experimental research, developmental and advisory studies on crop cultivation, hybrid registration and hybrid performance trials.

In this paper, the results from ten years of farmer questionnaires and literature searches that were independently conducted as part of PMEM for commercial cultivation of MON 810 in the EU are described. A comprehensive statistical analysis of the pooled data from the ten years of farmer questionnaires was conducted, including an analysis of trends within the data. With MON 810 being the only GM crop cultivated commercially in the EU for nearly 15 years, this summary of GS information illustrates the experience gained through MON 810 cultivation and provides an opportunity to reflect on possible adjustments to the current PMEM approach. As far as we know, this is also the longest duration and most comprehensive PMEM data set ever developed for a GM crop.

## Materials and methods

### Farmer questionnaires

#### Design of the questionnaire

The methodology for using farmer questionnaires in PMEM of GMO has been published [11–24]. For the MON 810 farmer questionnaires, three subjects were identified which might be influenced by and observed during cultivation, *i*.*e*. agronomic practices, plant characteristics in the field, and effects on environment and wildlife (Table 1). For each subject, practical endpoints (monitoring characteristics) suitable to detect emerging effects from the cultivation of MON 810 were defined.

**Table 1:** Subjects, characteristics and response categories for MON 810 farmer questionnaires.

Subject	Monitoring characteristics–observations of MON 810	*Categories*		
		*as usual*	*different*: *plus* or *changed*	*different*:*minus*
Agronomic practices	Crop Rotation	as usual	changed	-
	Time of planting	as usual	later	earlier
	Tillage and planting technique	as usual	changed	-
	Insect control practices	as usual	changed	-
	Fertilizer application	as usual	changed	-
	Time of harvest	as usual	later	earlier
	Weed control practices	as usual	changed	-
	Fungal control practices	as usual	changed	-
	Irrigation practices	as usual	changed	-
	Maize Borer control practice	as usual	changed	-
Characteristics in the field	Germination vigor	as usual	more	less
	Time to emergence	as usual	delayed	accelerated
	Time to male flowering	as usual	delayed	accelerated
	Plant growth and development	as usual	delayed	accelerated
	Incidence of stalk/root lodging	as usual	more	less
	Time to maturity	as usual	delayed	accelerated
	Yield	as usual	higher	lower
	Occurrence of MON 810 volunteers	as usual	more	less
Environment and wildlife	Disease susceptibility	as usual	more	less
	Pest susceptibility	as usual	more	less
	Weed pressure	as usual	more	less
	Performance of fed animals	as usual	changed	-
	Occurrence of insects	as usual	more	less
	Occurrence of birds	as usual	more	less
	Occurrence of mammals	as usual	more	less

For each monitoring characteristic, qualitative categories were defined to direct the assessment of the cultivation of MON 810 compared to conventional maize. For most characteristics, the possible categories of answers were “as usual” and “different” with the latter category subdivided into “plus” (e.g. later, delayed, higher, more) or “minus” (e.g. earlier, accelerated, lower or less). For some characteristics related to agronomic practices and performance of fed animals, a subdivision of the “different” category based on quantitative terms was not meaningful.

A farmer questionnaire was designed to obtain data on the monitoring characteristics. Because in an agricultural landscape other factors (like soil characteristics, cultivation methods or environmental factors) may influence the monitored characteristics, these were also taken into account in the questionnaire.

The questionnaire was adjusted yearly based on the experience gained in the previous season, on improvements of the statistical relevance of the collected data, and according to feedback received from the ESFA GMO Panel and the European Commission (DG Environment). The survey questions used for each year of the survey are provided in the published, annual PMEM reports [25–34].

#### Sampling

An annual survey was performed for ten years. The total population consisted of all fields within the EU where MON 810 was cultivated within the ten-year monitoring period. The sample size for the survey was determined in relation to the statistical procedure (one-sided exact binomial test, comparison of a probability with a constant), the performance requirements (type-1 error rate α = 0.01, type-2 error rate β = 0.01, constant = 0.1 (10% “different” answers)) and minimum difference of practical interest (0.03) [14]. This resulted in a minimum sample size of 2,436 fields. To obtain this minimum sample size (e.g. allowing for the exclusion from the survey of low quality questionnaires), an accumulated multiyear sample size of at least 2,500 fields was set (more details are given in S1 File).

The total number of monitoring objects, i.e. the 2,500 fields, was equally subdivided into 250 per year (first stratum). MON 810 cultivation areas in various European countries varied from year to year, meaning that the population was distributed irregularly. To handle the variation across regions and population of MON 810 cultivating farmers from year to year, a quota was applied considering:

- the countries of MON 810 cultivation in the respective year,
- the magnitude of MON 810 cultivation (ha planted per country/ ha planted in the EU), and
- local situation (average field size in the country).

The fluctuating deployment of MON 810 was taken into account by assigning the 250 fields per year to the respective countries (second stratum). Cultivation areas with high uptake of MON 810 are represented in the sample by a large number of monitored fields, whereas countries with proportionally very low cultivation were excluded from the monitoring.

Within each stratum (per year and country), the determined number of monitoring units needed to be selected. Where publicly available, the “GMO cultivation register” was used to identify regions of cultivation, the county-specific sample size was distributed in proportion to the regional cultivation and MON 810 cultivating farmers were identified by the interviewers per region. Where information in the GMO cultivation register could not be used to identify the farmers (e.g. due to privacy provisions), the interviewers had to contact farmers identified via their own research or with support of the MON 810 seed selling companies. The identity of the contributing farmers was kept confidential.

The stratified approach for sampling was intended to ensure that the monitoring area was proportional to and representative of the total regional area under MON 810 cultivation.

#### Collection of information

Information was collected by trained interviewers. The annual training addressed the background of the questions and experience gained during previous years’ surveys (uncertainties, misinterpretation of questions). To ensure consistent high data quality and to assist the interviewers in filling out the questionnaires with farmers, a 'user's manual' was developed.

#### Statistical analysis

If there is no effect of MON 810 cultivation or other influencing factors, and the question is well formulated and unambiguous, farmers will assess the situation to be “as usual”. Uncertainty or environmental impacts may result in low frequencies of differing answers. These are expected to be balanced in both “minus” and “plus” directions. If the cultivation of MON 810 or any other influencing factor has a real impact, this would result in a greater proportion of “different” answers. To test for an effect, the proportions of “different “answers were compared with the threshold of 10% by a statistical test (one-sided, comparison of a probability with a constant). Because the “as usual” and “different” answers complement each other, a closed test procedure was applied: first the “as usual” proportion was compared with the threshold of 90%. If the “as usual” proportion exceeded this threshold, this was considered to indicate no effect. Otherwise, both “plus” and “minus” proportions were compared with the 10% threshold and an effect was indicated if the threshold was exceeded by one of the proportions (more details are given in S1 File).

The data from the farmer questionnaires were analysed annually, and annual reports of the results were published [25–34]. Data from the first ten years of surveys have been used to conduct a comprehensive analysis of the pooled data including an analysis of trends within the data. In this analysis of the pooled data (ten years of monitoring, all countries), simple proportions p^s^ as well as weighted proportions p^w^ of the “as usual”, “plus” and “minus” categories of the monitoring characteristics were estimated. The model proportions p^mo^ (inclusive 99% confidence intervals) of all categories were estimated by applying a linear mixed model (more details are given in S1 File). The estimated 99% confidence intervals for the model proportions p^mo^ were compared with the defined thresholds according to the closed test procedure described above.

For each monitoring characteristic, the year-specific model proportions of all categories were estimated and assessed for any linear trends over years by fitting linear regression models and subsequently testing the estimated slopes against zero (more details are given in S1 File).

### Literature search

Since the start of the PMEM efforts, literature searches have been performed annually with the objective to detect any adverse effects of MON 810 or its use that were not anticipated in the ERA. The methodology for these literature searches was developed in-house in 2006 and further refined over the years based on experience. Considering the objective of the literature searches was not to address a specific question, they do not reflect the characteristics of a systematic literature review. Single or combinations of queries with free text terms as keywords and combinations with Booleans were used (S6 File). The keywords and keyword combinations were used to initiate searches, or collect updates, in the Web of Science database (WoS) and, since 2015, in the Centre for Agriculture and Biosciences International database (CABI). Over the years, the keywords used for the literature searches were adjusted taking into consideration newly established knowledge and experience. The keywords and keyword combinations that were used for each year of monitoring are available in the published annual PMEM reports [25–34] and are also provided in S6 File. An automated alert system was set up retrieving potentially relevant publications on a monthly basis from the Web of Science Core Collection database. Taking into account recommendations of the European Food Safety Authority (EFSA) GMO Panel [35] the CABI CAB Abstracts and Global Health database was included in the automated search starting in 2015.

All the retrieved publications were assessed for their potential relevance to the risk assessment of MON 810 based on the following inclusion/exclusion criteria: i) the objective(s) of the studies, i.e. assessment of potential effects on human and animal health or the environment of MON 810 and ii) the scope of the application, i.e. authorization for import, processing and all uses as any other maize, including the cultivation of MON 810 in the EU, and iii) the categories of information for risk assessment data requirements outlined in relevant guidelines and legislation. The list of different categories included food and feed safety (toxicity / animal feeding; molecular characterization; protein expression; toxicity *in vitro;* crop composition/nutrition; protein fate / DNA in the digestive tract; allergenicity; mycotoxins; others) or environmental safety (spillage and consequences thereof; agronomy; non target organisms (NTOs); pollen mediated gene flow; protein/DNA fate in soil; toxin fate in soil; insect resistance management (IRM); ecology; others). Because of this diversity of categories, studies with different experimental design, including review articles, were considered for inclusion in the annual PMEM reports.

A stepwise approach was followed by three different reviewers: two external reviewers with the company ToxMinds BVBA and one internal reviewer. This approach was conducted to assess the relevance of the retrieved publications to the risk assessment of MON 810. First, a rapid assessment was conducted based on the information in the title and abstract of all the retrieved publications following the inclusion/exclusion criteria described previously. In a second step, a more detailed assessment of the publications that were selected as potentially relevant within the rapid assessment was conducted. The full text of these selected, potentially relevant studies was further assessed based on the inclusion/exclusion criteria, leading to a final list of relevant publications included in the PMEM reports (provided in S5 File). In cases when the reviewers’ assessment of a retrieved study did not lead to a unanimous recommendation for relevance, the study was *de facto* included.

Using the information reported in the publications that were included in the PMEM reports (S5 File), the reviewers carried out an assessment to define if the results of the study impacted the initial risk assessment conclusions for MON 810. In accordance with the framework provided by the European Commission [36], all the included publications were assessed by summarizing the study results and conclusions, and by considering if there were any adverse effects of MON 810 reported in the studies related to human, animal or environmental safety. These assessments are available in the published annual PMEM reports [25–34] and determined whether the conclusions of the initial risk assessment that demonstrated the safety of MON 810 based on the comprehensive weight of evidence were still valid. The studies that suggested no adverse effects were considered as confirmatory studies to the initial risk assessment and no further action was taken. The studies that indicated potential adverse effects of MON 810 to human and animal health or the environment, relevant to the above-mentioned risk assessment categories, were subjected to further analysis by internal and external experts with a solid experience in the risk assessment of GM plants and by experts with technical experience in the specific area of the selected publication. This further assessment determined whether the methodological quality of the experiment described in the study adhered to the basic principles for conducting a scientific experiment (e.g. inclusion of the necessary control groups, characterization of test materials used, consideration of natural variability in study results, etc.). The results of the methodological quality assessment conducted on the studies indicating a potential adverse effect of MON 810 on human and animal health or the environment are provided in the published annual PMEM reports [25–34].

## Results

### Farmer questionnaires

#### MON 810 cultivation in the EU

Since the introduction of MON 810 in the EU, the area of commercial cultivation has quickly reached a stable footprint focused on regions in Spain and Portugal where the target pests are abundant (Table 2).

**Table 2:** Cultivation areas (ha) of MON 810 per country within the ten-year monitoring period (source: Annual Reports of the growing seasons 2006–2015) [25–34].

	2006	2007	2008	2009	2010	2011	2012	2013	2014	2015	total
Czech Republic	1,290	5,000	8,380	6,480	4,675	5,090	3,052	2,560	1,754	997	39,278
France	5,200	21,174									26,374
Germany	948	2,685	3,173								6,806
Poland		320	3,000	3,000	3,000	3,000					12,320
Portugal	1,255	4,500	4,851	5,094	4,869	7,723	9,278	8,171	8,542	8,017	62,300
Romania		350	7,146	3,344	823	588	217	835	771	2	14,074
Slovakia	30	900	1,900	794	1,249	761	189	100	411	104	6,438
Spain	53,667	75,148	79,269	76,057	76,575	97,346	116,306	136,962	131,537	107,749	950,616
**Total**	**62,390**	**110,077**	**107,719**	**94,850**	**91,191**	**114,508**	**129,042**	**148,628**	**143,015**	**116,867**	**1,118,287**

#### Sample and survey response rate

Over ten years of MON 810 PMEM in Europe, 2,627 farmer questionnaires were completed by 1,262 farmers in eight countries. The final totals (number of farmers per country and year included in the biometrical analysis) are described in Table 3.

**Table 3:** Number of completed farmer questionnaires per year and country (grey cells indicate that MON 810 was not cultivated in the corresponding country and year, see also Table 2).

	2006	2007	2008	2009	2010	2011	2012	2013	2014	2015	total
Czech Republic	38	45	51	49	39	29	22	18	0	0	291
France	58	79									137
Germany	37	37	44								118
Poland		3	10	3	10	10					36
Portugal	16	12	40	42	43	42	41	46	48	49	379
Romania		5	43	40	25	15	10	2	0	0	140
Slovakia	3	10	9	6	4	3	1	0	0	0	36
Spain	100	100	100	100	150	150	175	190	213	212	1,490
**Total**	**252**	**291**	**297**	**240**	**271**	**249**	**249**	**256**	**261**	**261**	**2,627**

The sampling procedure for the survey was impacted and complicated by several challenges:

- data on the total population of interest, *i*.*e*. the total number of fields (and the field sizes), were not known at the beginning of the monitoring project;
- the development of areas of MON 810 cultivation could not be predicted;
- country-specific promotions and bans of MON 810 led to a very irregular distribution across the strata of the sampling frame.

Since 2014 the programme has focused on Spain and Portugal in view of the area of MON 810 commercially grown.

The response rate was calculated as the number of farmers who completed the farmer survey with sufficient data quality to be included in the analysis, divided by the total number of farmers contacted. All farmer refusals were recorded, however, overall only 1% of farmers refused to participate. This unusually low refusal rate is likely due to how the surveys were presented and conducted. In advance of the review, the farmers were personally contacted and asked for their ability and willingness to participate. The 1% of farmers who refused to participate mostly did so for lack of available time to complete the survey. Less than 0.1% of farmers’ responses had to be excluded from the analysis for insufficient data quality reasons [25–34]. Therefore, the response rate was approximately 98%.

#### Longitudinal aspects

For representativeness, taking into consideration variable cultivation areas and farmers' willingness to participate in the interview, new farmers were recruited each year. Consequently, each year the survey covered a mixture of newly identified and known farmers.

Table 4 indicates the number of times that an individual farmer was surveyed across the ten years. Fifty-two percent of the farmers were surveyed across multiple years, though not necessarily for the same field. Eighty-eight percent participated up to four times and three farmers were interviewed in all ten years.

**Table 4:** Frequencies of farmers' survey participation.

Frequency of participation	Year										Farmers	
	2006	2007	2008	2009	2010	2011	2012	2013	2014	2015	Total	%
1	252	200	158	93	126	72	111	104	75	71	**1,262**	48.0
2		91	75	71	48	75	22	48	50	43	**523**	19.9
3			64	41	50	29	45	18	50	35	**332**	12.6
4				35	28	34	14	28	22	39	**200**	7.6
5					19	26	24	10	20	15	**114**	4.3
6						13	23	23	10	19	**88**	3.3
7							10	18	18	9	**55**	2.1
8								7	13	16	**36**	1.4
9									3	11	**14**	0.5
10										3	**3**	0.1
**Number of farmers**	**252**	**291**	**297**	**240**	**271**	**249**	**249**	**256**	**261**	**261**	**2,627**	100.0

#### Ten-year analysis

Data on 25 monitoring characteristics covering agronomic practices, characteristics in the field, and environmental and wildlife aspects were collected (see Table 1 and S7 File for the results). The Supporting Information lists all simple proportions p (Tables A-C in S2 File), the descriptive weighted proportions p^w^ (Tables D-F in S2 File); the results of ten-year analysis, model proportions p^mo^ (Tables G-I in S2 File); and the slopes and confidence intervals of linear regression (Table J in S2 File) per monitoring characteristic. The results of the descriptive weighted proportions p^w^ of each monitoring characteristic are also provided as figures in S3 File.

The model proportions p^mo^ estimate the mean annual monitoring character values considering multiple participation of farmers. The estimated variances of the random effect 'multiple participation of farmers' were either zero or negligible, so an influence on the monitoring characteristics could not be identified. Consequently, multiple growing seasons may be considered quasi-independent as each year represents a different growing season with different climate and growing conditions. Moreover, across growing seasons, different fields were planted with MON 810 by the same farmer due to decisions on crop rotation measures.

**Fig 1:**
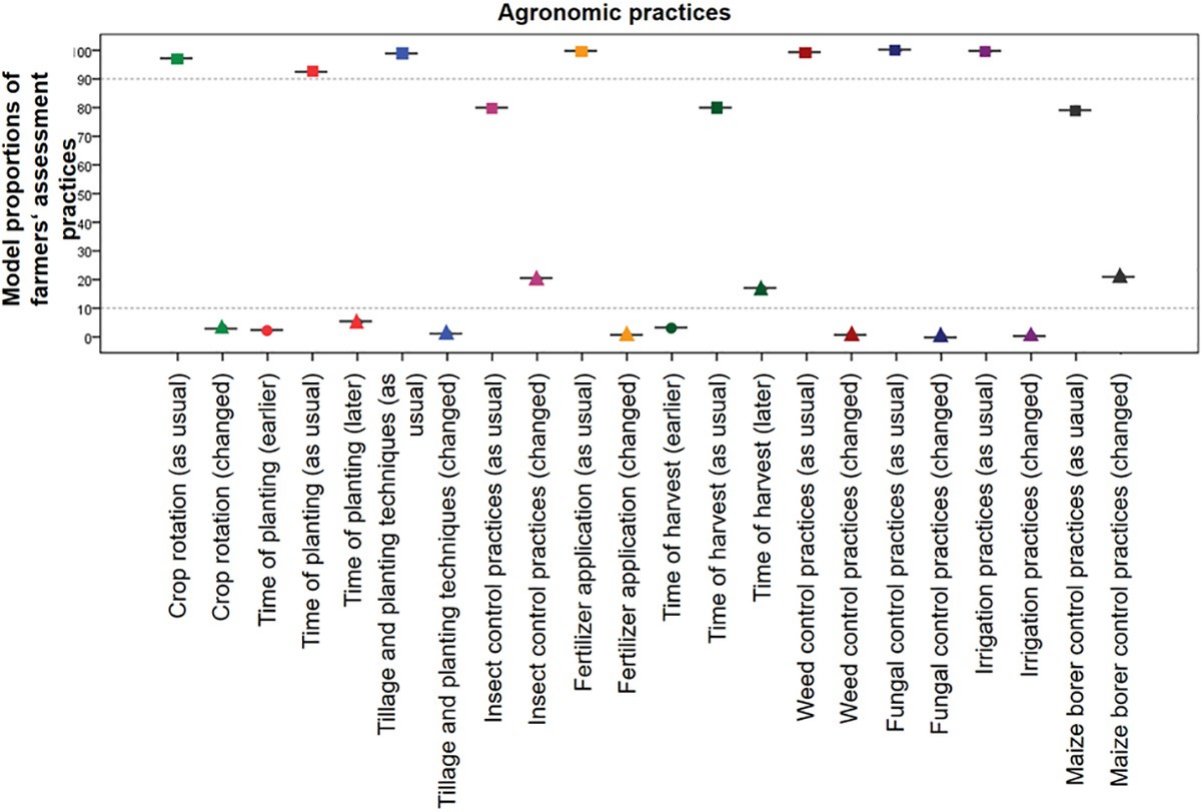
99% confidence intervals of model proportions of “as usual”, “minus” and “plus” categories for agronomic practices.

The results of the analysis of characteristics relating to agronomic practices are presented in Fig 1.

In significantly more than 10% of surveyed MON 810 fields, insect control (20.04%; CI 20.03% - 20.06%) and maize borer control practices (20.79%; CI 20.77% - 20.81%) were changed (decreased) relative to conventional maize fields. MON 810 maize was harvested significantly later than conventional comparators at 16.70% (CI: 16.69% - 16.71%) of all monitored fields. All other monitored agronomic practice characteristics (crop rotation, time of planting, tillage and planting techniques, fertilizer application, weed and fungal control practices as well as irrigation practices) of MON 810 fields were not significantly different from conventional maize fields.

**Fig 2:**
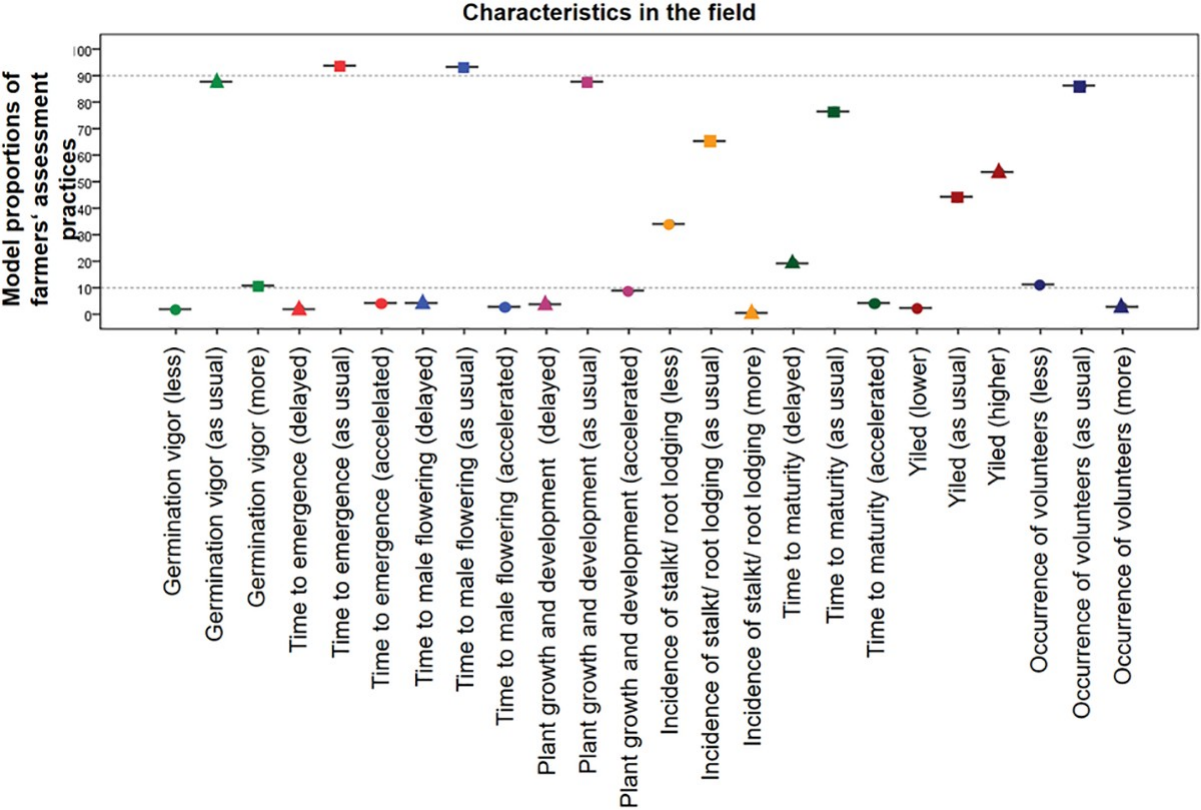
99% confidence intervals of model proportions of “as usual”, “minus” and “plus” categories for characteristics in the field.

The results of the analysis of characteristics in the field are presented in Fig 2.

Six of the characteristics in the MON 810 fields that were monitored were significantly different from what was observed in conventional maize fields (Fig 2). As expected based on the insect protection provided by MON 810, yield (53.74%; CI 53.71% - 53.76%) was significantly increased, while incidence of stalk/ root lodging (34.03%; CI 34.01% - 34.05%) and occurrence of volunteers (11.14%; CI: 11.12% - 11.16%) were significantly reduced in MON 810 fields compared to conventional maize fields. In addition, germination vigor was significantly increased (10.83%; CI: 10.82% - 10.85%) in MON 810 fields, while time to maturity was delayed (19.30%; CI: 19.28% - 19.32%). Plant growth and development was significantly changed (as usual 87.76%; CI 87.75% - 87.77%): 3.68% delayed (CI: 3.67% - 3.69%, not significant), 8.56% accelerated (CI: 8.55–8.57, not significant).

Other characteristics, such as time to emergence and time to male flowering, were not significantly different in MON 810 fields compared to conventional maize fields.

**Fig 3:**
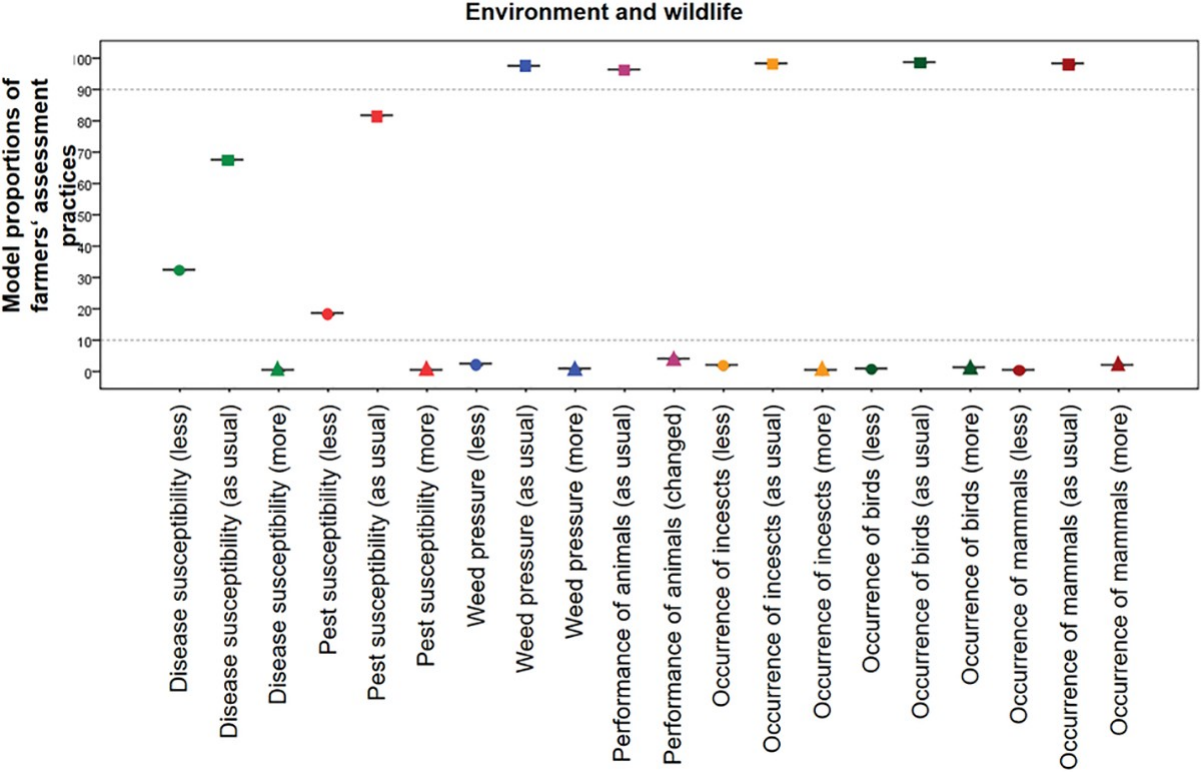
99% confidence intervals of model proportions of “as usual”, “minus” and “plus” categories for environment and wildlife.

The results of the analysis of characteristics relating to environment and wildlife are presented in Fig 3.

Two of the monitored characteristics showed a statistically significant difference between growing MON 810 and conventional maize: disease susceptibility (32.25%; CI: 32.24% - 32.27%) and pest susceptibility (18.20%; CI: 18.19% - 18.21%) were both decreased in the surveyed MON 810 fields relative to conventional maize fields. No changes were observed for weed pressure or occurrence of non-target insects, birds and mammals. Also, no difference was reported in the performance of animals fed with MON 810 compared to animals fed conventional maize (Fig 3).

Table 5 provides an overview of the results described above from the analysis for all the monitoring characteristics.

**Table 5:** Summary of the results of the ten-year analysis for all the monitoring characteristics.

Subject	Monitoring characteristic	Observations for MON 810 vs control
Agronomic practices	Crop rotation	No change
	Time of planting	No change
	Tillage and planting technique	No change
	Insect control practices	20.03% decrease
	Fertilizer application	No change
	Time of harvest	16.7% decrease
	Weed control practices	No change
	Fungal control practices	No change
	Irrigation practices	No change
	Maize borer control practice	20.79% decrease
Characteristics in the field	Germination vigor	10.83% increase
	Time to emergence	No change
	Time to male flowering	No change
	Plant growth and development	8.56% increase
	Incidence of stalk/root lodging	34.03% decrease
	Time to maturity	19.3% decrease
	Yield	53.74% increase
	Occurrence of MON 810 volunteers	11.14% decrease
Environment and wildlife	Disease susceptibility	32.25% decrease
	Pest susceptibility	18.20% decrease
	Weed pressure	No change
	Performance of fed animals	No change
	Occurrence of insects	No change
	Occurrence of birds	No change
	Occurrence of mammals	No change

#### Trend analysis

Table J in S2 File presents the results of the trend analysis over the ten years. A trend analysis can indicate if the findings remain stable over time. Although some parameters changed across years as described below, overall the changes were minor and did not modify the initial findings.

The ratio of fields reported to have changed crop rotation grew by 0.96% per year, indicating that MON 810 was cultivated in slightly more fields with different previous crops compared to conventional maize. There was also a minor increase in the proportion of fields with changed tillage and planting techniques (0.37% per year) as well as with later time of planting (0.39% per year). In contrast, the difference in fertilizer application and harvest time of MON 810 compared with conventional maize both diminished over years (resp. 0.11% and 0.18% per year). No trends were detectable for insect, weed and fungal control practices, irrigation practices and maize borer control practices.

Time to male flowering and yield showed no statistically significant trends over time, while the other observed characteristics in the field showed significant (though minor) trends. Most trends in field characteristics indicated an increase in the proportion of “as usual” responses.

For environment and wildlife monitoring characteristics no trends were observed for weed pressure, occurrence of birds and the performance of animals fed on MON 810. However, the proportion of surveyed MON 810 fields for which disease susceptibility and occurrence of non-target insects and mammals was assessed to be “as usual” increased per year.

### Literature search

The use of the Web of Science Core Collection database and the CABI CAB Abstracts and Global Health database ensured a broad coverage as well as a high probability of retrieving relevant publications from several peer-reviewed and high impact journals. Over the ten years of PMEM, 375 publications and 48 review papers related to MON 810 and Cry1Ab published in peer-reviewed journals were identified (See S4 File). Among the retrieved publications, 102 were related to food and feed safety while 273 of the publications were related to environmental safety. An assessment of each of the relevant publications was conducted in the context of the yearly PMEM report. A full list of the retrieved relevant publications is provided in S5 File. None of them led to a change in the conclusions of the initial risk assessment that demonstrated the safety of MON 810.

## Discussion

Farmer questionnaires use farmers' experience with a product and provide first-hand knowledge of agricultural environments and crop performance. With the challenges associated with the evolution of the cultivated area, a stratified sampling approach had to be used to ensure that the monitoring area was proportional to and representative of the total regional area cultivated with MON 810 in the EU. By doing so, it was confirmed that the cultivation of MON 810 in the EU resulted in a significantly reduced proportion of fields on which standard insect control practices, in particular maize borer control, were applied. Standard pest control practices require the use of synthetic insecticides. Due to the protection provided by the Cry1Ab protein expressed in MON 810, a decrease in these and other maize borer control practices is expected. The same effect has been observed in other regions of the world that have adopted insect-protected GM crops. Overall, a decrease of as much as 56.1% in insecticide active ingredient use globally from using GM insect-protected maize was reported based on data gathered from 1996 to 2016 [37–39]. Hutchinson et al. [40] showed that, as a result of area-wide pest suppression, economic benefits accrue not only to farmers planting Bt maize but also to those planting non-Bt maize, and that these suppression benefits can equal or exceed the benefits to Bt maize growers [41].

Adoption of MON 810 technology reduces the use of pesticides, provides efficient protection against the target pests and results in a healthier and better yielding crop. The significantly improved control of the targeted corn borers leads to improved plant growth and development in general, delayed time to maturity and consequently a delayed time to harvest for MON 810 compared to conventional maize. The results of the farmer questionnaire analysis confirmed a significantly later harvesting time of MON 810 maize plants compared to conventional maize. An indicator for farmers to determine time of maize harvest is the grain moisture. It has been demonstrated previously that Bt maize plants tend to have higher grain moisture compared to non-Bt maize [42], potentially caused by accelerated senescence of maize plants when infested with corn borers [43]. Therefore, the delayed time to maturity and later harvesting time that was observed for MON 810 is likely due to preserved grain moisture in the absence of corn borer infestations. Harvesting early also prevents further infestation and damage by the corn borer larvae and consequently reduces the amount of lodged plants or dropped ears [44]. Later harvesting also results in yield protection when farmers plant MON 810 instead of conventional maize. The availability of high quality germplasm used by MON 810 cultivating farmers likely adds to the observed increase in yield. This result is in line with studies conducted with MON 810 or Bt maize in different non-EU countries and regions that are subject to high pressure of certain maize insect pests. The conclusions consistently confirm an increased yield compared to the cultivation of conventional maize [39, 45–47].

Stalk lodging happens more frequently to maize plants infested with corn borers. Lodged plants may escape harvest at the end of the growing season and can therefore increase the occurrence of maize volunteers in the subsequent year. The data of the farmer questionnaires confirm that the occurrence of volunteers was reduced in a significant number of fields with MON 810. Increased germination vigor was observed for MON 810 compared to conventional maize, which is considered to be linked to the availability of high-quality germplasm and premium seed treatments when buying MON 810 seeds. This likely leads to the observation of an increased germination vigor for MON 810 seeds compared to conventional maize seeds.

Finally, MON 810 had reduced susceptibility to disease and pests when compared to conventional maize. Research has demonstrated that insect-damaged maize plants are an easy target for pathogens, such as *Fusarium* [48, 49]. The farmer questionnaires demonstrate that MON 810 maize with its plant-incorporated insect protection is less susceptible to these pathogens. Several reports [48–50] highlighted the beneficial effect of reduction of mycotoxins in the harvested crop, resulting from preventing secondary diseases such as *Fusarium* by limiting insect damage.

Other monitoring characteristics related to environment and wildlife revealed no significant difference between MON 810 maize fields and conventional maize fields, as expected given the nature of MON 810 maize.

The essential characteristics of changes in pest control practice, in particular for maize borer control, and increased yield remained stable over the ten-year period. The minor trends observed in certain other monitoring characteristics may have resulted from farmers adapting their agronomic practice based on experience with MON 810: confidence in efficient pest protection, improved plant development and reduced losses and may have resulted in a later planting. In addition, as farmers gain familiarity with the crop, the “as usual” category is expected to increase for many monitoring characteristics. In any case, the trends are minor and do not significantly influence the observed differences reported above.

The searches of peer-reviewed literature broaden the scope beyond the agricultural setting and the defined territory. The literature searches identified a comprehensive set of publications addressing environmental safety as well as food and feed safety aspects of MON 810. None of the identified publications challenged the original ERA conclusions, nor did they lead to a change in the conclusions of the initial risk assessment that demonstrated the safety of MON 810. All gathered information confirmed that MON 810 is as safe to human and animal health and the environment as conventional maize, and that there is no adverse impact from the cultivation of MON 810 on biodiversity, abundance, or survival of non-target species.

The EU regulatory framework foresees a maximum authorization period of ten years for the placing of GM products on the market, after which a renewal application needs to be submitted. During this period, PMEM required a substantial effort. MON 810 is the only GM product authorized for cultivation in the EU and therefore the first GM product in the EU that has completed a full ten-year cycle.

CS monitoring is intended to assess alleged development of resistance by the target pests, the only potential adverse effect of the GMO identified in the ERA. For the cultivation of MON 810 in the EU, CS monitoring has focused on detecting resistance in the target pests to Cry1Ab and the effectiveness of the implemented IRM plan. There have been no indications of reduced Cry1Ab susceptibility in the two target pests (ECB and MCB) in the EU. This can largely be attributed to the rather conservative, pro-active and effectively implemented IRM plan for MON 810 [7, 8] in combination with additional factors that further reduce the likelihood of resistance development of the target pests (i.e. use of a high-dose product, the mobility of ECB and MCB populations, and the relatively small-scale MON 810 cultivation in the EU, although there are certain areas in the region where the MON 810 adoption rate is high (Table 2)). The implemented IRM plan accompanying the cultivation of MON 810 is needed to sustain the efficacy of this Bt product in the EU.

GS is intended to identify adverse effects of the GMO or its use on human or animal health or the environment which were not anticipated in the ERA. It has been conducted voluntarily and summarized results provide a unique opportunity to review the validity of the current PMEM approach. In this paper, results were reported from implementing the farmer questionnaire and the searches of relevant scientific literature.

The literature searches constitute to be an important GS component. Automation of the identification step ensures a rapid discovery of new publications, whereas the critical review by internal and external experts ensures an evaluation in relation to the existing ERA and the information supporting it.

The farmer questionnaire component required establishing an intensive program with trained interviewers in order to guarantee the necessary response rate. Even then, the selection of stratified subsamples in an evolving market was a challenge. The farmer questionnaires have so far not identified a case of a MON 810 efficacy performance problem or an unanticipated adverse effect. With a great deal of effort, a large number of “as usual” observations have been collected. Based on these results and the efforts required to obtain them, the farmer questionnaire system is not an efficient option for future GS of MON 810 and is not proportionate to the risk.

An alternative tool to capture relevant data from farmers is a farmer complaint system. Companies that are selling MON 810 varieties have established robust farmer complaint systems, which are available to the entire farming community and consequently are not limited to a subsample. The farmer complaint systems serve as a primary tool to detect unexpected adverse effects such as insect susceptibility shifts, and allow reporting of broader issues related with performance criteria such as germination, crop development, damage due to pests and insects, effects on yield and any kind of unexpected finding. Farmers can report complaints to the seed suppliers about product-related topics via the local sales representatives or customer service routes. An example is the Technical User Guide that accompanies each bag of MON 810 and that includes a section with company and seed association contact details for the farmer’s reference. Although the specifics may differ between seed suppliers, in all cases an internal procedure for verification, analysis and follow up processes is initiated upon receipt of a validated product-specific complaint. This procedure includes on-site inspection by company representatives and additional testing of larval susceptibility to Cry1Ab. To collectively monitor the complaints each company received by farmers on a yearly basis, a system was put in place in 2016 by the member companies of the Asociación Nacional de Obtentores Vegetales (ANOVE) that commercialize MON 810 in Spain. A total of 1,556 farmer complaints were received in 2016, demonstrating its effectiveness. Six of the received complaints were related to efficacy of MON 810. After applying the follow-up procedure for these verified complaints, it was confirmed that none of them were linked to resistance development in the field. In addition, registration holders of any MON 810 variety in Spain are obliged to comply with a two-month monitoring plan that includes inspection of fields to monitor potential resistance development [51, 52]. In line with the results of the questionnaires and literature, the complaint systems have so far not identified a case where an in-depth further assessment of a received complaint was attributed to a MON 810 efficacy performance problem or a non-anticipated effect. The complaint systems allow focus on deviating observations that can be early indicators of adverse effects of MON 810 that were not anticipated in the initial ERA, thereby offering an effective, integrated (as used for all kind of complaints) and more cost-efficient option for MON 810 GS, proportionate to the risk.

## Conclusion

The ten-year analysis of data reported by farmers on 2,627 fields in eight European countries in the period 2006–2015 did not reveal any adverse effects associated with the cultivation of MON 810. Statistically significant differences between the cultivation of conventional maize and of MON 810 observed in the farmer questionnaires can be directly attributed to the insect protection provided by Cry1Ab expressed in MON 810. MON 810 provided tangible and non-tangible benefits and allowed farmers to adapt their crop management as illustrated by some of the trends. It provided flexibility in management while ensuring that the crop was adequately protected against the target pests, a protection that likely spanned beyond the specific fields and provided benefits to non Bt-maize growing farmers.

Furthermore, the searches of peer-reviewed literature, guaranteeing a broad coverage of potential issues, did not reveal any adverse effects that changed the conclusions of the initial risk assessment that demonstrated the safety of MON 810. These results are in line with EFSA’s assessment of the 2007 renewal application, confirming the conclusions of the original safety assessment: “…*MON 810 is as safe as its conventional counterpart with respect to potential effects on human and animal health*. *The EFSA GMO Panel also concludes that maize MON 810 is unlikely to have any adverse effect on the environment in the context of its intended uses…*” [53].

When the concepts of PMEM were developed, the dynamic nature of the approach was stressed. All PMEM guidance documents indicate that monitoring plans should not be viewed as static and that it is fundamental that the monitoring plan and associated methodology are reviewed at appropriate intervals and may need to be adapted depending on the results of the monitoring information collected [4, 54]. The PMEM results for MON 810 offer the first opportunity to evaluate the contribution of different components. Even though no resistance of ECB or MCB to the Cry1Ab protein has been detected in the EU, continued IRM efforts are needed to sustain product efficacy and delay the development of resistance. On the other hand, while requiring an intensive effort and facing organizational challenges, the farmer questionnaires did not reveal any new finding that could not have been revealed via the farmer complaint system. The results presented in this publication are consistent with the history of safe use of MON 810 for nearly 15 years in the EU. Therefore, this publication suggests that focussing the GS component of the accompanying PMEM of MON 810 to literature searches and the farmer complaint systems would not weaken the level of protection for human or animal health and the environment.

## Supporting information

S1 File Statistical background. (DOCX)

S2 File Results of the statistical analysis of the farmer questionnaires. (DOCX)

S3 File Results of the statistical analysis of the farmer questionnaires (Graph bars). (DOCX)

S4 File Results of the literature screening. (XLSX)

S5 File References of literature screening. (DOCX)

S6 File Keywords used for the literature searches. (DOCX)

S7 File Results of the individual farmer questionnaires. (XLSX)
