# Supplementary material for: Results from ten years of post-market environmental monitoring of genetically modified MON 810 maize in the European Union
Source: PLoS One. 2020 Apr 24;15(4):e0217272. doi: 10.1371/journal.pone.0217272 (PMC7182268; doi:10.1371/journal.pone.0217272)
Supplement: S1 File — (DOCX) [file pone.0217272.s001.docx]

**S1 File. Statistical background**

[Closed Test Procedure 1](#_Toc478390465)

[Sample size determination 3](#_Toc478390466)

[Descriptive and Meta-analysis of the 10-years pooled data 6](#_Toc478390467)

[Trend analysis 8](#_Toc478390468)

[References 9](#_Toc478390469)

## Closed Test Procedure

The frequencies of *as usual-,* and *different-* (*i.e.* *plus*- or *minus*-) answers are statistically tested according to the closed principle test procedure (in case of questions that allow for only two answers like *e.g.* *Crop Rotation’s* “as usual”/”changed”, only *as usual*- and *plus*-answer frequencies are tested accordingly).

The categories *As usual*, *Plus* and *Minus* form a vector with a multinomial distribution

$$\left(Minus,As usual, Plus\right)\sim Mult\left(n;p_{Minus},p_{As usual},p_{Plus}\right)$$

Therefore, each component of this vector is binomially distributed

$Minus\sim B\left(n,p_{Minus},k\right)$, $As usual\sim B\left(n,p_{As usual},k\right)$, $Plus\sim B\left(n,p_{Plus},k\right)$

To detect an effect of MON810 cultivation, the following statistical hypothesis are formulated:

$H_{0}^{1}:p_{As usual}\leq0.9$ vs. $H_{A}^{1}:p_{As usual}>0.9$

$H_{0}^{2}:p_{Minus}\geq0.1$ vs. $H_{A}^{2}:p_{Minus}<0.1$

$H_{0}^{3}:p_{Plus}\geq0.1$ vs. $H_{A}^{3}:p_{Plus}<0.1$

The set of null hypothesis $\left\{H_{0}^{1},H_{0}^{2},H_{0}^{3}\right\}$ is closed under intersection because

$H_{0}^{1}\cap H_{0}^{2}=\left[0,0.9\right]\cap\left[0.1,1\right]=\left[0.1,0.9\right]\in\left[0,1\right]=\left\{H_{0}^{1},H_{0}^{2},H_{0}^{3}\right\}$ and

$H_{0}^{1}\cap H_{0}^{3}=\left[0,0.9\right]\cap\left[0.1,1\right]=\left[0.1,0.9\right]\in\left[0,1\right]=\left\{H_{0}^{1},H_{0}^{2},H_{0}^{3}\right\}$ and

$H_{0}^{2}\cap H_{0}^{3}=\left[0.1,1\right]\cap\left[0.1,1\right]=\left[0.1,1\right]\in\left[0,1\right]=\left\{H_{0}^{1},H_{0}^{2},H_{0}^{3}\right\}$.

The detection of an effect is made in two steps. First, the global null hypothesis $H_{0}^{1}:p_{As usual}\leq0.9$ is tested. If this hypothesis is rejected, testing of the hypotheses $H_{0}^{2}$ and $H_{0}^{3}$ is not needed anymore since they will be rejected then, too. Secondly, if $H_{0}^{1}:p_{As usual}\leq0.9$ is not rejected, the hypotheses $H_{0}^{2}$ and $H_{0}^{3}$ are to be tested. The test procedure is displayed in Fig. A.

Fig A. Closed test procedure for the three probabilities of $As usual$, $Plus$- and $Minus$-answers

This test procedure is coherent because a rejection of the null hypothesis in step 1 implies a rejection of the hypotheses in step 2. The test procedure is called a closed test procedure.

Within the closed test principle, hypotheses are tested by applying the exact binomial test.

- Step (1): Test of the probability $p_{As usual}$ (usually the largest probability)

Null hypothesis: GMP cultivation has an effect, the probability of getting $As usual$-answers is smaller than 90 % ($H_{0}: p_{As usual}\leq0.9$)

- Step (2): Test of the $p_{Minus}$ probabilities and $p_{Plus}$ probabilities

Null hypothesis: GMP cultivation has an effect, the probability of getting $Minus$- or $Plus$-answers is larger than 10 % ( $H_{0}: p_{Minus}\geq0.1$, $H_{0}: p_{Plus}\geq0.1$)

The resulting P-values are compared to a level of significance α=0.01. If the P-value is smaller than α=0.01, the corresponding null hypothesis is rejected. If the P-value is larger than α=0.01, respective hypothesis cannot be rejected.

- In case Hypothesis (1) with $p_{As usual}\leq0.9$ is rejected, no effect is indicated.
- In case Hypothesis (1) with $p_{As usual}\leq0.9$ cannot be rejected, but both hypotheses (2) with $p_{Minus}\geq0.1$ and $p_{Plus}\geq0.1$ can be rejected, no effect is indicated.
- In case Hypothesis (1) with $p_{As usual}\leq0.9$ cannot be rejected and at least one of the hypotheses (2) cannot be rejected either, an effect is indicated.

This closed test procedure controls for the experiment-wise error rate because an erroneous decision, *i.e.* an error of the first kind (rejection of the null hypothesis although it is true) during the whole procedure can only be done once: an erroneous rejection of the null hypothesis (1) (*i.e.* in reality $p_{As usual}\leq0.9$) corresponds to an erroneous rejection of the null hypotheses (2) (*i.e.* in reality $p_{Plus}\geq0.1$ or $p_{Minus}\geq0.1$) [Marcus, 1976], [Maurer, 1995].

## Sample size determination

The sample size determination of the survey was based on the one-sided exact binomial test, comparison of a probability with a constant. It depends on the constant, the error of the first kind $\alpha$, the error of the second kind $\beta$ and the minimum difference of practical interest $d$ [Rasch, 2007a].

The error of the first kind is the probability to reject the null hypothesis although it is true, *i.e.* not to identify an existing effect. This probability should be as small as possible since it is the aim of GS to identify any existing effects. The error of the first kind is also called consumer's risk.

The error of the second kind is the probability to accept the null hypothesis although it is false, *i.e.* to identify an effect although none exists. This probability should also be as small as possible as it would raise false alarm (Table A). The error of the second kind is also called producer's risk.

The magnitude of the minimum difference of practical interest $d$ specifies the minimum difference value behind the '<' and '>' in the null hypotheses $H_{0}^{1}:p_{As usual}\leq0.9$, $H_{0}^{2}:p_{Minus}\geq0.1$ or $H_{0}^{3}:p_{Plus}\geq0.1$, respectively. It was chosen from experience in analyzing farm questionnaires in a pilot study in Germany 2001 - 2005 [Schmidt, 2008].

|  | | **Real situation** | |
| --- | --- | --- | --- |
|  |  | $p\leq0.9$ **Indication for an effect** | $p>0.9$ **No effect** |
| **Test decision** | **Acceptance** $H_{0} : p\leq0.9$ | Correct decision with  Probability $1-\alpha= 99 \%$ | Wrong decision with  Probability $\beta=1 \%$ |
|  | **Rejection** $H_{0} : p\leq0.9$ | Wrong decision with  Probability $\alpha=1 \%$ | Correct decision with  Probability $1-\beta= 99 \%$ $= POWER$ |

Table A: Error of the first kind $\alpha$ and error of the second kind $\beta$ for the test decision in testing frequencies of $Plus$- or $Minus$-answers from farm questionnaires against the threshold of 10 %

CADEMO light [1] was used as proposed by [2] to determine the sample size for a binomial test (Method 3/62/1005). A sample size of 2 436 questionnaires results from the accuracy demands $p = 0.9$ (threshold for effects to be tested: 90 % of *as usual*-answers, α = 0.01 (error of the first kind), β = 0.01 (error of the second kind), and d 0 0.03 (minimum difference of practical interest).

The power of the test $p_{Minus}\geq0.1$, $p_{Plus}\geq0.1$, respectively is the probability to reject the null hypothesis of an effect where none exists (correct decision). It is defined as $1-\beta$ ($\beta$ = error of the second kind) and is calculated as followed:

$$Power= \sum_{F=0}^{F_{u}-1} \left( \frac{n!}{F!\left( n-F \right)!} \right)p^{F}{(1-p)}^{n-F}$$

where:

$F_{u}= \min_{F} (P(F\leq F_{E}|H_{0})>\alpha$

$p=$ given probability of $Plus$- or $Minus$ -answers for which the power is calculates

$F_{E}=$ absolute frequency of $Plus$- or $Minus$ -answers

Fig B illustrates the power for an alternative hypothesis value of 0.13 (minimum difference of practical interest 0.03). The distribution of the null hypothesis value (0.10) is represented by the red curve; the distribution of the alternative hypothesis value (0.13) is represented by the blue curve. The green line shows the critical value for an error probability $\alpha=0.01$. If the alternative hypothesis is actually true (GM cultivation has no effect) the rejection of the null hypothesis is a correct decision which will occur with 99 % probability (under the blue curve to the left of the green line), *i.e.* with a power of 99 %.

Fig B. Null ($p = 0.1$) and alternative ($p = 0.13$) binomial distribution functions for a sample size of 2 500 type I and type II errors $\alpha$ and $\beta$ both 0.01 (graph: G*Power Version 3.1.6)

## Descriptive and Meta-analysis of the 10-years pooled data

### Descriptive analysis of the sample

In the descriptive analysis of the pooled data, ***simple*** ***proportions p^s^*** of the *as usual*-, *plus*- and *minus-* categories of the monitoring characteristics were estimated.

$$p_{country_{i};year_{j}}^{s}=\frac{n_{country_{i};year_{j}}}{N_{country_{i};year_{j}}}$$

$$p_{year_{j}}^{s}=\frac{n_{year_{j}}}{N_{year_{j}}}$$

$$p^{s}=\frac{n}{N}$$

*(j = 2006,..., 2015, i = Czech Republic, France, ..., Spain)*

where

| $p^{s}$ | total simple proportion (for *as usual*-, *plus*- or *minus-* category) |
| --- | --- |
| $p_{year_{j}}^{s}$ | simple proportion (for *as usual*-, *plus*- or *minus-* category) in year *j* |
| $p_{country_{i};year_{j}}^{s}$ | simple proportion (for *as usual*-, *plus*- or *minus-* category) in country *i* and year *j* |
| *n* | total number of positive responses (for *as usual*-, *plus*- or *minus-* category) |
| *N* | total sample size |
| $n_{year_{j}}$ | number of positive responses (for *as usual*-, *plus*- or *minus-* category) in year *j* |
| $N_{year_{j}}$ | sample size in year *j* |
| $n_{country_{i};year_{j}}$ | number of positive responses (for *as usual*-, *plus*- or *minus-* category) in county *i* and year *j* |
| $N_{country_{i};year_{j}}$ | sample size in country *i* and year *j* |

Because of the irregular distribution of the sample across the strata, also ***weighted*** ***proportions p^w^*** of the *as usual*-, *plus*- and *minus-* categories of the monitoring characteristics were estimated: from the simple proportions per country and year $p_{country;year_{j}}^{s}$, weighted mean proportions per year $p_{year_{j}}^{w}$ were calculated. Weighting was based on the ratio 'sample size in country/ total sample size (per year)'. Finally, the total weighted proportions $p^{w}$ (averaged over all years) were calculated.

$$p_{year_{j}}^{w}=\sum_{i} \frac{N_{country_{i};year_{j}}}{N_{year_{j}}}p_{country_{i};year_{j}}^{s}$$

$$p^{w}=\sum_{j} \frac{p_{year_{j}}^{w}}{M}$$

where

| $p^{w}$ | total weighted proportion (for *as usual*-, *plus*- or *minus-* category) | |
| --- | --- | --- |
| $p_{year_{j}}^{w}$ | weighted proportion (for *as usual*-, *plus*- or *minus-* category) in year *j* | |
| $p_{country_{i};year_{j}}^{s}$ | | simple proportion (for *as usual*-, *plus*- or *minus-* category) in country and year *j*, see above |
| $N_{country_{i},year_{j}}$ | sample size in country *i* and year *j* | |
| $N_{year_{j}}$ | sample size in year *j* | |
| $M$ | number of years in which the monitoring characteristic was measured (normally M=10 (2006 - 2015), for Crop rotation, Occurrence of insects, birds and mammals: M=7 (2009 - 2015), for Maize Borer control practice: M=8 (2008 - 2015) | |

### Meta-analysis of monitoring characteristics

Comparable to the yearly analyses, the meta-analysis (i.e. the analysis of the combined 10 years of data) of monitoring characteristics was carried out in two steps.

1. Parameter estimation

Each of the categories *as usual*, *plus* and *minus* were considered separately. For each of the categories the farmers' answers were transformed to resp=0 or resp=1. Then, applying a linear mixed model, the ***model proportions***  ***p^mo^*** of the *as usual*-, *plus*- and *minus-* categories were estimated. A cross-classified three-factorial model (strata = fixed factors year and country as well as random factor 'multiple participation of farmers') with interactions

$$\begin{matrix} resp_{ijkl}=p^{mo}+country_{i}+year_{j}+multiple_{l}+country\bullet year_{ij}+country\bullet multiple_{il} \\ +year\bullet multiple_{jl}+country\bullet year\bullet multiple_{ijl}+\varepsilon_{ijkl} \end{matrix}$$

because of the irregular pattern of the sampling frame (and consequently of the sample), was unable to fit the data and to converge on model parameters. A three-factorial model without interactions resulted in a non-positive Hessian matrix, implying a sub-optimal iteration result. In this model the estimated variances of the random effect 'multiple participation of farmers' were either Zero or negligible. Therefore this factor was removed and finally a two-factorials model (factors year and country) without interactions was applied (the model with interactions did not converge for all monitoring characters).

$$resp_{ijk}=p^{mo}+country_{i}+year_{j}+\varepsilon_{ijk}$$

Within the model, the total model proportions (inclusive 99% confidence intervals) as well as the year-specific (marginal) model proportions were estimated (SPSS MIXED procedure, Least square means).

$$p_{year_{j}}^{mo}=p^{mo}+year_{j}$$

where

| $p^{mo}$ | total model proportion (for *as usual*-, *plus*- or *minus-* category) |
| --- | --- |
| $\text{p}_{\text{year}_{\text{i}}}^{\text{mo}}$ | model proportion (for *as usual*-, *plus*- or *minus-* category) in year i |
| $resp_{ijkl}$ | response of the *k*-th farmer from country *i* in his *l*-th participation in year *j* |
| $country_{i}$ | effect of the *i*-th country (fixed effect) |
| $year_{j}$ | effect of the *j*-th year (fixed effect) |
| $multi_{l}$ | effect of the *l*-th participation of a farmer (random effect) |
| $country\bullet year_{ij}$ | twofold interaction between the *i*-th country and the *j*-th year |
| $country\bullet multiple_{il}$ | twofold interaction between the *i*-th country and the *l*-th participation of a farmer |
| $year\bullet multiple_{jl}$ | twofold interaction between the *j*-th year and the *l*-th participation of a farmer |
| $country\bullet year\bullet multiple_{ijl}$ | threefold interaction between the *i*-th country, the *j*-th year and the *l*-th participation |
| $\varepsilon_{ijkl}$ | error |

*p^mo^* is the estimated total model proportion of the corresponding *as usual*-, *plus*- or *minus-* response category. $\text{p}_{\text{yearj}}^{\text{mo}}$ are the year-specific model proportions of the *as usual*-, *plus*- or *minus-* response categories.

2. Parameter test

Secondly, the estimated 99% confidence intervals for the model proportions ***p^mo^*** were compared with the defined thresholds: (1) $p_{\text{as usual}}\geq0.9$, and (2) $p_{\text{minus}}\leq0.1$ or $p_{\text{plus}}\leq0.1$. Intervals for *as usual* that were strictly <0.9 and intervals for *plus* or *minus* that were strictly >0.1 were indicative of possible effects.

## Trend analysis

Additionally, for each monitoring characteristic the estimated year-specific model proportions $\text{p}_{\text{year}_{\text{j}}}^{\text{mo}}$ of the *as usual*-, *plus*- or *minus-* response categories were assessed for any linear trends over years by fitting linear regression models and subsequently testing the estimated slopes $\beta_{2}$ against zero [3]:

$p_{\text{year}_{\text{j}}\text{ }}^{mo}=\beta_{1}+\beta_{2}\cdot year_{\text{j}}+\varepsilon_{year_{j}}$ where

| $\text{p}_{\text{year}_{\text{j}}}^{\text{mo}}$ | estimated *j*-th year model proportion (for *as usual*-, *plus*- or *minus-* category) |
| --- | --- |
| $\beta_{1}$ | absolute term of the linear model |
| $\beta_{2}$ | slope (extent of the trend) of the linear model |
| *year_j_* | year *j* (time point, *j = 2006,..., 2015*) |
| $\varepsilon_{year_{j}}$ | error |

To check for a linear trend, the slope of the linear function $\beta_{2}$ was tested as follows.

$H_{0}: \beta_{2}=0$ against $H_{A}: \beta_{2}\neq0$

A trend with strength $\beta_{2}$ was deemed to exist if $H_{0}$ was rejected.

To test the slope $\beta_{2}$, confidence intervals with confidence level $\alpha=0.05$ were applied, and $H_{0}$ was rejected if the confidence interval did not include the zero.

References

[1] CADEMO Manual. Release 3.13; 2000.

[2] D. Rasch, G. Herrendörfer, J. Bock, N. Victor, V.Guiard. Verfahrensbibliothek: Versuchsplanung und -auswertung. Oldenbourg Verlag München 2007.

[3] Diggle P. Time series: A biostatistical introduction 1990.
