# Supplementary material for: Results from ten years of post-market environmental monitoring of genetically modified MON 810 maize in the European Union
Source: PLoS One. 2020 Apr 24;15(4):e0217272. doi: 10.1371/journal.pone.0217272 (PMC7182268; doi:10.1371/journal.pone.0217272)
Supplement: S2 File — (DOCX) [file pone.0217272.s002.docx]

**S2 File. Results of the statistical analysis of the farmer questionnaires**

Tables: simple, weighted, model proportions and slopes

## Table A: Descriptive *simple* *proportions p* of the *as usual*- categories of the monitoring characteristics (Table values are formatted as p*100)

| **Subject** | **Monitoring characteristic** | **2006** | **2007** | **2008** | **2009** | **2010** | **2011** | **2012** | **2013** | **2014** | **2015** | **Total** |
| --- | --- | --- | --- | --- | --- | --- | --- | --- | --- | --- | --- | --- |
| Agronomic practices | Crop rotation |  |  |  | 99.17 | 98.15 | 99.20 | 95.58 | 94.14 | 96.17 | 93.49 | 96.56 |
|  | Time of planting | 92.43 | 92.78 | 94.61 | 95.83 | 94.10 | 97.19 | 96.39 | 94.92 | 95.40 | 93.10 | 94.68 |
|  | Tillage and planting technique | 100.00 | 99.31 | 100.00 | 100.00 | 99.63 | 100.00 | 97.99 | 98.05 | 96.93 | 96.54 | 98.85 |
|  | Insect control practices | 52.00 | 88.11 | 77.78 | 81.67 | 84.13 | 75.10 | 82.73 | 83.59 | 83.52 | 85.44 | 79.41 |
|  | Fertilizer application | 99.20 | 99.66 | 100.00 | 99.58 | 99.63 | 100.00 | 100.00 | 97.66 | 99.62 | 100.00 | 99.53 |
|  | Time of harvest | 73.49 | 77.66 | 82.83 | 90.00 | 91.14 | 95.18 | 95.98 | 94.92 | 95.02 | 95.77 | 89.20 |
|  | Weed control practices | 99.59 | 99.66 | 99.66 | 100.00 | 99.63 | 100.00 | 100.00 | 100.00 | 98.08 | 100.00 | 99.66 |
|  | Fungal control practices | 100.00 | 98.94 | 100.00 | 99.58 | 100.00 | 100.00 | 100.00 | 100.00 | 100.00 | 100.00 | 99.85 |
|  | Irrigation Practices | 98.36 | 100.00 | 100.00 | 100.00 | 99.63 | 100.00 | 100.00 | 100.00 | 100.00 | 100.00 | 99.80 |
|  | Maize Borer control practice |  |  | 90.16 | 77.08 | 84.50 | 77.11 | 81.93 | 83.98 | 83.91 | 85.77 | 83.06 |
| Characteristics in the field | Germination vigor | 86.00 | 89.00 | 86.87 | 84.52 | 83.76 | 94.38 | 93.98 | 91.80 | 88.12 | 86.97 | 88.54 |
|  | Time to emergence | 87.50 | 93.10 | 91.58 | 93.72 | 95.57 | 99.20 | 98.80 | 99.61 | 100.00 | 99.62 | 95.87 |
|  | Time to male flowering | 98.00 | 90.63 | 91.55 | 96.23 | 93.73 | 97.99 | 97.99 | 98.83 | 99.62 | 100.00 | 96.46 |
|  | Plant growth and development | 91.84 | 88.28 | 87.50 | 92.05 | 89.30 | 98.39 | 96.39 | 98.05 | 100.00 | 98.47 | 94.03 |
|  | Incidence of stalk / root lodging | 39.52 | 63.57 | 61.09 | 68.07 | 62.73 | 75.50 | 71.89 | 82.81 | 73.18 | 72.80 | 67.12 |
|  | Time to maturity | 67.07 | 69.42 | 71.65 | 82.43 | 79.70 | 87.15 | 83.94 | 87.50 | 88.51 | 93.87 | 81.12 |
|  | Yield | 28.86 | 51.42 | 42.91 | 41.42 | 48.34 | 56.63 | 54.62 | 63.28 | 62.45 | 49.43 | 49.94 |
|  | Occurrence of volunteers | 66.09 | 89.89 | 88.94 | 89.23 | 91.42 | 93.06 | 96.20 | 96.00 | 98.85 | 95.79 | 90.55 |
| Environment and wildlife | Disease susceptibility | 61.90 | 77.32 | 64.65 | 70.29 | 74.07 | 80.32 | 82.73 | 87.45 | 94.64 | 95.79 | 78.92 |
|  | Pest susceptibility | 87.70 | 92.76 | 80.81 | 81.51 | 81.41 | 82.33 | 78.31 | 81.57 | 83.52 | 78.16 | 82.81 |
|  | Weed pressure | 99.60 | 97.59 | 97.98 | 97.91 | 94.83 | 100.00 | 100.00 | 99.61 | 100.00 | 100.00 | 98.75 |
|  | Performance of animals | 100.00 | 93.33 | 95.06 | 91.07 | 87.72 | 89.47 | 90.00 | 92.31 | 100.00 | 100.00 | 93.90 |
|  | Occurrence of insects |  |  |  | 98.27 | 98.86 | 98.72 | 100.00 | 100.00 | 100.00 | 100.00 | 99.41 |
|  | Occurrence of birds |  |  |  | 99.57 | 98.06 | 99.58 | 100.00 | 100.00 | 99.62 | 100.00 | 99.55 |
|  | Occurrence of mammals |  |  |  | 97.84 | 97.71 | 99.16 | 99.59 | 100.00 | 99.62 | 100.00 | 99.13 |

## Table B: Descriptive *simple* *proportions p* of the *plus*- or *changed*- categories of the monitoring characteristics (Table values are formatted as p*100)

| **Subject** | **Monitoring characteristic** | **2006** | **2007** | **2008** | **2009** | **2010** | **2011** | **2012** | **2013** | **2014** | **2015** | **Total** |
| --- | --- | --- | --- | --- | --- | --- | --- | --- | --- | --- | --- | --- |
| Agronomic practices | Crop rotation (changed) |  |  |  | 0.83 | 1.85 | 0.80 | 4.42 | 5.86 | 3.83 | 6.51 | 3.44 |
|  | Time of planting (later) | 5.98 | 3.78 | 2.69 | 1.25 | 4.06 | 1.61 | 3.61 | 5.08 | 4.21 | 6.51 | 3.88 |
|  | Tillage and planting technique (changed) | 0.00 | 0.69 | 0.00 | 0.00 | 0.37 | 0.00 | 2.01 | 1.95 | 3.07 | 3.46 | 1.15 |
|  | Insect control practices (changed) | 48.00 | 11.89 | 22.22 | 18.33 | 15.87 | 24.90 | 17.27 | 16.41 | 16.48 | 14.56 | 20.59 |
|  | Fertilizer application (changed) | 0.80 | 0.34 | 0.00 | 0.42 | 0.37 | 0.00 | 0.00 | 2.34 | 0.38 | 0.00 | 0.47 |
|  | Time of harvest (later) | 24.10 | 18.56 | 13.80 | 7.92 | 6.64 | 4.42 | 4.02 | 5.08 | 4.60 | 4.23 | 9.34 |
|  | Weed control practices (changed) | 0.41 | 0.34 | 0.34 | 0.00 | 0.37 | 0.00 | 0.00 | 0.00 | 1.92 | 0.00 | 0.34 |
|  | Fungal control practices (changed) | 0.00 | 1.06 | 0.00 | 0.42 | 0.00 | 0.00 | 0.00 | 0.00 | 0.00 | 0.00 | 0.15 |
|  | Irrigation Practices (changed) | 1.64 | 0.00 | 0.00 | 0.00 | 0.37 | 0.00 | 0.00 | 0.00 | 0.00 | 0.00 | 0.20 |
|  | Maize Borer control practice (changed) |  |  | 9.84 | 22.92 | 15.50 | 22.89 | 18.07 | 16.02 | 16.09 | 14.23 | 16.94 |
| Characteristics in the field | Germination vigor (more) | 8.00 | 6.87 | 11.45 | 14.64 | 16.24 | 5.62 | 5.62 | 7.42 | 11.88 | 13.03 | 10.08 |
|  | Time to emergence (delayed) | 5.65 | 3.79 | 2.02 | 0.84 | 0.37 | 0.00 | 0.40 | 0.39 | 0.00 | 0.00 | 1.35 |
|  | Time to male flowering (delayed) | 1.60 | 7.64 | 3.72 | 1.67 | 2.58 | 2.01 | 1.20 | 0.39 | 0.38 | 0.00 | 2.12 |
|  | Plant growth and development (delayed) | 1.63 | 4.83 | 2.70 | 2.09 | 3.69 | 0.80 | 2.01 | 0.78 | 0.00 | 0.38 | 1.89 |
|  | Incidence of stalk / root lodging (more) | 1.61 | 0.34 | 0.34 | 0.00 | 2.21 | 0.00 | 0.00 | 0.00 | 0.00 | 0.00 | 0.45 |
|  | Time to maturity (delayed) | 30.92 | 25.77 | 24.02 | 14.64 | 16.24 | 12.85 | 16.06 | 12.50 | 11.49 | 6.13 | 17.06 |
|  | Yield (higher) | 68.70 | 44.68 | 52.70 | 56.90 | 49.82 | 43.37 | 42.97 | 34.77 | 36.02 | 50.57 | 48.05 |
|  | Occurrence of volunteers (more) | 0.00 | 1.69 | 0.00 | 0.00 | 0.43 | 0.00 | 0.00 | 0.00 | 0.00 | 0.38 | 0.25 |
| Environment and wildlife | Disease susceptibility (more) | 1.98 | 1.03 | 0.67 | 0.42 | 0.37 | 0.00 | 0.00 | 0.00 | 0.00 | 0.00 | 0.45 |
|  | Pest susceptibility (more) | 1.19 | 1.38 | 0.67 | 1.26 | 0.00 | 0.00 | 0.40 | 0.39 | 0.38 | 0.00 | 0.57 |
|  | Weed pressure (more) | 0.00 | 0.34 | 0.34 | 0.00 | 0.37 | 0.00 | 0.00 | 0.39 | 0.00 | 0.00 | 0.14 |
|  | Performance of animals (changed) | 0.00 | 6.67 | 4.94 | 8.93 | 12.28 | 10.53 | 10.00 | 7.69 | 0.00 | 0.00 | 6.10 |
|  | Occurrence of insects (more) |  |  |  | 0.87 | 0.38 | 0.43 | 0.00 | 0.00 | 0.00 | 0.00 | 0.24 |
|  | Occurrence of birds (more) |  |  |  | 0.00 | 0.78 | 0.00 | 0.00 | 0.00 | 0.00 | 0.00 | 0.11 |
|  | Occurrence of mammals (more) |  |  |  | 1.29 | 1.15 | 0.42 | 0.00 | 0.00 | 0.00 | 0.00 | 0.41 |

## Table C: Descriptive *simple* *proportions p* of the *minus*- categories of the monitoring characteristics (Table values are formatted as p*100)

| **Subject** | **Monitoring characteristic** | **2006** | **2007** | **2008** | **2009** | **2010** | **2011** | **2012** | **2013** | **2014** | **2015** | **Total** |
| --- | --- | --- | --- | --- | --- | --- | --- | --- | --- | --- | --- | --- |
| Agronomic practices | Time of planting (earlier) | 1.59 | 3.44 | 2.69 | 2.92 | 1.85 | 1.20 | 0.00 | 0.00 | 0.38 | 0.38 | 1.45 |
|  | Time of harvest (earlier) | 2.41 | 3.78 | 3.37 | 2.08 | 2.21 | 0.40 | 0.00 | 0.00 | 0.38 | 0.00 | 1.46 |
| Characteristics in the field | Germination vigor (less) | 6.00 | 4.12 | 1.68 | 0.84 | 0.00 | 0.00 | 0.40 | 0.78 | 0.00 | 0.00 | 1.38 |
|  | Time to emergence (accelerated) | 6.85 | 3.10 | 6.40 | 5.44 | 4.06 | 0.80 | 0.80 | 0.00 | 0.00 | 0.38 | 2.78 |
|  | Time to male flowering (accelerated) | 0.40 | 1.74 | 4.73 | 2.09 | 3.69 | 0.00 | 0.80 | 0.78 | 0.00 | 0.00 | 1.42 |
|  | Plant growth and development (accelerated) | 6.53 | 6.90 | 9.80 | 5.86 | 7.01 | 0.80 | 1.61 | 1.17 | 0.00 | 1.15 | 4.08 |
|  | Incidence of stalk / root lodging (less) | 58.87 | 36.08 | 38.57 | 31.93 | 35.06 | 24.50 | 28.11 | 17.19 | 26.82 | 27.20 | 32.43 |
|  | Time to maturity (accelerated) | 2.01 | 4.81 | 4.33 | 2.93 | 4.06 | 0.00 | 0.00 | 0.00 | 0.00 | 0.00 | 1.81 |
|  | Yield (lower) | 2.44 | 3.90 | 4.39 | 1.67 | 1.85 | 0.00 | 2.41 | 1.95 | 1.53 | 0.00 | 2.01 |
|  | Occurrence of volunteers (less) | 33.91 | 8.43 | 11.06 | 10.77 | 8.15 | 6.94 | 3.80 | 4.00 | 1.15 | 3.83 | 9.20 |
| Environment and wildlife | Disease susceptibility (less) | 36.11 | 21.65 | 34.68 | 29.29 | 25.56 | 19.68 | 17.27 | 12.55 | 5.36 | 4.21 | 20.64 |
|  | Pest susceptibility (less) | 11.11 | 5.86 | 18.52 | 17.23 | 18.59 | 17.67 | 21.29 | 18.04 | 16.09 | 21.84 | 16.62 |
|  | Weed pressure (less) | 0.40 | 2.06 | 1.68 | 2.09 | 4.80 | 0.00 | 0.00 | 0.00 | 0.00 | 0.00 | 1.10 |
|  | Occurrence of insects (less) |  |  |  | 0.87 | 0.76 | 0.85 | 0.00 | 0.00 | 0.00 | 0.00 | 0.35 |
|  | Occurrence of birds (less) |  |  |  | 0.43 | 1.16 | 0.42 | 0.00 | 0.00 | 0.38 | 0.00 | 0.34 |
|  | Occurrence of mammals (less) |  |  |  | 0.86 | 1.15 | 0.42 | 0.41 | 0.00 | 0.38 | 0.00 | 0.46 |

## Table D: Descriptive *weighted* *proportions p^w^* of the *as usual*- categories of the monitoring characteristics (Table values are formatted as p*100)

| **Subject** | **Monitoring characteristic** | **2006** | **2007** | **2008** | **2009** | **2010** | **2011** | **2012** | **2013** | **2014** | **2015** | **Total** |
| --- | --- | --- | --- | --- | --- | --- | --- | --- | --- | --- | --- | --- |
| Agronomic practices | Crop rotation |  |  |  | 99.17 | 98.15 | 99.20 | 95.58 | 94.14 | 96.17 | 93.49 | 96.56 |
|  | Time of planting | 92.45 | 92.78 | 94.61 | 95.83 | 94.10 | 97.19 | 96.39 | 94.92 | 95.40 | 93.10 | 94.68 |
|  | Tillage and planting technique | 100.00 | 99.31 | 100.00 | 100.00 | 99.63 | 100.00 | 97.99 | 98.05 | 96.93 | 96.54 | 98.85 |
|  | Insect control practices | 52.49 | 88.11 | 77.78 | 81.67 | 84.13 | 75.10 | 82.73 | 83.59 | 83.52 | 85.44 | 79.46 |
|  | Fertilizer application | 99.20 | 99.66 | 100.00 | 99.58 | 99.63 | 100.00 | 100.00 | 97.66 | 99.62 | 100.00 | 99.53 |
|  | Time of harvest | 73.44 | 77.66 | 82.83 | 90.00 | 91.14 | 95.18 | 95.98 | 94.92 | 95.02 | 95.79 | 89.20 |
|  | Weed control practices | 99.59 | 99.66 | 99.66 | 100.00 | 99.63 | 100.00 | 100.00 | 100.00 | 98.08 | 100.00 | 99.66 |
|  | Fungal control practices | 100.00 | 98.95 | 100.00 | 99.58 | 100.00 | 100.00 | 100.00 | 100.00 | 100.00 | 100.00 | 99.85 |
|  | Irrigation Practices | 94.24 | 100.00 | 100.00 | 100.00 | 99.63 | 100.00 | 100.00 | 100.00 | 100.00 | 100.00 | 99.39 |
|  | Maize Borer control practice |  |  | 90.16 | 77.08 | 84.50 | 77.11 | 81.93 | 83.98 | 83.91 | 85.81 | 83.06 |
| Characteristics in the field | Germination vigor | 86.07 | 89.00 | 86.87 | 84.52 | 83.76 | 94.38 | 93.98 | 91.80 | 88.12 | 86.97 | 88.55 |
|  | Time to emergence | 87.61 | 93.11 | 91.58 | 93.69 | 95.57 | 99.20 | 98.80 | 99.61 | 100.00 | 99.62 | 95.88 |
|  | Time to male flowering | 98.00 | 90.64 | 91.52 | 96.23 | 93.73 | 97.99 | 97.99 | 98.83 | 99.62 | 100.00 | 96.45 |
|  | Plant growth and development | 91.80 | 88.28 | 87.49 | 92.05 | 89.30 | 98.39 | 96.39 | 98.05 | 100.00 | 98.47 | 94.02 |
|  | Incidence of stalk / root lodging | 39.62 | 63.57 | 60.98 | 68.03 | 62.73 | 75.50 | 71.89 | 82.81 | 73.18 | 72.80 | 67.11 |
|  | Time to maturity | 67.11 | 69.42 | 71.65 | 82.32 | 79.70 | 87.15 | 83.94 | 87.50 | 88.51 | 93.87 | 81.12 |
|  | Yield | 29.25 | 51.68 | 42.89 | 41.41 | 48.34 | 56.63 | 54.62 | 63.28 | 62.45 | 49.43 | 50.00 |
|  | Occurrence of volunteers | 72.22 | 85.76 | 84.05 | 88.14 | 89.42 | 93.03 | 96.39 | 96.09 | 98.85 | 95.79 | 89.97 |
| Environment and wildlife | Disease susceptibility | 61.90 | 77.32 | 64.65 | 70.12 | 73.99 | 80.32 | 82.73 | 87.27 | 94.64 | 95.79 | 78.87 |
|  | Pest susceptibility | 87.70 | 92.78 | 80.81 | 81.58 | 81.53 | 82.33 | 78.31 | 81.62 | 83.52 | 78.16 | 82.83 |
|  | Weed pressure | 99.59 | 97.59 | 97.98 | 97.92 | 94.83 | 100.00 | 100.00 | 99.61 | 100.00 | 100.00 | 98.75 |
|  | Performance of animals | 100.00 | 94.59 | 97.37 | 96.44 | 95.34 | 97.26 | 98.03 | 98.58 | 100.00 | 100.00 | 97.76 |
|  | Occurrence of insects |  |  |  | 98.25 | 98.83 | 98.53 | 100.00 | 100.00 | 100.00 | 100.00 | 99.37 |
|  | Occurrence of birds |  |  |  | 99.58 | 98.10 | 99.60 | 100.00 | 100.00 | 99.62 | 100.00 | 99.56 |
|  | Occurrence of mammals |  |  |  | 97.80 | 97.67 | 99.20 | 99.60 | 100.00 | 99.62 | 100.00 | 99.13 |

## Table E: Descriptive *weighted* *proportions p^w^* of the *plus*- or *changed*- categories of the monitoring characteristics (Table values are formatted as p*100)

| **Subject** | **Monitoring characteristic** | **2006** | **2007** | **2008** | **2009** | **2010** | **2011** | **2012** | **2013** | **2014** | **2015** | **Total** |
| --- | --- | --- | --- | --- | --- | --- | --- | --- | --- | --- | --- | --- |
| Agronomic practices | Crop rotation (changed) |  |  |  | 0.83 | 1.85 | 0.80 | 4.42 | 5.86 | 3.83 | 6.51 | 3.44 |
|  | Time of planting (later) | 5.96 | 3.78 | 2.69 | 1.25 | 4.06 | 1.61 | 3.61 | 5.08 | 4.21 | 6.51 | 3.88 |
|  | Tillage and planting technique (changed) | 0.00 | 0.69 | 0.00 | 0.00 | 0.37 | 0.00 | 2.01 | 1.95 | 3.07 | 3.46 | 1.15 |
|  | Insect control practices (changed) | 47.51 | 11.89 | 22.22 | 18.33 | 15.87 | 24.90 | 17.27 | 16.41 | 16.48 | 14.56 | 20.54 |
|  | Fertilizer application (changed) | 0.80 | 0.34 | 0.00 | 0.42 | 0.37 | 0.00 | 0.00 | 2.34 | 0.38 | 0.00 | 0.47 |
|  | Time of harvest (later) | 24.17 | 18.56 | 13.80 | 7.92 | 6.64 | 4.42 | 4.02 | 5.08 | 4.60 | 4.21 | 9.34 |
|  | Weed control practices (changed) | 0.41 | 0.34 | 0.34 | 0.00 | 0.37 | 0.00 | 0.00 | 0.00 | 1.92 | 0.00 | 0.34 |
|  | Fungal control practices (changed) | 0.00 | 1.05 | 0.00 | 0.42 | 0.00 | 0.00 | 0.00 | 0.00 | 0.00 | 0.00 | 0.15 |
|  | Irrigation Practices (changed) | 5.76 | 0.00 | 0.00 | 0.00 | 0.37 | 0.00 | 0.00 | 0.00 | 0.00 | 0.00 | 0.61 |
|  | Maize Borer control practice (changed) |  |  | 9.84 | 22.92 | 15.50 | 22.89 | 18.07 | 16.02 | 16.09 | 14.19 | 16.94 |
| Characteristics in the field | Germination vigor (more) | 7.94 | 6.87 | 11.45 | 14.64 | 16.24 | 5.62 | 5.62 | 7.42 | 11.88 | 13.03 | 10.07 |
|  | Time to emergence (delayed) | 5.63 | 3.79 | 2.02 | 0.84 | 0.37 | 0.00 | 0.40 | 0.39 | 0.00 | 0.00 | 1.34 |
|  | Time to male flowering (delayed) | 1.60 | 7.63 | 3.74 | 1.68 | 2.58 | 2.01 | 1.20 | 0.39 | 0.38 | 0.00 | 2.12 |
|  | Plant growth and development (delayed) | 1.62 | 4.82 | 2.70 | 2.10 | 3.69 | 0.80 | 2.01 | 0.78 | 0.00 | 0.38 | 1.89 |
|  | Incidence of stalk / root lodging (more) | 1.61 | 0.34 | 0.35 | 0.00 | 2.21 | 0.00 | 0.00 | 0.00 | 0.00 | 0.00 | 0.45 |
|  | Time to maturity (delayed) | 30.90 | 25.77 | 24.02 | 14.74 | 16.24 | 12.85 | 16.06 | 12.50 | 11.49 | 6.13 | 17.07 |
|  | Yield (higher) | 68.33 | 44.45 | 52.72 | 56.91 | 49.82 | 43.37 | 42.97 | 34.77 | 36.02 | 50.57 | 47.99 |
|  | Occurrence of volunteers (more) | 0.00 | 5.58 | 0.00 | 0.00 | 0.77 | 0.00 | 0.00 | 0.00 | 0.00 | 0.38 | 0.67 |
| Environment and wildlife | Disease susceptibility (more) | 1.98 | 1.03 | 0.67 | 0.42 | 0.37 | 0.00 | 0.00 | 0.00 | 0.00 | 0.00 | 0.45 |
|  | Pest susceptibility (more) | 1.19 | 1.37 | 0.67 | 1.30 | 0.00 | 0.00 | 0.40 | 0.39 | 0.38 | 0.00 | 0.57 |
|  | Weed pressure (more) | 0.00 | 0.34 | 0.34 | 0.00 | 0.37 | 0.00 | 0.00 | 0.39 | 0.00 | 0.00 | 0.14 |
|  | Performance of animals (changed) | 0.00 | 5.41 | 2.63 | 3.56 | 4.66 | 2.74 | 1.97 | 1.42 | 0.00 | 0.00 | 2.24 |
|  | Occurrence of insects (more) |  |  |  | 0.91 | 0.37 | 0.49 | 0.00 | 0.00 | 0.00 | 0.00 | 0.25 |
|  | Occurrence of birds (more) |  |  |  | 0.00 | 0.75 | 0.00 | 0.00 | 0.00 | 0.00 | 0.00 | 0.11 |
|  | Occurrence of mammals (more) |  |  |  | 1.32 | 1.16 | 0.40 | 0.00 | 0.00 | 0.00 | 0.00 | 0.41 |

## Table F: Descriptive *weighted* *proportions p^w^* of the *minus*- categories of the monitoring characteristics (Table values are formatted as p*100)

| **Subject** | **Monitoring characteristic** | **2006** | **2007** | **2008** | **2009** | **2010** | **2011** | **2012** | **2013** | **2014** | **2015** | **Total** |
| --- | --- | --- | --- | --- | --- | --- | --- | --- | --- | --- | --- | --- |
| Agronomic practices | Time of planting (earlier) | 1.59 | 3.44 | 2.69 | 2.92 | 1.85 | 1.20 | 0.00 | 0.00 | 0.38 | 0.38 | 1.45 |
|  | Time of harvest (earlier) | 2.39 | 3.78 | 3.37 | 2.08 | 2.21 | 0.40 | 0.00 | 0.00 | 0.38 | 0.00 | 1.46 |
| Characteristics in the field | Germination vigor (less) | 5.99 | 4.12 | 1.68 | 0.84 | 0.00 | 0.00 | 0.40 | 0.78 | 0.00 | 0.00 | 1.38 |
|  | Time to emergence (accelerated) | 6.77 | 3.10 | 6.40 | 5.47 | 4.06 | 0.80 | 0.80 | 0.00 | 0.00 | 0.38 | 2.78 |
|  | Time to male flowering (accelerated) | 0.40 | 1.73 | 4.74 | 2.08 | 3.69 | 0.00 | 0.80 | 0.78 | 0.00 | 0.00 | 1.42 |
|  | Plant growth and development (accelerated) | 6.58 | 6.90 | 9.81 | 5.85 | 7.01 | 0.80 | 1.61 | 1.17 | 0.00 | 1.15 | 4.09 |
|  | Incidence of stalk / root lodging (less) | 58.77 | 36.08 | 38.67 | 31.97 | 35.06 | 24.50 | 28.11 | 17.19 | 26.82 | 27.20 | 32.44 |
|  | Time to maturity (accelerated) | 1.99 | 4.81 | 4.33 | 2.94 | 4.06 | 0.00 | 0.00 | 0.00 | 0.00 | 0.00 | 1.81 |
|  | Yield (lower) | 2.42 | 3.87 | 4.38 | 1.68 | 1.85 | 0.00 | 2.41 | 1.95 | 1.53 | 0.00 | 2.01 |
|  | Occurrence of volunteers (less) | 27.78 | 8.65 | 15.95 | 11.86 | 9.81 | 6.97 | 3.61 | 3.91 | 1.15 | 3.83 | 9.35 |
| Environment and wildlife | Disease susceptibility (less) | 36.11 | 21.65 | 34.68 | 29.46 | 25.65 | 19.68 | 17.27 | 12.73 | 5.36 | 4.21 | 20.68 |
|  | Pest susceptibility (less) | 11.11 | 5.84 | 18.52 | 17.12 | 18.47 | 17.67 | 21.29 | 17.99 | 16.09 | 21.84 | 16.59 |
|  | Weed pressure (less) | 0.41 | 2.06 | 1.68 | 2.08 | 4.80 | 0.00 | 0.00 | 0.00 | 0.00 | 0.00 | 1.10 |
|  | Occurrence of insects (less) |  |  |  | 0.83 | 0.80 | 0.99 | 0.00 | 0.00 | 0.00 | 0.00 | 0.37 |
|  | Occurrence of birds (less) |  |  |  | 0.42 | 1.15 | 0.40 | 0.00 | 0.00 | 0.38 | 0.00 | 0.34 |
|  | Occurrence of mammals (less) |  |  |  | 0.88 | 1.17 | 0.40 | 0.40 | 0.00 | 0.38 | 0.00 | 0.46 |

## Table G: Meta-analysis: *model proportions p^mo^* of the *as usual*- categories of the monitoring characteristics (Table values are formatted as p*100)

| **Subject** | **Monitoring characteristic** | **2006** | **2007** | **2008** | **2009** | **2010** | **2011** | **2012** | **2013** | **2014** | **2015** | **Total** | **CL_l_ *** | **CL_u_ *** |
| --- | --- | --- | --- | --- | --- | --- | --- | --- | --- | --- | --- | --- | --- | --- |
| Agronomic practices | Crop rotation |  |  |  | 99.75 | 98.73 | 99.70 | 96.17 | 94.65 | 96.59 | 93.89 | 97.07 | 97.05 | 97.08 |
|  | Time of planting | 91.93 | 92.70 | 94.33 | 95.15 | 92.30 | 94.87 | 93.68 | 91.74 | 91.66 | 89.35 | 92.77 | 92.76 | 92.78 |
|  | Tillage and planting technique | 100.27 | 99.55 | 100.17 | 100.05 | 99.78 | 100.17 | 98.23 | 98.29 | 97.21 | 96.81 | 99.05 | 99.05 | 99.06 |
|  | Insect control practices | 47.24 | 82.84 | 80.20 | 87.48 | 87.18 | 77.83 | 83.96 | 85.12 | 82.79 | 84.93 | 79.96 | 79.94 | 79.97 |
|  | Fertilizer application | 99.25 | 99.73 | 100.02 | 99.66 | 99.67 | 100.01 | 100.00 | 97.63 | 99.57 | 99.95 | 99.55 | 99.54 | 99.55 |
|  | Time of harvest | 69.11 | 74.53 | 78.67 | 82.41 | 82.12 | 85.25 | 84.04 | 82.11 | 80.76 | 81.51 | 80.05 | 80.04 | 80.06 |
|  | Weed control practices | 99.37 | 99.50 | 99.38 | 99.69 | 99.36 | 99.72 | 99.60 | 99.59 | 97.63 | 99.55 | 99.34 | 99.34 | 99.34 |
|  | Fungal control practices | 100.01 | 98.83 | 100.22 | 99.91 | 100.23 | 100.19 | 100.15 | 100.12 | 100.02 | 100.02 | 99.97 | 99.97 | 99.97 |
|  | Irrigation Practices | 98.26 | 100.03 | 100.00 | 99.80 | 99.43 | 99.80 | 99.80 | 99.80 | 99.81 | 99.81 | 99.65 | 99.65 | 99.66 |
|  | Maize Borer control practice |  |  | 92.57 | 75.88 | 81.08 | 73.49 | 76.50 | 78.91 | 76.59 | 78.70 | 79.21 | 79.19 | 79.23 |
| Characteristics in the field | Germination vigor | 80.84 | 83.36 | 86.48 | 86.37 | 84.12 | 94.69 | 93.32 | 91.28 | 87.54 | 86.55 | 87.45 | 87.44 | 87.47 |
|  | Time to emergence | 84.75 | 90.72 | 90.96 | 93.28 | 94.45 | 97.63 | 96.41 | 96.75 | 96.87 | 96.48 | 93.83 | 93.82 | 93.84 |
|  | Time to male flowering | 95.07 | 88.00 | 90.63 | 94.21 | 91.07 | 94.88 | 93.77 | 94.14 | 94.47 | 94.85 | 93.11 | 93.10 | 93.12 |
|  | Plant growth and development | 86.82 | 84.03 | 85.29 | 87.84 | 83.71 | 92.02 | 88.56 | 89.40 | 90.75 | 89.21 | 87.76 | 87.75 | 87.77 |
|  | Incidence of stalk / root lodging | 36.04 | 60.53 | 62.17 | 68.20 | 61.94 | 73.87 | 70.34 | 80.19 | 70.74 | 70.23 | 65.42 | 65.40 | 65.45 |
|  | Time to maturity | 64.78 | 67.39 | 71.29 | 77.77 | 75.33 | 82.46 | 77.95 | 81.17 | 81.22 | 86.54 | 76.59 | 76.57 | 76.61 |
|  | Yield | 23.32 | 46.97 | 40.68 | 40.12 | 44.00 | 50.99 | 47.14 | 54.67 | 52.26 | 39.26 | 43.94 | 43.92 | 43.96 |
|  | Occurrence of volunteers | 61.12 | 86.89 | 84.54 | 86.08 | 87.40 | 88.94 | 91.68 | 90.82 | 93.34 | 90.25 | 86.10 | 86.08 | 86.12 |
| Environment and wildlife | Disease susceptibility |  | 67.65 | 63.16 | 67.45 | 65.18 | 69.38 | 68.27 | 71.20 | 75.02 | 76.32 | 67.53 | 67.52 | 67.55 |
|  | Pest susceptibility | 80.60 | 84.21 | 81.02 | 83.59 | 81.32 | 82.50 | 77.70 | 81.72 | 83.76 | 78.68 | 81.51 | 81.50 | 81.52 |
|  | Weed pressure | 97.74 | 95.89 | 98.40 | 98.33 | 94.23 | 98.94 | 98.55 | 97.71 | 97.89 | 97.89 | 97.56 | 97.55 | 97.56 |
|  | Performance of animals | 103.13 | 95.98 | 98.82 | 96.50 | 90.98 | 93.36 | 94.37 | 95.82 | 96.39 | 96.39 | 96.17 | 96.15 | 96.20 |
|  | Occurrence of insects |  |  |  | 97.73 | 97.93 | 97.52 | 98.50 | 98.43 | 98.27 | 98.27 | 98.09 | 98.09 | 98.10 |
|  | Occurrence of birds |  |  |  | 99.05 | 97.29 | 98.66 | 98.79 | 98.72 | 98.31 | 98.70 | 98.50 | 98.50 | 98.51 |
|  | Occurrence of mammals |  |  |  | 96.85 | 96.73 | 98.12 | 98.18 | 98.58 | 98.09 | 98.48 | 97.86 | 97.85 | 97.87 |

*****CL_l_: lower 99% confidence limit, CL_u_: upper 99% confidence limit

## Table H: Meta-analysis: *model proportions p^mo^* of the *plus*- or *changed*- categories of the monitoring characteristics (Table values are formatted as p*100)

| **Subject** | **Monitoring characteristic** | **2006** | **2007** | **2008** | **2009** | **2010** | **2011** | **2012** | **2013** | **2014** | **2015** | **Total** | **CL_l_ *** | **CL_u_ *** |
| --- | --- | --- | --- | --- | --- | --- | --- | --- | --- | --- | --- | --- | --- | --- |
| Agronomic practices | Crop rotation (changed) |  |  |  | 0.25 | 1.27 | 0.30 | 3.83 | 5.35 | 3.41 | 6.11 | 2.93 | 2.92 | 2.95 |
|  | Time of planting (later) | 6.19 | 3.71 | 3.00 | 1.86 | 5.15 | 2.96 | 5.16 | 6.88 | 6.23 | 8.54 | 4.97 | 4.96 | 4.98 |
|  | Tillage and planting technique (changed) | -0.27 | 0.45 | -0.17 | -0.05 | 0.22 | -0.17 | 1.78 | 1.71 | 2.79 | 3.19 | 0.95 | 0.94 | 0.96 |
|  | Insect control practices (changed) | 52.76 | 17.16 | 19.80 | 12.52 | 12.82 | 22.17 | 16.04 | 14.88 | 17.21 | 15.07 | 20.04 | 20.03 | 20.06 |
|  | Fertilizer application (changed) | 0.75 | 0.27 | -0.02 | 0.34 | 0.34 | -0.01 | 0.00 | 2.37 | 0.43 | 0.05 | 0.45 | 0.45 | 0.46 |
|  | Time of harvest (later) | 27.57 | 21.17 | 16.80 | 14.08 | 13.90 | 12.40 | 13.67 | 15.41 | 16.20 | 15.83 | 16.70 | 16.69 | 16.71 |
|  | Weed control practices (changed) | 0.63 | 0.51 | 0.62 | 0.31 | 0.64 | 0.28 | 0.40 | 0.41 | 2.37 | 0.45 | 0.66 | 0.66 | 0.66 |
|  | Fungal control practices (changed) | -0.01 | 1.17 | -0.22 | 0.09 | -0.23 | -0.19 | -0.15 | -0.12 | -0.02 | -0.02 | 0.03 | 0.03 | 0.03 |
|  | Irrigation Practices (changed) | 1.74 | -0.03 | 0.00 | 0.21 | 0.57 | 0.20 | 0.20 | 0.20 | 0.19 | 0.19 | 0.35 | 0.34 | 0.35 |
|  | Maize Borer control practice (changed) |  |  | 7.43 | 24.12 | 18.92 | 26.51 | 23.50 | 21.09 | 23.41 | 21.30 | 20.79 | 20.77 | 20.81 |
| Characteristics in the field | Germination vigor (more) | 13.29 | 12.89 | 11.07 | 12.36 | 15.51 | 4.94 | 5.83 | 7.49 | 11.99 | 12.99 | 10.83 | 10.82 | 10.85 |
|  | Time to emergence (delayed) | 5.87 | 3.89 | 2.76 | 1.90 | 1.21 | 0.80 | 1.36 | 1.32 | 0.92 | 0.92 | 2.09 | 2.09 | 2.10 |
|  | Time to male flowering (delayed) | 3.22 | 9.19 | 4.75 | 3.55 | 4.15 | 3.61 | 3.56 | 2.80 | 3.03 | 2.65 | 4.05 | 4.04 | 4.06 |
|  | Plant growth and development (delayed) | 2.83 | 5.88 | 3.57 | 3.75 | 5.55 | 2.76 | 4.16 | 3.04 | 2.44 | 2.83 | 3.68 | 3.67 | 3.69 |
|  | Incidence of stalk / root lodging (more) | 2.05 | 0.77 | 0.24 | -0.33 | 2.15 | 0.02 | 0.08 | 0.15 | 0.17 | 0.17 | 0.55 | 0.54 | 0.55 |
|  | Time to maturity (delayed) | 30.80 | 25.78 | 21.98 | 18.87 | 19.24 | 15.68 | 19.46 | 15.74 | 15.38 | 10.07 | 19.30 | 19.28 | 19.32 |
|  | Yield (higher) | 73.88 | 48.84 | 54.76 | 58.29 | 54.10 | 48.86 | 50.06 | 42.91 | 45.58 | 60.11 | 53.74 | 53.71 | 53.76 |
|  | Occurrence of volunteers (more) | 2.75 | 2.74 | 2.60 | 2.57 | 3.07 | 2.63 | 2.67 | 2.71 | 2.73 | 3.11 | 2.76 | 2.75 | 2.76 |
| Environment and wildlife | Disease susceptibility (more) | 2.04 | 1.09 | 0.54 | 0.04 | 0.04 | -0.31 | -0.33 | -0.31 | -0.33 | -0.33 | 0.21 | 0.21 | 0.22 |
|  | Pest susceptibility (more) | 1.20 | 1.46 | 0.31 | 0.80 | -0.40 | -0.37 | 0.03 | 0.05 | 0.10 | -0.28 | 0.29 | 0.29 | 0.29 |
|  | Weed pressure (more) | 0.43 | 0.74 | 0.53 | 0.20 | 0.57 | 0.22 | 0.35 | 0.77 | 0.40 | 0.40 | 0.46 | 0.46 | 0.46 |
|  | Performance of animals (changed) | -3.13 | 4.03 | 1.18 | 3.50 | 9.02 | 6.64 | 5.63 | 4.18 | 3.61 | 3.61 | 3.83 | 3.80 | 3.85 |
|  | Occurrence of insects (more) |  |  |  | 0.74 | 0.28 | 0.33 | -0.10 | -0.09 | -0.01 | -0.01 | 0.16 | 0.16 | 0.17 |
|  | Occurrence of birds (more) |  |  |  | 0.65 | 1.41 | 0.67 | 0.89 | 0.91 | 0.92 | 0.92 | 0.08 | -0.06 | 0.22 |
|  | Occurrence of mammals (more) |  |  |  | 1.38 | 1.53 | 0.49 | 0.05 | -0.02 | 0.01 | 0.00 | 0.39 | 0.16 | 0.61 |

***** CL_l_: lower 99% confidence limit, CL_u_: upper 99% confidence limit

## Table I: Meta-analysis: *model proportions p^mo^* of the *minus*- categories of the monitoring characteristics (Table values are formatted as p*100)

| **Subject** | **Monitoring characteristic** | **2006** | **2007** | **2008** | **2009** | **2010** | **2011** | **2012** | **2013** | **2014** | **2015** | **Total** | **CL_l_ *** | **CL_u_ *** |
| --- | --- | --- | --- | --- | --- | --- | --- | --- | --- | --- | --- | --- | --- | --- |
| Agronomic practices | Time of planting (earlier) | 1.88 | 3.58 | 2.67 | 3.00 | 2.55 | 2.17 | 1.16 | 1.38 | 2.10 | 2.10 | 2.26 | 2.25 | 2.27 |
|  | Time of harvest (earlier) | 3.33 | 4.31 | 4.53 | 3.52 | 3.98 | 2.35 | 2.30 | 2.48 | 3.04 | 2.66 | 3.25 | 3.24 | 3.26 |
| Characteristics in the field | Germination vigor (less) | 5.88 | 3.75 | 2.46 | 1.28 | 0.38 | 0.37 | 0.85 | 1.23 | 0.46 | 0.47 | 1.71 | 1.71 | 1.72 |
|  | Time to emergence (accelerated) | 9.37 | 5.39 | 6.29 | 4.83 | 4.34 | 1.57 | 2.23 | 1.93 | 2.21 | 2.60 | 4.08 | 4.07 | 4.08 |
|  | Time to male flowering (accelerated) | 1.70 | 2.81 | 4.62 | 2.24 | 4.77 | 1.51 | 2.66 | 3.06 | 2.50 | 2.50 | 2.84 | 2.83 | 2.85 |
|  | Plant growth and development (accelerated) | 10.35 | 10.09 | 11.14 | 8.41 | 10.74 | 5.21 | 7.28 | 7.57 | 6.81 | 7.96 | 8.56 | 8.55 | 8.57 |
|  | Incidence of stalk / root lodging (less) | 61.91 | 38.70 | 37.59 | 32.14 | 35.91 | 26.11 | 29.59 | 19.66 | 29.09 | 29.60 | 34.03 | 34.01 | 34.05 |
|  | Time to maturity (accelerated) | 4.42 | 6.84 | 6.73 | 3.36 | 5.43 | 1.86 | 2.59 | 3.09 | 3.39 | 3.40 | 4.11 | 4.10 | 4.12 |
|  | Yield (lower) | 2.79 | 4.20 | 4.57 | 1.59 | 1.91 | 0.15 | 2.80 | 2.42 | 2.17 | 0.63 | 2.32 | 2.32 | 2.33 |
|  | Occurrence of volunteers (less) | 36.13 | 10.38 | 12.86 | 11.35 | 9.53 | 8.43 | 5.66 | 6.47 | 3.94 | 6.64 | 11.14 | 11.12 | 11.16 |
| Environment and wildlife | Disease susceptibility (less) | 46.25 | 31.26 | 36.30 | 32.51 | 34.79 | 30.94 | 32.06 | 29.11 | 25.31 | 24.02 | 32.25 | 32.24 | 32.27 |
|  | Pest susceptibility (less) | 18.19 | 14.33 | 18.67 | 15.62 | 19.07 | 17.87 | 22.27 | 18.23 | 16.14 | 21.60 | 18.20 | 18.19 | 18.21 |
|  | Weed pressure (less) | 1.83 | 3.37 | 1.07 | 1.47 | 5.20 | 0.85 | 1.10 | 1.53 | 1.71 | 1.71 | 1.98 | 1.98 | 1.99 |
|  | Occurrence of insects (less) |  |  |  | 1.53 | 1.79 | 2.15 | 1.60 | 1.66 | 1.75 | 1.75 | 1.75 | 1.74 | 1.75 |
|  | Occurrence of birds (less) |  |  |  | 0.30 | 1.29 | 0.67 | 0.32 | 0.37 | 0.77 | 0.39 | 0.59 | 0.58 | 0.59 |
|  | Occurrence of mammals (less) |  |  |  | 0.61 | 0.90 | 0.16 | 0.12 | -0.31 | 0.13 | -0.25 | 0.19 | 0.19 | 0.20 |

***** CL_l_: lower 99% confidence limit, CL_u_: upper 99% confidence limit

## Table J: Slopes and confidence intervals of linear regression (time trends)

|  |  | **as usual** | |  | **plus** | |  | **minus** | |  |
| --- | --- | --- | --- | --- | --- | --- | --- | --- | --- | --- |
| **Subject** | **Monitoring characteristic** | **Slope** | **Lower 95% CL** | **Upper 95% CL** | **Slope** | **Lower 95% CL** | **Upper 95% CL** | **Slope** | **Lower 95% CL** | **Upper 95% CL** |
| Agronomic practices | Crop rotation | -0.2743 | -0.6956 | 0.1471 | 0.9601 | 0.3456 | 1.5745 |  |  |  |
|  | Time of planting | -0.3756 | -0.5503 | -0.2008 | 0.3998 | -0.0545 | 0.8541 | -0.1256 | -0.2919 | 0.0408 |
|  | Tillage and planting technique | 2.0823 | -0.6253 | 4.7898 | 0.3756 | 0.2008 | 0.5503 |  |  |  |
|  | Insect control practices | -0.0329 | -0.2242 | 0.1584 | -2.0823 | -4.7898 | 0.6253 |  |  |  |
|  | Fertilizer application | 1.0933 | 0.1384 | 2.0483 | 0.0329 | -0.1584 | 0.2242 |  |  |  |
|  | Time of harvest | -0.0624 | -0.2196 | 0.0948 | -0.9093 | -1.8716 | 0.0530 | -0.1840 | -0.3462 | -0.0218 |
|  | Weed control practices | 0.0528 | -0.0506 | 0.1562 | 0.0624 | -0.0948 | 0.2196 |  |  |  |
|  | Fungal control practices | 0.0713 | -0.0548 | 0.1973 | -0.0528 | -0.1562 | 0.0506 |  |  |  |
|  | Irrigation Practices | -1.1560 | -3.2492 | 0.9372 | -0.0713 | -0.1973 | 0.0548 |  |  |  |
|  | Maize Borer control practice | -0.2743 | -0.6956 | 0.1471 | 1.1560 | -0.9372 | 3.2492 |  |  |  |
| Characteristics in the field | Germination vigor | 0.8250 | -0.1580 | 1.8080 | -0.3455 | -1.2502 | 0.5592 | -0.4795 | -0.7763 | -0.1826 |
|  | Time to emergence | 1.1529 | 0.6045 | 1.7013 | -0.4522 | -0.6975 | -0.2068 | -0.7008 | -1.0461 | -0.3555 |
|  | Time to male flowering | 0.3836 | -0.1763 | 0.9434 | -0.3548 | -0.7803 | 0.0708 | -0.0288 | -0.3206 | 0.2629 |
|  | Plant growth and development | 0.6034 | 0.0371 | 1.1696 | -0.1714 | -0.4606 | 0.1179 | -0.4320 | -0.8216 | -0.0424 |
|  | Incidence of stalk / root lodging | 2.9549 | 0.8264 | 5.0833 | -0.1362 | -0.3400 | 0.0676 | -2.8187 | -4.8182 | -0.8191 |
|  | Time to maturity | 2.1196 | 1.4113 | 2.8279 | -1.7719 | -2.4003 | -1.1435 | -0.3477 | -0.7087 | 0.0133 |
|  | Yield | 1.6877 | -0.3004 | 3.6757 | -1.4303 | -3.5254 | 0.6649 | -0.2574 | -0.5665 | 0.0517 |
|  | Occurrence of volunteers | 2.1637 | 0.4287 | 3.8987 | 0.0217 | -0.0248 | 0.0682 | -2.1854 | -3.9076 | -0.4633 |
| Environment and wildlife | Disease susceptibility | 1.9392 | 0.9741 | 2.9043 | -0.2246 | -0.3369 | -0.1124 | -1.7146 | -2.6339 | -0.7953 |
|  | Pest susceptibility | -0.2028 | -0.7552 | 0.3496 | -0.1601 | -0.2789 | -0.0412 | 0.3628 | -0.2340 | 0.9597 |
|  | Weed pressure | 0.1042 | -0.2706 | 0.4790 | -0.0080 | -0.0597 | 0.0437 | -0.0962 | -0.4448 | 0.2524 |
|  | Performance of animals | -0.4651 | -1.2477 | 0.3176 | 0.4651 | -0.3176 | 1.2477 |  |  |  |
|  | Occurrence of insects | 0.1141 | -0.0331 | 0.2614 | -0.1168 | -0.2108 | -0.0227 | 0.0026 | -0.1030 | 0.1083 |
|  | Occurrence of birds | 0.0370 | -0.2667 | 0.3407 | 0.0014 | -0.1321 | 0.1349 | -0.0384 | -0.2250 | 0.1482 |
|  | Occurrence of mammals | 0.2879 | 0.0600 | 0.5159 | -0.1234 | -0.2472 | 0.0005 | -0.1646 | -0.2980 | -0.0312 |
