# Supplementary material for: Results from ten years of post-market environmental monitoring of genetically modified MON 810 maize in the European Union
Source: PLoS One. 2020 Apr 24;15(4):e0217272. doi: 10.1371/journal.pone.0217272 (PMC7182268; doi:10.1371/journal.pone.0217272)
Supplement: S3 File — (DOCX) [file pone.0217272.s003.docx]

**S3 File. Results of the statistical analysis of the farmer questionnaires (graph bars)**

Graphs: bar charts of the descriptive ***weighted proportions*** ***p^w^*** of the *as usual*-, *plus*- and *minus-* categories

Fig A. ***Weighted*** ***proportions p^w^*** of the *as usual*-, *plus*- and *minus-* categories in Agronomic practices (part 1)

Fig B. ***Weighted*** ***proportions p^w^*** of the *as usual*-, *plus*- and *minus-* categories in Agronomic practices (part 2)

Fig C. ***Weighted*** ***proportions p^w^*** of the *as usual*-, *plus*- and *minus-* categories in Characteristics in the field (part 1)

Fig D. ***Weighted*** ***proportions p^w^*** of the *as usual*-, *plus*- and *minus-* categories in Characteristics in the field (part 2)

Fig E. ***Weighted*** ***proportions p^w^*** of the *as usual*-, *plus*- and *minus-* categories in Environment and wildlife (part 1)

Fig F. ***Weighted*** ***proportions p^w^*** of the *as usual*-, *plus*- and *minus-* categories in Environment and wildlife (part 2)
