# Supplementary material for: Results from ten years of post-market environmental monitoring of genetically modified MON 810 maize in the European Union
Source: PLoS One. 2020 Apr 24;15(4):e0217272. doi: 10.1371/journal.pone.0217272 (PMC7182268; doi:10.1371/journal.pone.0217272)
Supplement: S5 File — (DOCX) [file pone.0217272.s005.docx]

**S5 File. References of the literature screening**

**FOOD AND FEED**

**Toxicity / animal feeding studies:**

2005 – 2006

Mazza, R., Soave, M., Molacchini, M., Piva, G. and Marocco, A. (2005) Assessing the transfer of genetically modified DNA from feed to animal tissues. Transgenic Research, 14, 775-784.

Rossi, F., Morlacchini, M., Fusconi, G., Pietri, A., Mazza, R. and Piva, G. (2005) Effect of Bt corn on broiler growth performance and fate of feedderived DNA in the digestive tract. Poultry Science, 84, 1022-1030.

Sanden, M., Berntssen, M.H.G., Krogdahl, A., Hemre, G.-I. and Bakke-McKellep, A.-M. (2005) An examination of the intestinal tract of Atlantic salmon, Salmo salar L., parr fed different varieties of soy and maize. Journal of Fish Diseases, 28, 317-330.

Sanden, M., Krogdahl, A., Bakke-McKellep, A.M., Buddington, R.K. and Hemre, G.I. (2006) Growth performance and organ development in Atlantic salmon, Salmon salar L. parr fed genetically modified (GM) soybean and maize. Aquaculture Nutrition, 12, 1-14.

Taylor, M.L., Hartnell, G., Nemeth, M., Karunanandaa, K. and George, B. (2005) Comparison of broiler performance when fed diets containing corn grain with insect-protected (corn rootworm and European Corn borer) and herbicide-tolerant (glyphosate) traits, control corn, or commercial reference corn. Poultry science, 84, 587-593.

2006 – 2007

Custodio, M.G., Powers, W.J., Huff-Lonergan, E., Faust, M.A. and Stein, J. (2006) Growth, pork quality and excretion characteristics of pigs fed Bt corn or non-transgenic corn. Canadian Journal of Animal Science, 461- 469.

Ferrini, A.M., Mannoni, V., Pontieri, E. and Pourshaban, M. (2007) Longer resistance of some DNA traits from Bt176 maize to gastric juice from gastrointestinal affected patients. International Journal of Immunopathology and Pharmacology, 20, 111-118.

Hammond, B.G., Dudek, R., Lemen, J.K. and Nemeth, M.A. (2006) Results of a 90-day safety assurance study with rats fed grain from corn borerprotected corn. Food and Chemical Toxicology, 44, 1092-1099.

Koch, M., Strobel, E., Tebbe, C.C., Heritage, J., Breves, G. and Huber, K. (2006) Transgenic maize in the presence of ampicillin modifies the metabolic profile and microbial population structure of bovine rumen fluid in vitro. Britisch Journal of Nutrition, 96, 820-829.

Sagstad, A., Sanden, M., Haugland, O., Hansen, A.C., Olsvik, P.A. and Hemre, G.I. (2007) Evaluation of stress- and immune-response biomarkers in Atlantic salmon, Salmo salar L., fed different levels of genetically modified maize (Bt maize), compared with its near-isogenic parental line and a commercial suprex maize. Journal of Fish Diseases, 30, 201-212.

Schwägele, F. (2007) Untersuchungen zum Ubergang von DNA-Fragmenten aus Mais auf Gewebe von Geflügel und Säugern. Fleischwirtschaft 87, 3.

Shimada, N., Miyamoto, K., Kanda, K. and Murata, H. (2006a) Bacillus thuringiensis insecticidal Cry1Ab toxin does not affect the membrane integrity of the mammalian intestinal epithelial cells: an in vitro study. In vitro Cell. Dev. Biol. - Animal, 42, 45-49.

Shimada, N., Miyamoto, K., Kanda, K. and Murata, H. (2006b) Binding of Cry1Ab toxin, a Bacillus thuringiensis insecticidal toxin, to proteins of the bovine intestinal epithelial cell: an in vitro study. Applied Entomol. Zool., 41, 295-301.

Shimada, N., Murata, H., Mikami, O., Yoshioka, M., Guruge, K.S., Yamanaka, N., Nakajima, Y. and Miyazaki, S. (2006c) Effects of feeding calves genetically modified corn Bt11: a clinico-biochemical study. J. Vet. Med. Sci., 68, 1113-1115.

Sung, H.G., Min, D.M., Kim, D.K., Li, D.Y., Kim, H.J., Upadhaya, S.D. and Ha, J.K. (2006) Influence of transgenic corn on the in vitro rumen microbial fermentation. Asian-Australasian Journal of Animal Sciences, 19, 1761-1768.

Wiedemann, S., Lutz, B., Kurtz, H., Schwarz, F.J. and Albrecht, C. (2006) In situ studies on the time-dependent degradation of recombinant corn DNA and protein in the bovine rumen. J. Anim. Sci., 84, 135-144.

2007 – 2008

Bakke-McKellep, A., Sanden, M., Danieli, A., Acierno, R., Hemre, G., Maffia, M. and Krogdahl, A. (2008) Atlantic salmon (Salmo salar L.) parr fed genetically modified soybeans and maize: Histological, digestive, metabolic, and immunological investigations, Research in Veterinary Science, 84, 395-408.

Bondzio, A., Stumpff, F., Schon, J., Martens, H. and Einspanier, R. (2008) Impact of Bacillus thuringiensis toxin Cry1Ab on rumen epithelial cells (REC) - A new in vitro model for safety assessment of recombinant food compounds, Food and Chemical Toxicology, 46, 1976-1984.

Calsamiglia, S., Hernandez, B., Hartnell, G. F. and Phipps, R. (2007) Effects of corn silage derived from a genetically modified variety containing two Transgenes on feed intake, milk production, and composition, and the absence of detectable Transgenic deoxyribonucleic acid in milk in holstein dairy cows, Journal of Dairy Science, 90, 4718-4723.

Key, S., Ma, J. and Drake, P. (2008) Genetically modified plants and human health, Journal of the Royal Society of Medicine, 101, 290-298.

Onose, J., Imai, T., Hasumura, M., Ueda, M., Ozeki, Y. and Hirose, M. (2008) Evaluation of subchronic toxicity of dietary administered Cry1Ab protein from Bacillus thuringiensis var. Kurustaki HD-1 in F344 male rats with chemically induced gastrointestinal impairment, Food and Chemical Toxicology, 46, 2184- 2189.

Paul, V., Steinke, K. and Meyer, H. H. D. (2008) Development and validation of a sensitive enzyme immunoassay for surveillance of Cry1Ab toxin in bovine blood plasma of cows fed Bt-maize (MON810), Analytica Chimica Acta, 607, 106-113.

Sagstad, A., Sanden, M., Haugland, O., Hansen, A. C., Olsvik, P. A. and Hemre, G. I. (2007) Evaluation of stress- and immune-response biomarkers in Atlantic salmon, Salmo salar L., fed different levels of genetically modified maize (Bt maize), compared with its near-isogenic parental line and a commercial suprex maize, Journal of Fish Diseases, 30, 201-212.

Schroder, M., Poulsen, M., Wilcks, A., Kroghsbo, S., Miller, A., Frenzel, T., Danier, J., Rychlik, M., Emami, K., Gatehouse, A., Shu, Q. Y., Engel, K. H., Altosaar, I. and Knudsen, I. (2007) A 90-day safety study of genetically modified rice expressing Cry1Ab protein (Bacillus thuringiensis toxin) in Wistar rats, Food and Chemical Toxicology, 45, 339-349.

Stumpff, F., Bondzio, A., Einspanier, R. and Martens, H. (2007) Effects of the Bacillus thuringiensis toxin Cry1Ab on membrane currents of isolated cells of the ruminal epithelium, Journal of Membrane Biology, 219, 37-47.

Trabalza-Marinucci, M., Brandi, G., Rondini, C., Avellini, L., Giammarini, C., Costarelli, S., Acuti, G., Orlandi, C., Filippini, G., Chiaradia, E., Malatesta, M., Crotti, S., Antonini, C., Amagliani, G., Manuali, E., Mastrogiacomo, A. R., Moscati, L., Haouet, M. N., Gaiti, A. and Magnani, M. (2008) A three-year longitudinal study on the effects of a diet containing genetically modified Bt176 maize on the health status and performance of sheep, Livestock Science, 113, 178-190.

Wiedemann, S., Gurtler, P. and Albrecht, C. (2007) Effect of feeding cows genetically modified maize on the bacterial community in the bovine rumen, Applied and Environmental Microbiology, 73, 8012-8017.

2008 – 2009

FrØystad-Saugen, M. K., Lilleeng, E., Bakke-McKellep, A. M., Vekterud, K., Valen, E. C., Hemre, Å. and Krogdahl, G. I. (2009) Distal intestinal gene expression in Atlantic salmon (*Salmo salar* L.) fed genetically modified maize, *Aquaculture Nutrition*, **15**, 104-115.

Guertler, P., Paul, V., Albrecht, C. and Meyer, H. (2008) Sensitive analytical methods for quantification of novel DNA and protein in bovine milk - first results from a long-term feeding study in dairy cows, pp 26-28.

Guertler, P., Paul, V., Albrecht, C. and Meyer, H. H. D. (2009) Sensitive and highly specific quantitative real-time PCR and ELISA for recording a potential transfer of novel DNA and Cry1Ab protein from feed into bovine milk, *Analytical and Bioanalytical Chemistry*, **393**, 1629-1638.

2009 – 2010

de Vendomois, J. S., Roullier, F., Cellier, D. and Seralini, G. E. (2009) A comparison of the effects of three gm corn on mammalian health, *International Journal of Biological Sciences*, **5**, 706-721.

Sissener, N. H., Johannessen, L. E., Hevry, E. M., Wiik-Nielsen, C. R., Berdal, K. G., Nordgreen, A. and Hemre, G. I. (2010) Zebrafish (*Danio rerio*) as a model for investigating the safety of GM feed ingredients (soya and maize); performance, stress response and uptake of dietary DNA sequences, *British Journal of Nutrition*, **103**, 3-15.

2010 – 2011

Delgado, J. and Wolt, J. (2010) Fumonisin B(1) and implications in nursery swine productivity: A quantitative exposure assessment, *Journal of Animal Science*, **88**, 3767-3777.

Steinke, K., Guertler, P., Paul, V., Wiedemann, S., Ettle, T., Albrecht, C., Meyer, H., Spiekers, H. and Schwarz, F. (2010) Effects of long-term feeding of genetically modified corn (event MON810) on the performance of lactating dairy cows, *Journal of Animal Physiology and Animal Nutrition*, **94**, e185-e193.

Swiatkiewicz, M., Hanczakowska, E., Twardowska, M., Mazur, M., Kwiatek, K., Kozaczynski, W., Swiatkiewicz, S. and Sieradzki, Z. (2011) Effect of genetically modified feeds on fattening results and transfer of transgenic DNA to swine tissues *Bulletin of the Veterinary Institute in Pulawy*, **55**, 121-125.

2011 – 2012

Rossi F, Morlacchini M, Fusconi G, Pietri A and Piva G, 2011. Effect of insertion of *Bt* gene in corn and different fumonisin content on growth performance of weaned piglets. Italian Journal of Animal Science, 10, e19.

Sissener NH, Hemre G-I, Lall SP, Sagstad A, Petersen K, Williams J, Rohloff J and Sanden M, 2011. Are apparent negative effects of feeding GM MON810 maize to Atlantic salmon, Salmo salar, caused by confounding factors? British Journal of Nutrition, 106, 42-56.

Stadnik J, Karwowska M, Dolatowski ZJ, Swiatkiewicz S and Kwiatek K, 2011. Effect of genetically modified, insect resistant corn (MON810) and glyphosate tolerant soybean meal (Roundup Ready) on physico-chemical properties of broilers' breast and thigh muscles. Bulletin of the Veterinary Institute in Pulawy, 55, 541-546.

Swiatkiewicz M, Hanczakowska E, Twardowska M, Mazur M, Kwiatek K, Kozaczynski W, Swiatkiewicz S and Sieradzki Z, 2011. Effect of genetically modified feeds on fattening results and transfer of transgenic DNA to swine tissues Bulletin of the Veterinary Institute in Pulawy, 55, 121-125.

Walsch MC, Buzoianu SG, Gardiner GE, Rea MC, Ross RP, Cassidy JP and Lawlor PG, 2012. Effects of short-term feeding of *Bt* MON810 maize on growth performance, organ morphology and function in pigs. British Journal of Nutrition, 107, 364-371.

Walsch MC, Buzoianu SG, Gardiner GE, Rea MC, Gelencser E, Janosi A, Epstein MM, Ross RP and Lawlor PG, 2011. Fate of transgenic DNA from orally administered *Bt* MON810 maize and effects on immune response and growth in pigs. PLoS ONE, 6,

2012 – 2013

Buzoianu SG, Walsh MC, Rea MC, Cassidy JP, Ross RP, Gardiner GE and Lawlor PG, 2012a. Effect of feeding genetically modified *Bt* MON810 maize to similar to 40-day-old pigs for 110 days on growth and health indicators. Animal, 6, 1609-1619.

Buzoianu SG, Walsh MC, Rea MC, O'Sullivan O, Cotter PD, Ross RP, Gardiner GE and Lawlor PG, 2012c. High-throughput sequence-based analysis of the intestinal microbiota of weanling pigs fed genetically modified MON810 maize expressing *Bacillus thuringiensis* Cry1Ab (*Bt* maize) for 31 days. Applied and Environmental Microbiology, 78, 4217-4224.

Guertler P, Brandl C, Meyer HHD and Tichopad A, 2012. Feeding genetically modified maize (MON810) to dairy cows: comparison of gene expression pattern of markers for apoptosis, inflammation and cell cycle. Journal Fur Verbraucherschutz Und Lebensmittelsicherheit- Journal of Consumer Protection and Food Safety, 7, 195-202.

Reichert M, Kozaczynski W, Karpinska TA, Bocian L, Jasik A, Kycko A, Swiatkiewicz M, Swiatkiewicz S, Furgal-Dierzuk I, Arczewska-Wlosek A, Strzetelski J and Kwiatek K, 2012. Histopathology of internal organs of farm animals fed genetically modified corn and soybean meal. Bulletin of the Veterinary Institute in Pulawy, 56, 617-622.

Sartowska K, Korwin-Kossakowska A, Sender G, Jozwik A and Prokopiuk M, 2012. The impact of genetically modified plants in the diet of Japanese quails on performance traits and the nutritional value of meat and eggs - preliminary results. Archiv Fur Geflugelkunde, 76, 140- 144.

Walsh MC, Buzoianu SG, Gardiner GE, Rea MC, O'Donovan O, Ross RP and Lawlor PG, 2013. Effects of feeding *Bt* MON810 maize to sows during first gestation and lactation on maternal and offspring health indicators. British Journal of Nutrition, 109, 873-881.

2013 – 2014

Bednarek D, Dudek K, Kwiatek K, Swiatkiewicz M, Swiatkiewicz S and Strzetelski J, 2013. Effect of a diet composed of genetically modified feed components on the selected immune parameters in pigs, cattle and poultry. Bulletin of the Veterinary Institute in Pulawy, 57, 209-217.

Buzoianu SG, Walsh MC, Cassidy JP, Ryan TP, Ross RP, Gardiner GE and Lawlor PG, 2013a. Transgenerational effects of feeding genetically modified maize to nulliparous sows and offspring on offspring growth and health. American Society of Animal Science, 91,

Buzoianu SG, Walsh MC, Rea MC, Quigley L, O'Sullivan O, Cotter PD, Ross RP, Gardiner GE and Lawlor PG, 2013b. Sequence-based analysis of the intestinal microbiota of sows and their offspring fed genetically modified maize expressing a truncated form of *Bacillus thuringiensis* Cry1Ab protein (*Bt* Maize). Applied and Environmental Microbiology, 79, 7735-7744.

Furgal-Dierzuk I, Strzetelski J, Kwiatek K, Twardowska M, Mazur M, Sieradzki Z, Kozaczynski W and Reichert M, 2014. The effect of genetically modified maize (MON 810) and soyabean meal (Roundup Ready) on rearing performance and transfer of transgenic DNA to calf tissues. Journal of Animal and Feed Sciences, 23, 13-22.

Sanden M, Ornsrud R, Sissener NH, Jorgensen S, Gu JN, Bakke AM and Hemre GI, 2013. Cross-generational feeding of *Bt* (*Bacillus thuringiensis*)-maize to zebrafish (*Danio rerio*) showed no adverse effects on the parental or offspring generations. British Journal of Nutrition, 110, 2222-2233.

Sieradzki Z, Mazur M, Kwiatek K, Swiatkiewicz S, Swiatkiewicz M, Koreleski J, Hanczakowska E, Arczewska-Wlosek A and Goldsztejn M, 2013. Assessing the possibility of genetically modified DNA transfer from GM feed to broiler, laying hen, pig and calf tissues. Polish Journal of Veterinary Sciences, 16, 435-441.

Swiatkiewicz M, Bednarek D, Markowski J, Hanczakowska E and Kwiatek K, 2013. Effect of feeding genetically modified maize and soybean meal to sows on their reproductive traits, haematological indices and offspring performance. Bulletin of the Veterinary Institute in Pulawy, 57, 413-418.

2014 – 2015

Furgal-Dieriuk I, Strzetelski J, Twardowske M, Kwiatek K and Mazur M, 2015. The effect of genetically modified feeds on productivity, milk composition, serum metabolite profiles and transfer of tDNA into milk of cows. Journal of Animal and Feed Sciences, 24, 19-30.

Gu J, Bakke AM, Valen EC, Lein I and Krogdahl A, 2014. *Bt*-maize (MON 810) and non-GM soybean meal in diets for Atlantic Salmon (*Salmo salar* L.) Juveniles - impact on survival, growth performance, development, digestive function, and transcriptional expression of intestinal immune and stress responses. Plos One, 9, 1-13.

**Molecular characterization:**

2008 – 2009

De Luis, R., Perez, M. D., Sanchez, L., Lavilla, M. and Calvo, M. (2008) Kinetic and thermodynamic parameters for heat denaturation of Cry1A(b) protein from transgenic maize (*Zea mays*), *Journal of Food Science*, **73**, C447-C451.

2009 – 2010

Aguilera, M., Querci, M., Balla, B., Prospero, A., Ermolli, M. and Van den Eede, G. (2008) A qualitative approach for the assessment of the genetic stability of the MON 810 trait in commercial seed maize varieties, *Food Analytical Methods*, **1**, 252-258.

Barros, E., Lezar, S., Anttonen, M. J., van Dijk, J. P., Rohlig, R. M., Kok, E. J. and Engel, K. H. (2010) Comparison of two GM maize varieties with a near-isogenic non-GM variety using transcriptomics, proteomics and metabolomics, *Plant Biotechnology Journal*, **8**, 436-451.

Coll, A., Nadal, A., Collado, R., Capellades, G., Kubista, M., Messeguer, J. and Pla, M. (2010) Natural variation explains most transcriptomic changes among maize plants of MON 810 and comparable non-GM varieties subjected to two N-fertilization farming practices, *Plant Molecular Biology*, **73**, 349-362.

La Paz, J. L., Vicient, C., Puigdomenech, P. and Pla, M. (2010) Characterization of polyadenylated *cryIA(b)* transcripts in maize MON 810 commercial varieties, *Analytical and Bioanalytical Chemistry*, **396**, 2125-2133.

Szekacs, A., Lauber, E., Takacs, E. and Darvas, B. (2010) Detection of Cry1Ab toxin in the leaves of *MON 810* transgenic maize, *Analytical and Bioanalytical Chemistry*, **396**, 2203-2211.

2010 – 2011

La Paz, J., Pla, M., Papazova, N., Puigdomenech, P. and Vicient, C. (2010) Stability of the MON 810 transgene in maize, *Plant Molecular Biology*, **74**, 563-571.

2011 – 2012

Neumann G, Brandes C, Joachimsthaler A and Hochegger R, 2011. Assessment of the genetic stability of GMOs with a detailed examination of MON810 using Scorpion probes. European Food Research and Technology, 233, 19-30.

2014 – 2015

La Paz JL, Pla M, Centeno E, Vicient CM and Puigdomenech P, 2014. The use of massive sequencing to detect differences between immature embryos of MON 810 and a comparable non-GM maize variety. Plos One, 9, 13.

Trtikova M, Wikmark OG, Zemp N, Widmer A and Hilbeck A, 2015. Transgene Expression and Bt Protein Content in Transgenic Bt Maize (MON 810) under Optimal and Stressful Environmental Conditions. Plos One, 10, 1-9.

**Protein expression:**

2010 - 2011

Kamath, S., Anuradha, S., Vidya, H., Mohan, K. and Dudin, Y. (2010) Quantification of *Bacillus thuringiensis* Cry1Ab protein in tissues of YieldGard (R) (MON810) corn hybrids tested at multiple field locations in India, *Crop Protection*, **29**, 921-926.

**Toxicity *in vitro* studies:**

Mesnage R, Clair E, Gress S, Then C, Szekacs A and Seralini GE, 2013. Cytotoxicity on human cells of Cry1Ab and Cry1Ac *Bt* insecticidal toxins alone or with a glyphosate-based herbicide. Journal of Applied Toxicology, 33, 695-699.

**Composition / Nutrition:**

2008 – 2009

Coll, A., Nadal, A., Palaudelmàs, M., Messeguer, J., Melé, E., Puigdomènech, P. and Pla, M. (2008) Lack of repeatable differential expression patterns between MON810 and comparable commercial varieties of maize, *Plant Molecular Biology*, **68**, 105-117.

2009 – 2010

Swiatkiewicz, S., Swiatkiewicz, M., Koreleski, J. and Kwiatek, K. (2010) Nutritional efficiency of genetically- modified insect resistant corn (MON 810) and glyphosate-tolerant soybean meal (Roundup Ready) for broilers, *Bulletin of the Veterinary Institute in Pulawy*, **54**, 43-48.

2011 – 2012

Balsamo GM, Cangahuala-Inocente GC, Bertoldo JB, Terenzi H and Arisi ACM, 2011. Proteomic analysis of four brazilian MON810 maize varieties and their four non-genetically-modified isogenic varieties. Journal of Agricultural and Food Chemistry, 59, 11553-11559.

Coll A, Nadal A, Rossignol M, Puigdomenech P and Pla M, 2011. Proteomic analysis of MON810 and comparable non-GM maize varieties grown in agricultural fields. Transgenic Research, 20, 939- 949.

Zhou J, Harrigan GG, Berman KH, Webb EG, Klusmeyer TH and Nemeth MA, 2011. Stability in the composition equivalence of grain from insect-protected maize and seed from glyphosate-tolerant soybean to conventional counterparts over multiple seasons, locations, and breeding germplasms. Journal of Agricultural and Food Chemistry, 59, 8822-8828.

Frank T, Roehlig RM, Davies HV, Barros E and Engel K-H, 2012. Metabolite profiling of maize kernels- genetic modification versus environmental influence. Journal of Agricultural and Food Chemistry, 60, 3005-3012.

Kamota A, Muchaonyerwa P and Mnkeni PNS, 2011. Effects of ensiling of *Bacillus thuringiensis* (*Bt*) maize (MON810) on degradation of the crystal 1Ab (Cry1Ab) protein and compositional quality of silage. African Journal of Biotechnology, 10, 17484-17489.

2012 – 2013

Buzoianu SG, Walsh MC, Rea MC, O'Sullivan O, Crispie F, Cotter PD, Ross RP, Gardiner GE and Lawlor PG, 2012d. The effect of feeding Bt MON 810 maize to pigs for 110 days on intestinal microbiota. PLos, 7, 1-9.

2013 – 2014

Agapito-Tenfen SZ, Guerra MP, Wikmark OG and Nodari RO, 2013. Comparative proteomic analysis of genetically modified maize grown under different agroecosystems conditions in Brazil. Proteome Science, 11,

**Protein / DNA fate in digestive tract:**

2010 – 2011

Guertler, P., Paul, V., Steinke, K., Wiedemann, S., Preissinger, W., Albrecht, C., Spiekers, H., Schwarz, F. J. and Meyer, H. (2010) Long-term feeding of genetically modified corn (MON810) - Fate of *cry1Ab* DNA and recombinant protein during the metabolism of the dairy cow, *Livestock Science*, **131**, 250-259.

Paul, V., Guertler, P., Wiedemann, S. and Meyer, H. (2010) Degradation of Cry1Ab protein from genetically modified maize (MON810) in relation to total dietary feed proteins in dairy cow digestion, *Transgenic Research*, **19**, 683-689.

Swiatkiewicz, S., Twardowska, M., Markowski, J., Mazur, M., Sieradzki, Z. and Kwiatek, K. (2010) Fate of transgenic DNA from *Bt* corn and Roundup Ready soybean meal in

broilers fed GMO feed, *Bulletin of the Veterinary Institute in Pulawy*, **54**, 237-242.

2012 – 2013

Fernandes TJR, Oliveira M and Mafra I, 2013. Tracing transgenic maize as affected by breadmaking process and raw material for the production of a traditional maize bread, broa. Food Chemistry, 138, 687-692.

**Allergenicity studies of the protein or the whole food/feed:**

2008 – 2009

Finamore, A., Roselli, M., Britti, S., Monastra, G., Ambra, R., Turrini, A. and Mengheri, E. (2008) Intestinal and peripheral immune response to MON810 maize ingestion in weaning and old mice, *Journal of Agricultural and Food Chemistry*, **56**, 11533-11539.

Guimaraes, V. D., Drumare, M. F., Ah-Leung, S., Lereclus, D., Bernard, H., Creminon, C., Wal, J. M. and Adel-Patient, K. (2008) Comparative study of the adjuvanticity of *Bacillus thuringiensis* Cry1Ab protein and cholera toxin on allergic sensitisation and elicitation to peanut, *Food and Agricultural Immunology*, **19**, 325-337.

2009 – 2010

de Luis, R., Lavilla, M., Sanchez, L., Calvo, M. and Perez, M. D. (2010) Pepsin degradation of Cry1A(b) protein purified from genetically modified maize (*Zea mays*), *Journal of Agricultural and Food Chemistry*, **58**, 2548-2553.

Guimaraes, V., Drumare, M. F., Lereclus, D., Gohar, M., Lamourette, P., Nevers, M. C., Vaisanen Tunkelrott, M. L., Bernard, H., Guillon, B., Creminon, C., Wal, J. M. and Adel-Patient, K. (2010) In vitro digestion of Cry1Ab proteins and analysis of the impact on their immunoreactivity, *Journal of Agricultural and Food Chemistry*, **58**, 3222-3231.

2010 – 2011

Adel-Patient, K., Guimaraes, V., Paris, A., Drumare, M., Ah-Leung, S., Lamourette, P., Nevers, M., Canlet, C., Molina, J., Bernard, H., Creminon, C. and Wal, J. (2011) Immunological and metabolomic impacts of administration of Cry1Ab protein and MON 810 maize in mouse, *Plos One*, **6**

Randhawa, G., Singh, M. and Grover, M. (2011) Bioinformatic analysis for allergenicity assessment of *Bacillus thuringiensis* Cry proteins expressed in insect-resistant food crops, *Food and Chemical Toxicology*, **49**, 356-362.

2012 – 2013

Buzoianu SG, Walsh MC, Rea MC, O'Donovan O, Gelencser E, Ujhelyi G, Szabo E, Nagy A, Ross RP, Gardiner GE and Lawlor PG, 2012b. Effects of feeding *Bt* maize to sows during gestation and lactation on maternal and offspring immunity and fate of transgenic material. PLoS ONE, 7, e47851-e47851.

Gu J, Krogdahl A, Sissener NH, Kortner TM, Gelencser E, Hemre G-I and Bakke AM, 2013. Effects of oral *Bt*-maize (MON810) exposure on growth and health parameters in normal and sensitised Atlantic salmon, *Salmo salar* L. British Journal of Nutrition, 109, 1408-1423.

Walsh MC, Buzoianu SG, Rea MC, O'Donovan O, Gelencser E, Ujhelyi G, Ross RP, Gardiner GE and Lawlor PG, 2012. Effects of feeding *Bt* MON810 maize to pigs for 110 days on peripheral immune response and digestive fate of the cry1Ab gene and truncated *Bt* toxin. PLoS ONE, 7.

2014 – 2015

Andreassen M, Bohn T, Wikmark OG, Van den Berg J, Lovik M, Traavik T and Nygaard UC, 2015a. Cry1Ab protein from *Bacillus thuringiensis* and MON810 cry1Ab-transgenic maize exerts no adjuvant effect after airway exposure. Scandinavian Journal of Immunology, 81, 192-200

Andreassen M, Rocca E, Bohn T, Wikmark O-G, van den Berg J, Lovik M, Traavik T and Nygaard UC, 2015b. Humoral and cellular immune responses in mice after airway administration of *Bacillus thuringiensis* Cry1Ab and MON810 cry1Ab-transgenic maize. Food and Agricultural Immunology, 26, 521-537.

Reiner D, Lee RY, Dekan G and Epstein MM, 2014. No adjuvant effect of *Bacillus thuringiensis*-maize on allergic responses in mice. Plos One, 9, 8.

**Mycotoxins:**

2005 – 2006

de la Campa, R., Hooker, D.C., Miller, J.D., Schaafsma, A.W. and Hammond, B.G. (2005) Modeling effects of environment, insect damage, and Bt genotypes on fumonisin accumulation in maize in Argentina and the Philippines. Mycopathologia, 159, 539-552.

Papst, C., Utz, H.F., Melchinger, A.E., Eder, J., Magg, T., Klein, D. and Bohn, M. (2005) Mycotoxins produced by Fusarium spp. in isogenic Bt vs. non-Bt maize hybrids under European corn borer pressure. Agronomy Journal, 97, 219-224.

Williams, W.P., Windham, G.L., Buckley, P.M. and Perkins, J.M. (2005) Southwestern corn borer damage and aflatoxin accumulation in conventional and transgenic corn hybrids. Field Crops Research, 91, 329-336.

2006 – 2007

Naef, A. and Défago, G. (2006) Population structure of plant-pathogenic Fusarium species in overwintered stalk residues from Bt-transformed and non-transformed maize crops. European Journal of Plant Pathology, 116, 129-143.

Wu, F. (2006) Mycotoxin reduction in Bt corn: potential economic, health, and regulatory impacts. Transgenic Research, 15, 277-289.

2008 – 2009

Abbas, H. K., Accinelli, C., Zablotowicz, R. M., Abel, C. A., Bruns, H. A., Dong, Y. H. and Shier, W. T. (2008) Dynamics of mycotoxin and Aspergillus flavus levels in aging Bt and non-Bt corn residues under Mississippi no-till conditions, *Journal of Agricultural and Food Chemistry*, **56**, 7578-7585.

Polisenska, I., Kubicek, J., Dohnal, V., Jirsa, O., Jezkova, A. and Spitzer, T. (2008) Maize ear rot, Fusarium mycotoxins and ergosterol content in maize hybrids, pp 381-383.

2010 – 2011

Barros, G., Magnoli, C., Reynoso, M., Ramirez, M., Farnochi, M., Torres, A., Dalcero, M., Sequeira, J., Rubinstein, C. and Chulze, S. (2009) Fungal and mycotoxin contamination in *Bt* maize and non-*Bt* maize grown in Argentina, *World Mycotoxin Journal*, **2**, 53-60.

Folcher, L., Delos, M., Marengue, E., Jarry, M., Weissenberger, A., Eychenne, N. and Regnault- Roger, C. (2010) Lower mycotoxin levels in *Bt* maize grain, *Agronomy for Sustainable Development*, **30**, 711-719.

**Others:**

**ENVIRONMENTAL SAFETY**

**Spillage and consequences thereof**

2009 – 2010

Park, K. W., Lee, B., Kim, C. G., Kim, D. Y., Park, J. Y., Ko, E. M., Jeong, S. C., Choi, K. H., Yoon, W. K. and Kim, H. M. (2010) Monitoring the occurrence of genetically modified maize at a grain receiving port and along transportation routes in the Republic of Korea, *Food Control*, **21**, 456-461.

**Agronomy**

2007 – 2008

Andersen, M. N., Sausse, C., Lacroix, B., Caul, S. and Messean, A. (2007) Agricultural studies of GM maize and the field experimental infrastructure of ECOGEN, *Pedobiologia*, **51**, 175-184.

Gomez-Barbero, M., Berbel, J. and Rodriguez-Cerezo, E. (2008) Bt corn in Spain - the performance of the EU's first GM crop, *Nature Biotechnology*, **26**, 384-386.

Krogh, P. H., Griffiths, B., Demsar, D., Bohanec, M., Debeljak, M., Andersen, M. N., Sausse, C., Birch, A. N. E., Caul, S., Holmstrup, M., Heckmann, L. H. and Cortet, J. (2007) Responses by earthworms to reduced tillage in herbicide tolerant maize and Bt maize cropping systems, *Pedobiologia*, **51**, 219-227.

2014 – 2015

Bowen KL, Flanders KL, Hagan AK and Ortiz B, 2014. Insect damage, aflatoxin content, and yield of *Bt* corn in Alabama. Journal of Economic Entomology, 107, 1818-1827.

Bowers E, Hellmich R and Munkvold G, 2014. Comparison of fumonisin contamination using HPLC and ELISA methods in *Bt* and near-isogenic maize hybrids infested with european corn borer or western bean cutworm. Journal of Agricultural and Food Chemistry, 62, 6463-6472.

Gulli M, Salvatori E, Fusaro L and Pallacani C, 2015. Comparison of drought stress response and gene expression between a GM maize variety and a near-isogenic non-GM variety. PLOs, 1-21.

**Non-Target Organisms**

2005 – 2006

Anderson, P.L., Hellmich, R.L., Prasifka, J.R. and Lewis, L.C. (2005) Effects on fitness and behavior of monarch butterfly larvae exposed to combination of Cry1Ab-expressing corn anthers and pollen. Environmental Entomology, 34, 944-952.

Babendreier, D., Kalberer, N.M., Romeis, J., Fluri, P., Mulligan, E. and Bigler, F. (2005) Influence of Bt-transgenic pollen, Bt-toxin and protease inhibitor (SBTI) ingestion on development of the hypopharyngeal glands in honeybees. Apidologie, 36, 585-594.

Daly, T. and Buntin, G.D. (2005) Effect of Bacillus thuringiensis transgenic corn for Lepidopteran control on nontarget arthropods. Environ. Entomol., 34, 1292-1301.

Eckert, J., Schuphan, I., Hothorn, L.A. and Gathmann, A. (2006) Arthropods on maize ears for detecting impacts of Bt maize on non target organisms. Environmental Entomology, 35, 554-560.

Heckmann, L., Griffiths, B., Caul, S., Thompson, J., Pusztai-Carey, M., Moar, W., Andersen, M. and Krogh, P. (2006) Consequences for Protaphorura armata (Collembola : Onychiuridae) following exposure to genetically modified Bacillus thuringiensis (Bt) maize and non-Bt maize. Environmental Pollution, 142, 212-216.

Li, W.D., Wu, K.M., Wang, X.Q., Wang, G.R. and Guo, Y.Y. (2005b) Impact of pollen grains from Bt transgenic corn on the growth and development of Chinese tussah silkworm, Antheraea pernyi (Lepidoptera : Saturniidae). Environmental Entomology, 34, 922-928.

Mattila, H., Sears, M.K. and Duan, J.J. (2005) Response of Danaus plexippus to pollen of two new Bt corn events via laboratory bioassay. Entomologia Experimentalis et Applicata, 116, 31-41.

Meissle, M., Vojtech, E. and Poppy, G.M. (2005) Effects of Bt maize-fed prey on the generalist predator Poecilus cupretis L. (Coleoptera : Carabidae). Transgenic Research, 14, 123-132.

Rodrigo-Simon, A., de Maagd, R.A., Avilla, C., Bakker, P.L., Molthoff, J., Gonzalez-Zamora, J.E. and Ferre, J. (2006) Lack of detrimental effects of Bacillus thuringiensis Cry toxins on the insect predator Chrysoperla carnea: a toxicological, histopathological, and biochemical analysis. Applied and Environmental Microbiology, 72, 1595-1603.

Tounou, A.K., Gounou, S., Borgemeister, C., Goumedzoe, Y.M.D. and Schulthess, F. (2005) Susceptibility of Eldana saccharina (Lepidoptera: Pyralidae), Busseola fusca and Sesamia calamistis (Lepidoptera: Noctuidae) to Bacillus thuringiensis Cry toxins and potential side effects of the larval parasitoid Cotesia sesamiae (Hymenoptera: Braconidae). Biocontrol Science and Technology, 15, 127-137.

Vercesi, M., Krogh, P. and Holmstrup, M. (2006) Can Bacillus thuringiensis (Bt) corn residues and Bt-corn plants affect life-history traits in the earthworm Aporrectodea aliginosa? Applied Soil Ecology, 32, 180-187.

Vojtech, E., Meissle, M. and Poppy, G.M. (2005) Effects of Bt Maize on the herbivore Spodoptera littoralis (Lepidoptera: Noctuidae) and the parasitoid Cotesia marginiventris (Hymenoptera: Braconidae). Transgenic Research, 14, 133-144.

2006 – 2007

Babendreier, D., Joller, D., Romeis, J., Bigler, F. and Widmer, F. (2007) Bacterial community structures in honeybee intestines and their reponse to two insecticidal proteins. FEMS Microbiol. Ecol., 59, 600- 610.

Babendreier, D., Kalberer, N.M., Romeis, J., Fluri, P., Mulligan, E. and Bigler, F. (2005) Influence of Bt-transgenic pollen, bt-toxin and protease inhibitor (SBTI) ingestion on development of the hypopharyngeal glands in honeybees. Apidologie, 36, 585-594.

Bakonyi, G., Szira, F., Kiss, I., Villanyi, I., Seres, A. and Szekacs, A. (2006) Preference tests with collembolas on isogenic and Bt maize. Eur. J. Soil Biol., 42, S132 - S135.

Chilcutt, C.F. (2006) Cannibalism of Helicoverpa zea (Lepidoptera: Noctuidae) from Bacillus thuringiensis (Bt) transgenic corn versus non-Bt corn. Journal of Economic Entomology, 99, 728-732.

Clark, B.W. and Coats, J.R. (2006) Subacute effects of Cry1Ab Bt corn litter on the Earthworm Eisenia fetida and the springtail Folsomia candida. Environ. Entomol., 35, 1121-1129.

Dean, J.M. and De Moraes, C.M. (2006) Effects of genetic modification on herbivore-induced volatiles from maize. J. Chem. Ecol., 32, 713-724.

Eckert, J., Schuphan, I., Hothorn, L.A. and Gathmann, A. (2006) Arthropods on maize ears for detecting impacts of Bt maize on non target organisms. Environmental Entomology, 35, 554-560.

Gathmann, A., Wirooks, L., Hothorn, L.A., D., B. and Schuphan, I. (2006) Impact of Bt maize pollen (MON 810) on lepidopteran larvae living on accompanying weeds. Molecular Ecology, 15, 2677-2685.

Harwood, J.D., Samson, A. and Obrycki, J.J. (2006) No evidence for the uptake of Cry1Ab Bt-endotoxins by the generalist predator Scarites subterraneus (Coleoptera: Carabidae) in laboratory and field experiments. Biocontrol Science and Technology, 16, 377-388.

Heckmann, L.H., Griffiths, B., Caul, S., Thomson, J., Pusztai-Carey, M., Moar, W.J., Andersen, M.N. and Krogh, P.H. (2006) Consequences for Protaphorura armata (Collembola: Onychiuridae) following exposure to genetically modified Bacillus thuringiensis (Bt) maize and non-Bt maize. Environmental Pollution, 142, 212-216.

Lang, A. and Vojtech, E. (2006) The effects of pollen consumption of transgenic Bt maize on the common swallowtail, Papilio machaon L. (Lepidoptera, papilionidae). Basic and Applied Ecology, 7, 296-306.

Ludy, C. and Lang, A. (2006) A 3-year field-scale monitoring of foliage dwelling spiders (Araneae) in transgenic Bt maize fields and adjacent field margins. Biological Control, 38.

Marvier, M., McCreedy, C., Regetz, J. and Kareiva, P. (2007) A meta-analysis of effects of Bt cotton and maize on non-target invertebrates. Science, 316, 1474-1477.

Obrist, L.B., Dutton, A., Albajes, R. and Bigler, F. (2006a) Exposure of arthropod predators to Cry1Ab toxin in Bt maize fields. Ecological Entomology, 31, 143-154.

Obrist, L.B., Dutton, A., Romeis, J. and Bigler, F. (2006b) Biological activity of Cry1Ab toxin expressed by Bt maize following ingestion by herbivorous arthropods and exposure of the predator Chrysoperla carnea. BioControl, 51, 31-48.

Obrist, L.B., Klein, H. and Dutton, A. (2006c) Assessing the effects of Bt maize on the predatory mite Neoseiulus cucumeris. Experimental and applied acarology, 38, 125-139.

Peterson, R.K.D., Meyer, S.J., Wolf, A.T., Wolt, J.D. and Davis, P.M. (2006) Genetically engineered plants, endangered species, and risk: a termporal and spatial exposure assessment for karner blue butterfly larvae and Bt maize pollen. Risk analysis, 26, 845-858.

Prasifka, P.L., Hellmich, R.L., Prasifka, J.R. and Lewis, L.C. (2007) Effects of Cry1Ab-expressing corn anthers on the movement of monarch butterfly larvae. Environ. Entomol., 36, 228-233.

Ramirez-Romero, R., Chaufaux, J. and Pham-Delègue, M.H. (2005) Effects of Cry1Ab protoxin, deltamethrin and imidacloprid on the foraging activity and the learning performances of the honeybee Apis mellifera, a comparative approach. Apidologie, 36, 601-611.

Rezac, M., Pekar, S. and Kocourek, F. (2006) Effect of Bt-maize on epigeic spiders (Araneae) and harvestmen (Opiliones). Plant Protection Science, 42, 1-8.

Rodrigo-Simon, A., de Maagd, R.A., Avilla, C., Bakker, P.L., Molthoff, J., Gonzalez-Zamora, J.E. and Ferre, J. (2006) Lack of detrimental effects of Bacillus thuringiensis Cry toxins on the insect predator Chrysoperla carnea: a toxicological, histopathological, and biochemical analysis. Applied and Environmental Microbiology, 72, 1595-1603.

Sanders, C.J., Pell, J.K., Poppy, G.M., Raybould, A., Garcia-Alonso, M. and Schuler, T.H. (2007) Host-plant mediated effects of transgenic maize on the insect parasitoid Campoletis sonorensis (Hymenoptera: Ichneumonidae). Biological Control, 40, 362-369.

Shirai, Y. (2006) Laboratory evaluation of effects of transgenic Bt corn pollen on two non-target herbivorous beetles, Epilachna vigintioctopunctata (Coccinellidae) and Galerucella vittaticollis (Chrysomelidae). Appl. Entomol. Zool., 41, 607-611.

Szekeres, D., Kadar, F. and Kiss, J. (2006a) Activity density, diversity and seasonal dynamics of ground beetles (Coleoptera: Carabidae) in Bt- (MON 810) and in isogenic maize stands. Entomologia Fennica, 17, 269-275.

Szekeres, D., Kadar, F. and Kiss, J. (2006b) Ground beetle (Coleoptera, Carabidae) assemblages in Bt-(Cry1Ab, MON 810) and isogenic maize plots in Hungary. Novenyvedelem, 47, 357-363.

Vercesi, M.L., Krogh, P.H. and Holmstrup, M. (2006) Can Bacillus thuringiensis (Bt) corn residues and Bt-corn plants affect life-history traits in the earthworm Aporrectodea caliginosa? Applied Soil Ecology, 32, 180-187.

Weber, M. and Nentwig, W. (2006) Impact of Bt corn on the diplopod Allajulus latestriatus. Pedobiologia, 50, 357-368.

2007 – 2008

Álvarez-Alfageme, F., Ferry, N., Castañera, P., Ortego, F. and Gatehouse, A. (2008) Prey mediated effects of *Bt* maize on fitness and digestive physiology of the red spider mite predator S*tethorus punctillum Weise* (Coleoptera: *Coccinellidae*), *Transgenic Research*, **epub ahead of print**

Babendreier, D., Joller, D., Romeis, J., Bigler, F. and Widmer, F. (2007) Bacterial community structures in honeybee intestines and their response to two insecticidal proteins, *FEMS Microbiology Ecology*, **59**, 600-610.

Babendreier, D., Reichhart, B., Romeis, J. and Bigler, F. (2008) Impact of insecticidal proteins expressed in transgenic plants on bumblebee microcolonies, *Entomologia Experimentalis Et Applicata*, **126**, 148-157.

Beachy, R. (2008) The burden of proof: A response to Rosi-Marshall *et al*., 2008, Letter,http://www.pnas.org/cgi/reprint/0711431105v1.

Bøhn, T., Primicerio, R., Hessen, D. and Traavik, T. (2008) Reduced Fitness of *Daphnia magna* Fed a Bt-Transgenic Maize Variety, *Archives of Environmental Contamination and Toxicology*

Bruck, D., Lopez, M., Lewis, L., Prasifka, J. and Gunnarson, R. (2007) Effects of transgenic Bacillus thuringiensis corn and permethrin on nontarget arthropods, *J. Agric. Urban Entomol.*, **23**, 111-124.

Cortet, J., Griffiths, B. S., Bohanec, M., Demsar, D., Andersen, M. N., Caul, S., Birch, A. N. E., Pernin, C., Tabone, E., de Vaufleury, A., Ke, X. and Krogh, P. H. (2007) Evaluation of effects of transgenic Bt maize on microarthropods in a European multi-site experiment, *Pedobiologia*, **51**, 207-218.

de Vaufleury, A., Kramarz, P. E., Binet, P., Cortet, J., Caul, S., Andersen, M. N., Plumey, E., Coeurdassier, M. and Krogh, P. H. (2007) Exposure and effects assessments of Bt-maize on non-target organisms (gastropods, microarthropods, mycorrhizal fungi) in microcosms, *Pedobiologia*, **51**, 185-194.

Faria, C. A., Wäckers, F. L., Pritchard, J., Barrett, D. A. and Turlings, T. C. J. (2007) High susceptability of *Bt* maize to aphids enhances the performance of parasitoids of lepidopteran pests, *Plos One*, **2**, 1-11.

Farinós, G. P., de la Poza, M., Hernandez-Crespo, P., Ortego, F. and Castanera, P. (2008) Diversity and seasonal phenology of aboveground arthropods in conventional and transgenic maize crops in Central Spain, *Biological Control*, **44**, 362-371.

Fernandes, O. A., Faria, M., Martinelli, S., Schmidt, F., Carvalho, V. F. and Moro, G. (2007) Short-term assessment of bt maize on non-target arthropods in Brazil, *Scientia Agricola*, **64**, 249-255.

Floate, K. D., Carcamo, H. A., Blackshaw, R. E., Postman, B. and Bourassa, S. (2007) Response of ground beetle (Coleoptera: *Carabidae*) field populations to four years of lepidoptera-specific *Bt* corn production, *Environmental Entomology*, **36**, 1269-1274.

Gonzalez-Zamora, J. E., Camunez, S. and Avilla, C. (2007) Effects of Bacillus thuringiensis Cry Toxins on Developmental and Reproductive Characteristics of the Predator Orius albidipennis (Hemiptera: Anthocoridae) Under Laboratory Conditions, *Environ. Entomology*, **36**, 1246-1253.

Harwood, J. D., Samson, R. A. and Obrycki, J. J. (2007) Temporal detection of Cry1Ab-endotoxins in coccinellid predators from fields of *Bacillus thuringiensis* corn, *Bulletin of Entomological Research*, **97**, 643–648.

Hoheisel, G. and Fleischer, S. (2007) Coccinellids, aphids, and pollen in diversified vegetable fields with transgenic and isoline cultivars, *Journal of Insect Science* **7**, 1-12.

Höss, S., Arndt, M., Baumgarte, S., Tebbe, C. C., Nguyen, H. T. and Jehle, J. A. (2008) Effects of transgenic corn and Cry1Ab protein on the nematode, *Caenorhabditis elegans*, *Ecotoxicology and Environmental Safety*, **70**, 334-340.

Kramarz, P. E., De Vaufleury, A. and Carey, M. (2007a) Studying the effect of exposure of the snail Helix aspersa to the purified Bt toxin, Cry1Ab, *Applied Soil Ecology*, **37**, 169-172.

Kramarz, P. E., De Vaufleury, A., Zygmunt, P. M. S. and Verdun, C. (2007b) Increased response to cadmium and *Bacillus thuringiensis* maize toxicity in the snail *Helix aspersa* infected by the nematode *Phasmarhabditis hermaphrodita*, *Environmental Toxicology and Chemistry*, **26**, 73-79.

Lawo, N. C. and Romeis, J. (2008) Assessing the utilization of a carbohydrate food source and the impact of insecticidal proteins on larvae of the green lacewing, Chrysoperla carnea, *Biological Control*, **44**, 389-398.

Leslie, T. W., Hoheisel, G. A., Biddinger, D. J., Rohr, J. R. and Fleischer, S. J. (2007) Transgenes Sustain Epigeal Insect Biodiversity in Diversified Vegetable Farm Systems, *Environmental Entomology*, **36**, 234-244.

Malone, L., Scott-Dupree, C., Todd, J. and Ramankutty, P. (2007) No sub-lethal toxicity to bumblebees, *Bombus terrestris*, exposed to *Bt*-corn pollen, captan and novaluron, *New Zealand Journal of Crop and Horticultural Science*, **35**, 435- 439.

Parrott, W. (2008) Study of *Bt* impact on caddisflies overstates its conclusions: Response to Rosi-Marshall *et al*., 2008, Letter,http://www.pnas.org/cgi/reprint/0711284105v1.

Prasifka, P. L., Hellmich, R. L., Prasfika, J. R. and Lewis, L. C. (2007) Effects of Cry1Ab-expressing corn anthers on the movement of monarch butterfly larvae, *Environ. Entomol.*, **36**, 228-233.

Ramirez-Romero, R., Bernal, J. S., Chaufaux, J. and Kaiser, L. (2007) Impact assessment of Bt-maize on a moth parasitoid, *Cotesia marginiventris* (Hymenoptera : Braconidae), via host exposure to purified Cry1Ab protein or Bt-plants, *Crop Protection*, **26**, 953-962.

Ramirez-Romero, R., Desneux, N., Chaufaux, J. and Kaiser, L. (2008) *Bt*-maize effects on biological parameters of the non-target aphid *Sitobion avenae (*Homoptera: Aphididae) and Cry1Ab toxin detection, *Pesticide Biochemistry and Physiology*, **91**, 110-115.

Ramirez-Romero, R., Desneux, N., Decourtye, A., Chaffiol, A. and Pham-Delègue, M. H. (2008) Does Cry1Ab protein affect learning performances of the honey bee *Apis mellifera* L. (Hymenoptera, Apidae)?, *Ecotoxicology and Environmental Safety*, **70**, 327-333.

Rose, R. and Dively, G. P. (2007) Effects of insecticide-treated and lepidopteran-active *Bt* transgenic sweet corn on the abundance and diversity of arthropods, *Environmental Entomology*, **36**, 1254-1268.

Rose, R., Dively, G. P. and Pettis, J. (2007) Effects of Bt corn pollen on honey bees: emphasis on protocol development, *Apidologie*, **38**, 368-377.

Rosi-Marshall, E. J., Tank, J. L., Royer, T. V., Whiles, M. R., Evans-White, M., Chambers, C., Griffiths, N. A., Pokelsek, J. and Stephen, M. L. (2007) Toxins in transgenic crop byproducts may affect headwater stream ecosystems, *Proceedings of the National Academy of Sciences*, **104**, 16204-16208.

Sabugosa-Madeira, B., Abreu, I., Ribeiro, H. and Cunha, M. (2007) *Bt* transgenic maize pollen and the silent poisoning of the hive, *Journal of Apicultural Research*, **46**, 57-58.

Sanders, C. J., Pell, J. K., Poppy, G. M., Raybould, A., Garcia-Alonso, M. and Schuler, T. H. (2007) Host-plant mediated effects of transgenic maize on the insect parasitoid *Campoletis sonorensis* (Hymenoptera: Ichneumonidae), *Biological Control*, **40**, 362-369.

Sharma, H. C., Dhillon, M. K. and Arora, R. (2008) Effects of *Bacillus thuringiensis* delta-endotoxin-fed *Helicoverpa armigera* on the survival and development of the parasitoid *Campoletis chlorideae*, *Entomologia Experimentalis et Applicata*, **126**, 1-8.

Sisterson, M. S., Carrière, Y., Dennehy, T. J. and Tabashnik, B. E. (2007) Nontarget effects of transgenic insecticidal crops: implications of source-sink population dynamics *Environmental Ecology*, **36**, 121-127.

Toschki, A., Hothorn, L. A. and Ross-Nickoll, M. (2007) Effects of cultivation of genetically modified Bt maize on epigeic arthropods (A*raneae; Carabidae*), *Environmental Entomology*, **36**, 967-981.

Wang, Z., Wu, Y., He, K. and Bai, S. (2007) Effects of transgenic *Bt* maize pollen on longevity and fecundity of *Trichogramma ostriniae* in laboratory conditions, *Bulletin of Insectology*, **60**, 49-55.

Wu, Y., Wang, Z., He, K., Bai, S. and Zhao, C. (2008) Effect of transgenic *Bt* corn (event Bt11) pollen expressing Cry1Ab toxin on longevity and fecundity of *Trichogramma ostriniae* (Hymenoptera: Trichogrammatidae) in the laboratory, *Acta Entomologica Sinica*, **51**, 227-233.

2008 – 2009

Álvarez‐Alfageme, F., Ferry, N., Castañera, P., Ortego, F. and Gatehouse, A. (2008) Prey mediated effects of *Bt* maize on fitness and digestive physiology of the red spider mite predator S*tethorus punctillum Weise* (Coleoptera: *Coccinellidae*), *Transgenic Research*, **epub ahead of print**

Aviron, S., Sanvido, O., Romeis, J., Herzog, F. and Bigler, F. (2009) Case‐specific monitoring of butterflies to determine potential effects of transgenic Bt‐maize in Switzerland, *Agriculture Ecosystems & Environment*, **131**, 137‐144.

Bøhn, T., Primicerio, R., Hessen, D. and Traavik, T. (2008) Reduced Fitness of *Daphnia magna* Fed a Bt‐Transgenic Maize Variety, *Archives of Environmental Contamination and Toxicology*

Dhillon, M. K. and Sharma, H. C. (2009) Effects of Bacillus thuringiensis ‐endotoxins Cry1Ab and Cry1Ac on the coccinellid beetle, Cheilomenes sexmaculatus (Coleoptera, Coccinellidae) under direct and indirect exposure conditions, *Biocontrol Science and Technology*, **19**, 407‐420.

Höss, S., Arndt, M., Baumgarte, S., Tebbe, C. C., Nguyen, H. T. and Jehle, J. A. (2008) Effects of transgenic corn and Cry1Ab protein on the nematode, *Caenorhabditis elegans*, *Ecotoxicology and Environmental Safety*, **70**, 334‐340.

Konrad, R., Connor, M., Ferry, N., Gatehouse, A. and Babendreier, D. (2009) Impact of transgenic oilseed rape expressing oryzacystatin‐1 (OC‐1) and of insecticidal proteins on longevity and digestive enzymes of the solitary bee *Osmia bicornis*, *Journal of Insect Physiology*, **55**, 305‐313.

Lövei, G. L., Andow, D. A. and Arpaia, S. (2009) Transgenic insecticidal crops and natural enemies: A detailed review of laboratory studies, *Environmental Entomology*, **38**, 293‐306.

Moser, S. E., Harwood, J. D. and Obrycki, J. J. (2008) Larval feeding on *Bt* hybrid and non‐*Bt* corn seedlings by *Harmonia axyridis* (Coleoptera : Coccinellidae) and *Coleomegilla maculata* (Coleoptera : Coccinellidae), *Environmental Entomology*, **37**, 525‐533.

Priestley, A. L. and Brownbridge, M. (2009) Field trials to evaluate effects of Bt‐transgenic silage corn expressing the Cry1Ab insecticidal toxin on non‐target soil arthropods in northern New England, USA, *Transgenic Research*, **18**, 425‐443.

Ramirez‐Romero, R., Desneux, N., Chaufaux, J. and Kaiser, L. (2008a) *Bt*‐maize effects on biological parameters of the non‐target aphid *Sitobion avenae (*Homoptera: Aphididae) and Cry1Ab toxin detection, *Pesticide Biochemistry and Physiology*, **91**, 110‐115.

Ramirez‐Romero, R., Desneux, N., Decourtye, A., Chaffiol, A. and Pham‐Delègue, M. H. (2008b) Does Cry1Ab protein affect learning performances of the honey bee *Apis mellifera* L. (Hymenoptera, Apidae)?, *Ecotoxicology and Environmental Safety*, **70**, 327‐333.

Schmidt, J., Braun, C., Whitehouse, L. and Hilbeck, A. (2009) Effects of activated *Bt* transgene products (Cry1Ab, Cry3Bb) on immature stages of the ladybird *Adalia bipunctata* in laboratory ecotoxicity testing, *Archives of Environmental Contamination and Toxicology*, **56**, 221‐228.

Shelton, A. M., Naranjo, S. E., Romeis, J., Hellmich, R. L., Wolt, J. D., Federici, B. A., Albajes, R., Bigler, F., Burgess, E. P. J., Dively, G. P., Gatehouse, A. M. R., Malone, L. A., Roush, R., Sears, M. and Sehnal, F. (2009) Setting the record straight: a rebuttal to an erroneous analysis on transgenic insecticidal crops and natural enemies, *Transgenic Research*, **18**, 317‐322.

Zurbrugg, C. and Nentwig, W. (2009) Ingestion and excretion of two transgenic *Bt* corn varieties by slugs, *Transgenic Research*, **18**, 215‐225.

2009 – 2010

Bohn, T., Traavik, T. and Primicerio, R. (2010) Demographic responses of *Daphnia magna* fed transgenic Bt- maize, *Ecotoxicology*, **19**, 419-430.

Cancino-Rodezno, A., Alexander, C., Villasenor, R., Pacheco, S., Porta, H., Pauchet, Y., Soberon, M., Gill, S. S. and Bravo, A. (2010) The mitogen-activated protein kinase p38 is involved in insect defense against Cry toxins from *Bacillus thuringiensis*, *Insect Biochemistry and Molecular Biology*, **40**, 58-63.

Dorhout, D. L. and Rice, M. E. (2010) Intraguild competition and enhanced survival of Western Bean Cutworm (Lepidoptera: Noctuidae) on transgenic Cry1Ab (MON 810) *Bacillus thuringiensis* corn, *Journal of Economic Entomology*, **103**, 54-62.

Erasmus, A., Van Rensburg, J. B. J. and Van Den Berg, J. (2010) Effects of Bt maize on *Agrotis segetum* (Lepidoptera: Noctuidae): a pest of maize seedlings, *Environmental Entomology*, **39**, 702-706.

Jensen, P. D., Dively, G. P., Swan, C. M. and Lamp, W. O. (2010) Exposure and non-target effects of transgenic *Bt* corn debris in streams, *Environmental Entomology*, **39**, 707-714.

Kramarz, P., de Vaufleury, A., Gimbert, F., Cortet, J., Tabone, E., Andersen, M. N. and Krogh, P. H. (2009) Effects of Bt-maize material on the life cycle of the land snail *Cantareus aspersus*, *Applied Soil Ecology*, **42**, 236-242.

Perry, J. N., Devos, Y., Arpaia, S., Bartsch, D., Gathmann, A., Hails, R. S., Kiss, J., Lheureux, K., Manachini, B., Mestdagh, S., Neemann, G., Ortego, F., Schiemann, J. and Sweet, J. B. (2010) A mathematical model of exposure of non-target Lepidoptera to *Bt-*maize pollen expressing Cry1Ab within Europe, *Proceedings of the Royal Society B-Biological Sciences*, **277**, 1417-1425.

Peterson, J. A., Obrycki, J. J. and Harwood, J. D. (2009) Quantification of Bt-endotoxin exposure pathways in carabid food webs across multiple transgenic events, *Biocontrol Science and Technology*, **19**, 613-625.

Porcar, M., Grenier, A. M., Federici, B. and Rahbe, Y. (2009) Effects of *Bacillus thuringiensis* delta-endotoxins on the Pea Aphid (*Acyrthosiphon pisum*), *Applied and Environmental Microbiology*, **75**, 4897-4900.

2010 – 2011

Álvarez-Alfageme, F., Bigler, F. and Romeis, J. (2010) Laboratory toxicity studies demonstrate no adverse effects of Cry1Ab and Cry3Bb1 to larvae of *Adalia bipunctata* (Coleoptera: Coccinellidae): the importance of study design, *Transgenic Research*, *open access online*, 1-13.

Balog, A., Kiss, J., Szekeres, D., Szenasi, A. and Marko, V. (2010) Rove beetle (Coleoptera: Staphylinidae) communities in transgenic *Bt* (MON810) and near isogenic maize, *Crop Protection*, 29, 567-571.

Garcia, M., Ortego, F., Castanera, P. and Farinos, G. P. (2010) Effects of exposure to the toxin Cry1Ab through *Bt* maize fed-prey on the performance and digestive physiology of the predatory rove beetle *Atheta coriaria*, *Biological Control*, 55, 225-233.

Knecht, S. and Nentwig, W. (2010) Effect of *Bt* maize on the reproduction and development of saprophagous Diptera over multiple generations, *Basic and Applied Ecology*, 11, 346-353.

Porcar, M., Garcia-Robles, I., Dominguez-Escriba, L. and Latorre, A. (2010) Effects of *Bacillus thuringiensis* Cry1Ab and Cry3Aa endotoxins on predatory Coleoptera tested through artificial diet-incorporation bioassays, *Bulletin of Entomological Research*, 100, 297-302.

Rauschen, S., Schaarschmidt, F. and Gathmann, A. (2010) Occurrence and field densities of Coleoptera in the maize herb layer: implications for Environmental Risk Assessment of genetically modified *Bt*-maize, *Transgenic Research*, 19, 727-744.

Lumbierres, B., Stary, P. and Pons, X. (2011) Effect of *Bt* maize on the plant-aphid-parasitoid tritrophic relationships, *Biocontrol*, 56, 133-143.

Wolt, J. and Peterson, R. (2010) Prospective formulation of environmental risk assessments: Probabilistic screening for Cry1A(b) maize risk to aquatic insects, *Ecotoxicology and Environmental Safety*, 73, 1182-1188.

Zeilinger, A. R., Andow, D. A., Zwahlen, C. and Stotzky, G. (2010) Earthworm populations in a northern US cornbelt soil are not affected by long-term cultivation of *Bt* maize expressing Cry1Ab and Cry3Bb1 proteins, *Soil Biology & Biochemistry*, 42, 1284-1292.

2011 – 2012

Hendriksma HP, Hartel S and Steffan-Dewenter I, 2011b. Testing pollen of single and stacked insect- resistant Bt-maize on in vitro reared honey bee larvae. PlosOne, 6, 1-7.

Shu Y, Ma H, Du Y, Li Z, Feng Y and Wang J, 2011. The presence of Bacillus thuringiensis (Bt) protein in earthworms Eisenia fetida has no deleterious effects on their growth and reproduction. Chemosphere, 85, 1648-1656.

Tan F, Wang J, Chen Z, Feng Y, Chi G and Rehman SU, 2011. Assessment of the arbuscular mycorrhizal fungal community in roots and rhizosphere soils of Bt corn and their non-Bt isolines. Soil Biology & Biochemistry, 43, 2473-2479.

2012 – 2013

Alcantara EP, 2012. Postcommercialization monitoring of the long-term impact of Bt corn on non- target arthropod communities in commercial farms and adjacent riparian areas in the Philippines. Environmental Entomology, 41, 1268-1276.

Bowers E, Hellmich R and Munkvold G, 2013. Vip3Aa and Cry1Ab proteins in maize reduce Fusarium ear rot and fumonisins by deterring kernel injury from multiple Lepidopteran pests. World Mycotoxin Journal, 6, 127-135.

Dutra CC, Koch RL, Burkness EC, Meissle M, Romeis J, Hutchison WD and Fernandes MG, 2012. Harmonia axyridis (Coleoptera: Coccinellidae) exhibits preference between Bt and non-Bt maize fed Spodoptera frugiperda (Lepidoptera: Noctuidae). PLoS ONE, 7,

Grabowski M and Dabrowski ZT, 2012. Evaluation of the impact of the toxic protein Cry1Ab expressed by the genetically modified cultivar MON810 on honey bee (Apis mellifera L.) behavior. Medycyna Weterynaryjna, 68, 630-633.

Hansen LS, Lovei GL and Szekacs A, 2013. Survival and development of a stored-product pest, Sitophilus zeamais (Coleoptera: Curculionidae), and its natural enemy, the parasitoid Lariophagus distinguendus (Hymenoptera: Pteromalidae), on transgenic Bt maize. Pest Management Science, 69, 602-606.

Holst N, Lang A, Lovei G and Otto M, 2013. Increased mortality is predicted of Inachis io larvae caused by Bt-maize pollen in European farmland. Ecological Modelling, 250, 126-133.

Kim YH, Hwang CE, Kim T-S and Lee SH, 2012. Risk assessment system establishment for evaluating the potential impacts of imported Bacillus thuringiensis maize on a non-target insect, Tenebrio molitor. Journal of Asia-Pacific Entomology, 15, 225-229.

Meissle M, Knecht S, Waldburger M and Romeis J, 2012. Sensitivity of the cereal leaf beetle Oulema melanopus (Coleoptera: Chrysomelidae) to Bt maize-expressed Cry3Bb1 and Cry1Ab. Arthropod-Plant Interactions, 6, 203-211.

Perez-Hedo M, Lopez C, Albajes R and Eizaguirre M, 2012. Low susceptibility of non-target Lepidopteran maize pests to the Bt protein Cry1Ab. Bulletin of Entomological Research, 102, 737-743.

van der Merwe F, Bezuidenhout C, van den Berg J and Maboeta M, 2012. Effects of Cry1Ab transgenic maize on lifecycle and biomarker responses of the earthworm, Eisenia Andrei. Sensors, 12, 17155-17167.

Wang C, Henderson G, Huang F, Gautam BK and Zhu CQ, 2012. Survival rate, food consumption, and tunneling of the formosan subterranean termite (Isoptera: Rhinotermitidae) feeding on Bt and non-Bt maize. Sociobiology, 59, 1335-1350.

2013 – 2014

Cheeke TE, Cruzan MB and Rosenstiel TN, 2013. Field evaluation of arbuscular mycorrhizal fungal colonization in Bacillus thuringiensis toxin-expressing (Bt) and non-Bt maize. Applied and Environmental Microbiology, 79, 4078-4086.

Gonzalez-Cabrera J, Garcia M, Hernandez-Crespo P, Farinos GP, Ortego F and Castanera P, 2013. Resistance to Bt maize in Mythimna unipuncta (Lepidoptera: Noctuidae) is mediated by alteration in Cry1Ab protein activation. Insect Biochemistry and Molecular Biology, 43, 635-643.

Habustova O, Dolezal P, Spitzer L, Svobodova Z, Hussein H and Sehnal F, 2014. Impact of Cry1Ab toxin expression on the non- target insects dwelling on maize plants. Journal of Applied Entomology, 138, 164-172.

Kocourek F, Saska P and Rezac M, 2013. Diversity of carabid beetles (Coleoptera: Carabidae) under three different control strategies against european corn borer in maize. Plant Protection Science, 49, 146-153.

Kuramae EE, Verbruggen E, Hillekens R, de Hollander M, Roling WFM, van der Heijden MGA and Kowalchuk GA, 2013. Tracking fungal community responses to maize plants by DNA- and RNA-based pyrosequencing. Plos One, 8,

Perez-Hedo M, Reiter D, Lopez C and Eizaguirre M, 2013. Processing of the maize Bt toxin in the gut of Mythimna unipuncta caterpillars. Entomologia Experimentalis Et Applicata, 148, 56-64.

Ondreickova H, Mihalik D, Ficek A, Hudcovicova M, Kric J and Drahovska H, 2014. Impact of genetically modified maize on the genetic diversity of rhizosphere bacteria: two- year study in Slovakia. Polish J. Ecol., 62, 1.

Twardowski J, Beres P, Hurej M and Klukowski Z, 2014. A Quantitative Assessment of the Unintended Effects of Bt-Maize (MON 810) on Rove Beetle (Col., Staphylinidae) Assemblages. Polish Journal of Environmental Studies, 23, 215-220.

Velasco GV, Kowalchukb GA, Gutierrez Ma ̃neroa FG, Ramosa B, Yergeauc E and Lucas Garcíaa JA, 2013. Increased microbial activity and nitrogen mineralization coupled to changes in microbial community structure in the rhizosphere of Bt corn. Applied Soil Ecology, 68, 46-56.

2014 – 2015

Campos RC and Hernandez MIM, 2015. Changes in the dynamics of functional groups in communities of dung beetles in Atlantic forest fragments adjacent to transgenic maize crops. Ecological Indicators, 49, 216- 227.

Čerevková A and Cagan L, 2015. Effect of transgenic insect-resistant maize to the community structure of soil nematodes in two field trials. Helminthologia, 52, 41-49.

Cotta SR, Franco Dias AC, Marriel IE, Andreote FD, Seldin L and van Elsas JD, 2014. Different effects of transgenic maize and non-transgenic maize on nitrogen-transforming *Archaea* and *Bacteria* in tropical soils. Applied and Environmental Microbiology, 80, 6437-6445.

da Silva DAF, Cotta SR, Vollu RE, Jurelevicius DD, Marques JM, Marriel IE and Seldin L, 2014. Endophytic microbial community in two transgenic maize genotypes and in their near-isogenic non-transgenic maize genotype. Bmc Microbiology, 14, 9.

Erasmus A and Van den Berg J, 2014. Effect of *Bt*-maize expressing Cry1Ab toxin on non-target *Coleoptera* and *Lepidoptera* pests of maize in South Africa. African Entomology, 22, 167-179.

Giron-Perez K, Oliveira AL, Teixeira AF, Guedes RNC and Pereira EJG, 2014. Susceptibility of Brazilian populations of *Diatraea saccharalis* to Cry1Ab and response to selection for resistance. Crop Protection, 62, 124-128.

Grabowski M, Lipska A, Zmijewska E, Kozak M and Dabrowski ZT, 2014. Transfer of the Cry1Ab toxin in tritrophic bioassays involving transgenic maize MON 810, the herbivore tetranychusurticaeKoch and the predatory ladybird beetle Adaliabipunctata L. (Coleoptera: *Coccinelidae*). Egyptian Journal of Biological Pest Control, 24, 11-16.

Habustova OS, Svobodova Z, Spitzer L, Dolezal P, Hussein HM and Sehnal F, 2015. Communities of ground- dwelling arthropods in conventional and transgenic maize: background data for the post-market environmental monitoring. Journal of Applied Entomology, 139, 31-45.

Hurej M, Mietkiewski R and Twardowski JP, 2014. The effect of Cry1AB insecticidal protein on the incidence of entomopathogenic fungi infecting aphids on *Bt* maize. Zemdirbyste-Agriculture, 101, 279-284.

Leite AN, Mendes MS, Dos Santos AC and Pereira EJ, 2014. Does Cry1 Ab maize interfere in the biology and behavioural traits of *Podisus nigrispinus*? Bulletin of Insectology, 67, 265-271.

Meissle M, Zund J, Waldburger M and Romeis J, 2014. Development of *Chrysoperla carnea* (Stephens) (Neuroptera: *Chrysopidae*) on pollen from *Bt*-transgenic and conventional maize. Scientific Reports, 4, 9.

Truter J, Van Hamburg H and Van Den Berg J, 2014. Comparative diversity of arthropods on *Bt* maize and non- *Bt* maize in two different cropping systems in South Africa. Environmental Entomology, 43, 197-208.

Zeng HL, Tan FX, Zhang YY, Feng YJ, Shu YH and Wang JW, 2014. Effects of cultivation and return of *Bacillus thuringiensis* (*Bt*) maize on the diversity of the arbuscular mycorrhizal community in soils and roots of subsequently cultivated conventional maize. Soil Biology & Biochemistry, 75, 254-263.

**Gene flow**

2008 – 2009

Kim, C. G., Yi, H., Park, S., Yeon, J. F., Kim, D. Y., Kim, D. I., Lee, K. H., Lee, T. C., Paek, I. S., Yoon, W. K., Jeong, S. C. and Kim, H. M. (2008) Monitoring the occurrence of genetically modified soybean and maize around cultivated fields and at a grain receiving port in Korea *Journal of Plant Biology*, **51**, 311‐311.

Douville, M., Gagne, F., Andre, C. and Blaise, C. (2009) Occurrence of the transgenic corn Cry1Ab gene in freshwater mussels (*Elliptio complanata*) near corn fields: Evidence of exposure by bacterial ingestion, *Ecotoxicology and Environmental Safety*, **72**, 17‐25.

Griffiths, N. A., Tank, J. L., Royer, T. V., Rosi‐Marshall, E. J., Whiles, M. R., Chambers, C. P., Frauendorf, T. C. and Evans‐White, M. A. (2009) Rapid decomposition of maize detritus in agricultural headwater streams, *Ecological Applications*, **19**, 133‐142.

**Protein/DNA fate in soil or in stream water**

2008 – 2009

Fu, Q. L., Deng, Y. L., Li, H. S., Liu, J., Hu, H. Q., Chen, S. W. and Sa, T. M. (2009) Equilibrium, kinetic and thermodynamic studies on the adsorption of the toxins of *Bacillus thuringiensis* subsp *kurstaki* by clay minerals, *Applied Surface Science*, **255**, 4551‐4557.

Frouz, J., Elhottova, D., Helingerova, M. and Kocourek, F. (2008) The effect of *Bt*‐corn on soil invertebrates, soil microbial community and decomposition rates of corn post‐harvest residues under field and laboratory conditions, *Journal of Sustainable Agriculture*, **32**, 645‐655.

Gruber, H., Paul, V., Meyer, H. H. D. and Muller, M. (2008) Validation of an enzyme immunoassay for monitoring Cry1Ab toxin in soils planted with *Bt*‐maize (MON810) in a long‐term field trial on four South German sites, pp 22‐25.

Honemann, L., Zurbrugg, C. and Nentwig, W. (2008) Effects of *Bt*‐corn decomposition on the composition of the soil meso‐ and macrofauna, *Applied Soil Ecology*, **40**, 203‐209.

Lehman, R. M., Osborne, S. L. and Rosentrater, K. A. (2008) No evidence that *bacillus thuringiensis* genes and their products influence the susceptibility of corn residue to decomposition, *Agronomy Journal*, **100**, 1687‐1693.

Margarit, E., Reggiardo, M. I. and Permingeat, H. R. (2008) *Bt* protein rhizosecreted from transgenic maize does not accumulate in soil, *Electronic Journal of Biotechnology*, **11**, 10.

Oliveira, A. P., Pampulha, M. E. and Bennett, J. P. (2008) A two‐year field study with transgenic *Bacillus thuringiensis* maize: Effects on soil microorganisms, *Science of the Total Environment*, **405**, 351‐357.

Schrader, S., Münchenberg, T., Baumgarte, S. and Tebbe, C. (2008) Earthworms of different functional groups affect the fate of the Bt‐toxin Cry1Ab from transgenic maize in soil, *European Journal of Soil Biology*, **44**, 283‐289.

Villanyi, I., Bakonyi, G. and Biro, B. (2008) Effects of genetic modification on the decomposition of corn residues measured by litter‐bag method, *Cereal Research Communications*, **36**, 475‐478.

Wang, H. Y., Ye, Q. F., Gan, J. and Wu, J. M. (2008) Adsorption of Cry1Ab protein isolated from *Bt* transgenic rice on bentone, kaolin, humic acids, and soils, *Journal of Agricultural and Food Chemistry*, **56**, 4659‐4664.

2009 – 2010

Badea, E. M., Chelu, F. and Lacatusu, A. (2010) Results regarding the levels of Cry1Ab protein in transgenic corn tissue (MON 810) and the fate of Bt protein in three soil types, *Romanian Biotechnological Letters*, **15**, 55-62.

Daudu, C. K., Muchaonyerwa, P. and Mnkeni, P. N. S. (2009) Litterbag decomposition of genetically modified maize residues and their constituent *Bacillus thuringiensis* protein (Cry1Ab) under field conditions in the central region of the Eastern Cape, South Africa, *Agriculture Ecosystems & Environment*, **134**, 153- 158.

Icoz, I., Andow, D., Zwahlen, C. and Stotzky, G. (2009) Is the Cry1Ab protein from *Bacillus thuringiensis* (Bt) taken up by plants from soils previously planted with Bt corn and by carrot from hydroponic culture?, *Bulletin of Environmental Contamination and Toxicology*, **83**, 48-58.

Raubuch, M., Behr, K., Roose, K. and Joergensen, R. G. (2010) Specific respiration rates, adenylates, and energy budgets of soil microorganisms after addition of transgenic *Bt*-maize straw, *Pedobiologia*, **53**, 191-196.

Swan, C. M., Jensen, P. D., Dively, G. P. and Lamp, W. O. (2009) Processing of transgenic crop residues in stream ecosystems, *Journal of Applied Ecology*, **46**, 1304-1313.

Zurbrugg, C., Honemann, L., Meissle, M., Romeis, J. and Nentwig, W. (2010) Decomposition dynamics and structural plant components of genetically modified *Bt* maize leaves do not differ from leaves of conventional hybrids, *Transgenic Research*, **19**, 257-267.

2010 – 2011

Emmerling, C., Strunk, H., Schobinger, U. and Schrader, S. (2011) Fragmentation of Cry1Ab protein from *Bt*-maize (MON810) through the gut of the earthworm species *Lumbricus terrestris* L., *European Journal of Soil Biology*, 47, 160-164.

Lehman, R., Osborne, S., Prischmann-Voldseth, D. and Rosentrater, K. (2010) Insect-damaged corn stalks decompose at rates similar to *Bt*-protected, non-damaged corn stalks, *Plant and Soil*, 333, 481-490.

Sander, M., Madliger, M. and Schwarzenbach, R. (2010) Adsorption of transgenic insecticidal Cry1Ab protein to SiO2. 1. forces driving adsorption, *Environmental Science & Technology*, 44, 8870- 8876.

Tank, J., Rosi-Marshall, E., Royer, T., Whiles, M., Griffiths, N., Frauendorf, T. and Treering, D. (2010) Occurrence of maize detritus and a transgenic insecticidal protein (Cry1Ab) within the stream network of an agricultural landscape, *Proceedings of the National Academy of Sciences of the United States of America*, 107, 17645-17650.

Yanni, S., Whalen, J. and Ma, B. (2011b) Field-grown *Bt* and non-*Bt* corn: yield, chemical composition, and decomposability, *Agronomy Journal*, 103, 486-493.

Yanni, S., Whalen, J., Ma, B. and Gelinas, Y. (2011c) European corn borer injury effects on lignin, carbon and nitrogen in corn tissues, *Plant and Soil*, 341, 165-177.

Yanni, S., Whalen, J., Simpson, M. and Janzen, H. (2011a) Plant lignin and nitrogen contents control carbon dioxide production and nitrogen mineralization in soils incubated with *Bt* and non-*Bt* corn residues, *Soil Biology & Biochemistry*, 43, 63-69.

2011 – 2012

Gruber H, Paul V, Guertler P, Spiekers H, Tichopad A, Meyer HHD and Mueller M, 2011. Fate of Cry1Ab protein in agricultural systems under slurry management of cows fed genetically modified maize (Zea mays L.) MON810: A quantitative assessment. Journal of Agricultural and Food Chemistry, 59, 7135-7144.

Gruber H, Paul V, Meyer HHD and Mueller M, 2012. Determination of insecticidal Cry1Ab protein in soil collected in the final growing seasons of a nine-year field trial of Bt-maize MON810. Transgenic Research, 21, 77-88.

**Toxin fate in soil, microbial communities and other**

2005 – 2006

Baumgarte, S. and Tebbe, C.C. (2005) Field studies on the environmental fate of the Cry1Ab Bt-toxin produced by transgenic maize (MON810) and its effect on bacterial communities in the maize rhizophere. Molecular Ecology, 14, 2539-2551.

Cortet, J., Andersen, M.N., Caul, S., Griffiths, B., Joffre, R., Lacroix, B., Sausse, C., Thompson, J. and Krogh, P.H. (2006) Decomposition processes under Bt (Bacillus thuringiensis) maize: results of a multi- site experiment. Soi Biology and Biochemistry, 38, 195-199.

Dubelman, S., Ayden, B.R., Bader, B.M., Brown, C.R., Jiang, C. and Vlachos, D. (2005) Cry1Ab Protein does not persist in soil after 3 years of sustained Bt Corn use. Environm. Entomol., 34, 915-921.

Flores, S., Saxena, D. and Stotzky, G. (2005) Transgenic Bt plants decompose less in soil than non-Bt plants. Soil Biology and Biochemistry, 37, 1073-1082.

Griffiths, B., Caul, S., Thompson, J., Birch, A., Scrimgeour, C., Andersen, M., Cortet, J., Messean, A., Sausse, C., Lacroix, B. and Krogh, P. (2005) A comparison of soil microbial community structure, protozoa and nematodes in field plots of conventional and genetically modified maize expressing the Bacillus thuringiensis Cry1Ab toxin. Plant and Soil, 275, 135-146.

Griffiths, B.S., Caul, S., Thompson, J., Birch, A.N., Scrimgeour, C., Cortet, J., Foggo, A., Hacket, C.A. and Krogh, P.H. (2006) Soil microbial and faunal community responses to Bt maize and insecticide in two soils. J. Environ. Qual., 35, 734-741.

Hopkins, D.W. and Gregorich, E.G. (2005) Decomposition of residues and loss of the delta-endotoxin from transgenic (Bt) corn (Zea mays L.) in soil. Canadian Journal of Soil Science, 85, 19-26.

Muchaonyerwa, P., Waladde, S., Nyamugafata, P., Mpepereki, S. and Ristori, G.G. (2004) Persistence and impact on microorganisms of Bacillus thuringiensis proteins in some Zimbabwean soils. Plant and Soil, 266, 41-46.

Rauschen, S. and Schuphan, I. (2006) Fate of the Cry1Ab protein from Bt- maize MON810 silage in biogas production facilities. Journal of Agricultural Chemistry, 54, 879-883.

2006 – 2007

Bruns, H.A. and Abel, C.A. (2007) Effects of nitrogen fertility on Bt endotoxin levels in maize. J. Entomol. Sci., 42.

Cortet, J., Andersen, M.N., Caul, S., Griffiths, B.S., Joffre, R., Lacroix, B., Sausse, C., Thompson, J. and Krogh, P.H. (2006) Decomposition processes under Bt (Bacillus thuringiensis) maize: results of a multi- site experiment. Soil Biol. and Biochem., 38, 195-199.

Douville, M., Gagne, F., Blaise, C. and André, C. (2007) Occurence and persistence of Bacillus thuringiensis (Bt) and transgenic Bt corn crylAb gene from and aquatic environment. Ecotoxicology and Environmental Safety, 66, 195-203.

Griffiths, B.S., Caul, S., Thompson, J., Birch, A.N., Scrimgeour, C., Cortet, J., Foggo, A., Hacket, C.A. and Krogh, P.H. (2006) Soil microbial and faunal community responses to Bt maize and insecticide in two soils. J. Environ. Qual., 35, 734-741.

Griffiths, B.S., Heckmann, L.H., Caul, S., Thomson, J., Scrimgeour, C. and Krogh, P.H. (2007) Varietal effects of eight paired lines of transgenic Bt maize and near-isogenic non-Bt maize on soil microbial and nematode community structure. Plant Biotechnology, 5, 60-68.

Lutz, B., Wiedemann, S. and Albrecht, C. (2005) Degradation of transgenic Cry1Ab DNA and protein in Bt-176 maize during the ensiling process. Journal of Animal Physiology and Animal Nutrition.

Mulder, C., Wouterse, M., Raubuch, M., Roelofs, W. and Rutgers, M. (2006) Can transgenic maize affect soil microbial communities? Plos Computational Biology, 2, 1165-1172.

Philippot, L., Kuffner, M., Chèneby, D., Depret, G., Laguerre, G. and Martin- Laurent, F. (2006) Genetic structure and activity of the nitrate- reducers community in the rhizosphere of different cultivars of maize. Plant and Soil, 287, 177-186.

Rauschen, S. and Schuphan, I. (2006) Fate of the Cry1Ab protein from Bt- maize MON810 silage in biogas production facilities. Journal of Agricultural Chemistry, 54, 879-883.

Villanyi, I., Füzy, A. and Biro, B. (2006) Non-target microorganisms affected in the rhizosphere of the transgenic Bt corn. Cereal Research Communication, 34, 105-108.

2007 – 2008

Douville, M., Gagne, F., Blaise, C. and Andre, C. (2007 (available online Feb 2006)) Occurrence and persistence of *Bacillus thuringiensis* (*Bt*) and transgenic *Bt* corn C*ry1Ab* gene from an aquatic environment, *Ecotoxicology and Environmental Safety*, **66**, 195-203.

Fang, M., Motavalli, P. P., Kremer, R. J. and Nelson, K. A. (2007) Assessing changes in soil microbial communities and carbon mineralization in Bt and non-Bt corn residue-amended soils, *Applied Soil Ecology*, **37**, 150-160.

Griffiths, B. S., Caul, S., Thompson, J., Birch, A. N. E., Cortet, J., Andersen, M. N. and Krogh, P. H. (2007a) Microbial and microfaunal community structure in cropping systems with genetically modified plants, *Pedobiologia*, **51**, 195-206.

Griffiths, B. S., Heckmann, L. H., Caul, S., Thomson, J., Scrimgeour, C. and Krogh, P. H. (2007b) Varietal effects of eight paired lines of transgenic Bt maize and near-isogenic non-Bt maize on soil microbial and nematode community structure, *Plant Biotechnology*, **5**, 60-68.

Head, G. (2007) Soil fate and non-target impact of Bt proteins in microbial sprays and transgenic Bt crops, *American Chemical Society*, 212-221.

Hönemann, L., Zurbrügg, C. and Nentwig, W. Effects of Bt-corn decomposition on the composition of the soil meso- and macrofauna, *Applied Soil Ecology*, **In Press, Corrected Proof**

Icoz, I., Saxena, D., Andow, D. A., Zwahlen, C. and Stotzky, G. (2008) Microbial populations and enzyme activities in soil In Situ under transgenic corn expressing Cry proteins from *Bacillus thuringiensis*, *J Environ Qual*, **37**, 647- 662.

Lehman, R. M., Osborne, S. L. and Rosentrater, K. A. (2008) No differences in decomposition rates observed between *Bacillus thuringiensis* and non-*Bacillus thuringiensis* corn residue incubated in the field, *Agron J*, **100**, 163-168.

Marchetti, E., Accinelli, C., Talame, V. and Epifani, R. (2007) Persistence of Cry toxins and cry genes from genetically modified plants in two agricultural soils, *Agron. Sustain. Dev.*, **27**, 231-236.

Muchaonyerwa, P. and Waladde, S. (2007) Persistence of the pesticidal *Bacillus thuringiensis* protein expressed in Bt maize plant materials in two soils of the Central Eastern Cape, South Africa, *South African Journal of Plant and Soil*, **24**, 26-31.

Mulder, C., Wouterse, M., Rutgers, M. and Posthuma, L. (2007) Transgenic maize containing the Cry1Ab protein ephemerally enhances soil microbial communities, *Ambio*, **36**, 359-361.

Raubuch, M., Roose, K., Warnstorff, K., Wichern, F. and Joergensen, R. G. (2007) Respiration pattern and microbial use of field-grown transgenic Bt-maize residues, *Soil biology and biochemistry*, 1-10.

Schrader, S., Münchenberg, T., Baumgarte, S. and Tebbe, C. (2008) Earthworms of different functional groups affect the fate of the Bt-toxin Cry1Ab from transgenic maize in soil, *European Journal of Soil Biology*, **44**, 283-289.

Tarkalson, D., Kachman, S., Knops, J., Thies, J. and Wortmann, C. (2008) Decomposition of Bt and non-Bt corn hybrid residues in the field, *Nutrient Cycling in Agroecosystems*, **80**, 211-222.

Yudina, T. G., Brioukhanov, A. L., Zalunin, I. A., Revina, L. P., Shestakov, A. I., Voyushina, N. E., Chestukhina, G. G. and Netrusov, A. I. (2007) Antimicrobial activity of different proteins and their fragments from *Bacillus thuringiensis* parasporal crystals against clostridia and archaea, *Anaerobe*, **13**, 6-13.

Zwahlen, C., Hilbeck, A. and Nentwig, W. (2007) Field decomposition of transgenic Bt maize residue and the impact on non-target soil invertebrates, *Plant and Soil*, **300**, 245-257.

2012 – 2013

Barriuso J, Valverde JR and Mellado RP, 2012. Effect of Cry1Ab protein on rhizobacterial communities of Bt-maize over a four-year cultivation period. PLoS ONE, 7, e35481.

Cotta SR, Franco Dias AC, Marriel IE, Gomes EA, van Elsas JD and Seldin L, 2013. Temporal dynamics of microbial communities in the rhizosphere of two genetically modified (GM) maize hybrids in tropical agrosystems. Antonie Van Leeuwenhoek International Journal of General and Molecular Microbiology, 103, 589-601.

Londono LM, Tarkalson D and Thies JE, 2013. In-field rates of decomposition and microbial communities colonizing residues vary by depth of residue placement and plant part, but not by crop genotype for residues from two Cry1Ab Bt corn hybrids and their non-transgenic isolines. Soil Biology & Biochemistry, 57, 349-355.

Lupwayi NZ and Blackshaw RE, 2013. Soil microbial properties in Bt (Bacillus thuringiensis) corn cropping systems. Applied Soil Ecology, 63, 127-133.

Sander M, Tomaszewski JE, Madliger M and Schwarzenbach RP, 2012. Adsorption of insecticidal Cry1Ab protein to humic substances. 1. Experimental approach and mechanistic aspects. Environmental Science & Technology, 46, 9923-9931.

Verbruggen E, Kuramae EE, Hillekens R, de Hollander M, Kiers ET, Roling WFM, Kowalchuk GA and van der Heijden MGA, 2012. Testing potential effects of maize expressing the Bacillus thuringiensis Cry1Ab endotoxin (Bt Maize) on mycorrhizal fungal communities via DNA- and RNA-based pyrosequencing and molecular fingerprinting. Applied and Environmental Microbiology, 78, 7384-7392.

**IRM/ Impact of management practices**

2006 – 2007

Alves, A.P., Spencer, T.A., Tabashnik, B.E. and Siegfried, B.D. (2006) Inheritance of resistance to the Cry1Ab Bacillus thuringiensis toxin in Ostrinia nubilalis (Lepidoptera: Cramidae). Environmental Entomology, 35, 554-560.

Andreadis, S.S., Alvarez-Alfageme, F., Sanchez-Ramos, I., Stodola, T.J., Andow, D.A., Milonas, P.G., Savopoulou-Soultani, M. and Castanera, P. (2007) Frequency of resistance to Bacillus thuringiensis toxin Cry1Ab in Greek and Spanish population of Sesamia nonagrioides (Lepidoptera: Noctuidae). Journal of Economic Entomology, 100, 195- 201.

Butron, A., Sandoya, G., Santiago, R., Ordas, A., Rial, A. and Malvar, R.A. (2006) Searching for new sources of pink stem borer resistance in maize (Zea mays L.). Genetic Resources and Crop Evolution, 53, 1455- 1462.

Reardon, B.J., Sumerford, D.V. and Sappington, T.W. (2006) Dispersal of newly eclosed European corn borer adults (Lepidoptera: Crambidae) from corn into small-grain aggregation plots. Journal of Economic Entomology, 99, 1641-1650.

Saeglitz, C., Bartsch, D., Eber, S., Gathmann, A., Priesnitz, K.U. and Schuphan, I. (2006) Monitoring the Cry1Ab susceptibility of European corn borer in Germany. J. Econ. Entomol., 99, 1768-1773.

Siqueira, H.A.A., Gonzalez-Cabrera, J., Ferré, J., Flannagan, R. and Siegfried, B.D. (2006) Analyses of Cry1Ab binding in resistant and susceptible strains of the European corn borer, Ostrinia nubilalis (Hübner) (Lepidoptera: Crambidae). Applied and Environmental Microbiology, 72, 5318-5324.

Stodola, T.J., Andow, D.A., Hyden, A.R., Hinton, J.L., Roark, J.J., Buschman, L.L., Porter, P. and Cronholm, G.B. (2006) Frequency of resistance to Bacillus thuringiensis toxin Cry1Ab in southern United States corn belt population of European corn borer (Lepidoptera: Crambidae). Journal of Economic Entomology, 99, 502-507.

Tyutyunov, Y.V. (2007) Spatial model of development of resistance to transgenic insecticidal crop in a pest as applied to the European corn borer. Biofizika, 52, 95-113.

2008 – 2009

Bel, Y., Siqueira, H. A. A., Siegfried, B. D., Ferre, J. and Escriche, B. (2009) Variability in the cadherin gene in an *Ostrinia nubilalis* strain selected for Cry1Ab resistance, *Insect Biochemistry and Molecular Biology*, **39**, 218‐223.

Buntin, G. D. (2008) Corn expressing Cry1Ab or Cry1F endotoxin for fall armyworm and corn earworm (lepidoptera: noctuidae) management in field corn for grain production, *Florida Entomologist*, **91**, 523‐530.

Griko, N., Zhang, X. B., Ibrahim, M., Midboe, E. G. and Bulla, L. A. (2008) Susceptibility of *Manduca sexta* to Cry1Ab toxin of *Bacillus thuringiensis* correlates directly to developmental expression of the cadherin receptor BT‐R‐1, *Comparative Biochemistry and Physiology B‐Biochemistry & Molecular Biology*, **151**, 59‐63.

Huang, F. N., Leonard, B. R., Moore, S. H., Cook, D. R., Baldwin, J., Tindall, K. V. and Lee, D. R. (2008) Allele frequency of resistance to *Bacillus thuringiensis* Cry1Ab corn in Louisiana populations of sugarcane borer (Lepidoptera : Crambidae), *Journal of Economic Entomology*, **101**, 492‐498.

Huang, F. N., Parker, R., Leonard, R., Yong, Y. L. and Liu, J. (2009) Frequency of resistance alleles to *Bacillus thuringiensis*‐corn in Texas populations of the sugarcane borer, *Diatraea saccharalis* (F.) (Lepidoptera: Crambidae), *Crop Protection*, **28**, 174‐180.

Prasifka, J. R., Hellmich, R. L., Sumerford, D. V. and Siegfried, B. D. (2009) *Bacillus thuringiensis* resistance influences European corn borer (Lepidoptera: *Crambidae*) larval behavior after exposure to Cry1Ab, *Journal of Economic Entomology*, **102**, 781‐787.

Tyutyunov, Y., Zhadanovskaya, E., Bourguet, D. and Arditi, R. (2008) Landscape refuges delay resistance of the European corn borer to *Bt*‐maize: A demogenetic dynamic model, *Theoretical Population Biology*, **74**, 138‐146.

2009 – 2010

Arenas, I., Bravo, A., Soberon, M. and Gomez, I. (2010) Role of alkaline phosphatase from *Manduca sexta* in the mechanism of action of *Bacillus thuringiensis* Cry1Ab toxin, *Journal of biological chemistry*, **285**, 12497-12503.

Crespo, A. L. B., Spencer, T. A., Alves, A. P., Hellmich, R. L., Blankenship, E. E., Lopez, M. D., Sumerford, D. V. and Lewis, L. C. (2010) *Nosema pyrausta* and Cry1Ab-incorporated diet led to decreased survival and developmental delays in European corn borer, *Entomologia Experimentalis Et Applicata*, **134**, 146-153.

Goldstein, J. A., Mason, C. E. and Pesek, J. (2010) Dispersal and movement behavior of neonate European corn borer (Lepidoptera: Crambidae) on non-Bt and transgenic Bt corn, *Journal of Economic Entomology*, **103**, 331-339.

Lopez, M. D., Sumerford, D. V. and Lewis, L. C. (2010) *Nosema pyrausta* and Cry1Ab-incorporated diet led to decreased survival and developmental delays in European corn borer, *Entomologia Experimentalis Et Applicata*, **134**, 146-153.

Magalhaes, L. C. and Siegfried, B. D. (2009) On-plant survival and inheritance of resistance to Cry1Ab toxin from *Bacillus thuringiensis* in a field-derived strain of European corn borer, *Ostrinia nubilalis*, *Pest Management Science*, **65**, 1071-1081.

Prasifka, J. R., Hellmich, R. L., Crespo, A. L. B., Siegfried, B. D. and Onstad, D. W. (2010) Video-tracking and on-plant tests show Cry1Ab resistance influences behavior and survival of neonate *Ostrinia nubilalis* following exposure to Bt maize, *Journal of Insect Behavior*, **23**, 1-11.

Xu, L., Wang, Z., Zhang, J., He, K., Ferry, N. and Gatehouse, A. M. R. (2010) Cross-resistance of Cry1Ab- selected Asian corn borer to other Cry toxins, *Journal of Applied Entomology*, **134**, 429-438.

2010 – 2011

Andow, D., Farrell, S. and Hu, Y. (2010) Planting patterns of in-field refuges observed for *Bt* maize in Minnesota, *Journal of Economic Entomology*, 103, 1394-1399.

Alcantara, E., Estrada, A., Alpuerto, V. and Head, G. (2011) Monitoring Cry1Ab susceptibility in Asian corn borer (Lepidoptera: Crambidae) on *Bt* corn in the Philippines, *Crop Protection*, 30, 554-559.

Crespo, A., Spencer, T., Tan, S. and Siegfried, B. (2010) Fitness costs of Cry1Ab resistance in a field- derived strain of *Ostrinia nubilalis* (Lepidoptera: Crambidae), *Journal of Economic Entomology*, 103, 1386-1393.

Desneux, N., Ramirez-Romero, R., Bokonon-Ganta, A. H. and Bernal, J. S. (2010) Attraction of the parasitoid *Cotesia marginiventris* to host (*Spodoptera frugiperda*) frass is affected by transgenic maize, *Ecotoxicology*, 19, 1183-1192.

Engels, H., Bourguet, D., Cagan, L., Manachini, B., Schuphan, I., Stodola, T. J., Micoud, A., Brazier, C., Mottet, C. and Andow, D. A. (2010) Evaluating resistance to *Bt* toxin Cry1Ab by F(2) screen in European populations of *Ostrinia nubilalis* (Lepidoptera: Crambidae), *Journal of Economic Entomology*, 103, 1803-1809.

Feng, Y., Wang, J. and Jin, Q. (2010) Asian corn borer (*Ostrinia furnacalis*) damage induced systemic response in chemical defence in *Bt* corn (*Zea mays* L.), *Allelopathy Journal*, 26, 101-112.

Ghimire, M., Huang, F., Leonard, R., Head, G. and Yang, Y. (2011) Susceptibility of Cry1Ab-susceptible and -resistant sugarcane borer to transgenic corn plants containing single or pyramided *Bacillus thuringiensis* genes, *Crop Protection*, 30, 74-81.

Lopez, M., Sumerford, D. and Lewis, L. (2010) Effects of infection with *Nosema pyrausta* on survival and development of offspring of laboratory selected *Bt*-resistant and *Bt*-susceptible European corn borers, *Journal of Invertebrate Pathology*, 105, 248-253.

O'Rourke, M., Sappington, T. and Fleischer, S. (2010) Managing resistance to *Bt* crops in a genetically variable insect herbivore, *Ostrinia nubilalis*, *Ecological Applications*, 20, 1228-1236.

2011 – 2012

George DM, Rind FC, Bendall MW, Taylor MA and Gatehouse AMR, 2012. Developmental studies of transgenic maize expressing Cry1Ab on the African stem borer, Busseola fusca; effects on midgut cellular structure. Pest Management Science, 68, 330-339.

Kruger M, Van Rensburg JBJ and Van den Berg J, 2011. Resistance to Bt maize in Busseola fusca (Lepidoptera: Noctuidae) from Vaalharts, South Africa. Environmental Entomology, 40, 477-483.

Pérez-Hedo M, Albajes R and Eizaguirre M, 2011. Modification of hormonal balance in larvae of the corn borer Sesamia nonagrioides (Lepidoptera: Noctuidae) due to cublethal Bacillus thuringiensis protein ingestion. Journal of Economic Entomology, 104, 853-861.

Razze JM, Mason CE and Pizzolato TD, 2011. Feeding behavior of neonate Ostrinia nubilalis (Lepidoptera: Crambidae) on Cry1Ab Bt corn: implications for resistance management. Journal of Economic Entomology, 104, 806-813.

2012 - 2013

Atsumi S, Miyamoto K, Yamamoto K, Narukawa J, Kawai S, Sezutsu H, Kobayashi I, Uchino K, Tamura T, Mita K, Kadono-Okuda K, Wada S, Kanda K, Goldsmith MR and Noda H, 2012. Single amino acid mutation in an ATP-binding cassette transporter gene causes resistance to Bt toxin Cry1Ab in the silkworm, Bombyx mori. Proceedings of the National Academy of Sciences of the United States of America, 109, E1591-E1598.

Burkness EC and Hutchison WD, 2012. Bt pollen dispersal and Bt kernel mosaics: integrity of non-Bt refugia for Lepidopteran resistance management in maize. Journal of Economic Entomology, 105, 1773-1780.

Gryspeirt A and Gregoire J-C, 2012. Effects of two varieties of Bacillus thuringiensis maize on the biology of Plodia interpunctella. Toxins, 4, 373-389.

Kruger M, Van Rensburg JBJ and Van den Berg J, 2012. Reproductive biology of Bt-resistant and susceptible field-collected larvae of the maize stem borer, Busseola fusca (Lepidoptera: Noctuidae). African Entomology, 20, 35-43.

Rios-Diez JD, Siegfried B and Saldamando-Benjumea CI, 2012. Susceptibility of Spodoptera frugiperda (Lepidoptera: Noctuidae) strains from entral colombia to Cry1Ab and Cry1Ac entotoxins of Bacillus thuringiensis. Southwestern Entomologist, 37, 281-293.

2013 – 2014

Campagne P, Kruger M, Pasquet R, Le Ru B and Van den Berg J, 2013. Dominant inheritance of field-evolved resistance to Bt corn in Busseola fusca. Plos One, 8, 1-7.

Crava C, M., Farinos GP, Bel Y, Castanera P and Escriche B, 2013. Quantitative genetic analysis of Cry1Ab tolerance in Ostrinia nubilalis Spanish populations. Journal of Invertebrate Pathology, 113, 220-227.

Kruger M, Van Rensburg JBJ and Van den Berg J, 2014. No fitness costs associated with resistance of Busseola fusca (Lepidoptera: Noctuidae) to genetically modified Bt maize. Crop Protection, 55, 1-6.

2014 – 2015

Cruz D and Eizaguirre M, 2015. Do *Sesamia nonagrioides* (Lepidoptera; *Noctuidae*) gravid females discriminate between *Bt* or multivitamin corn varieties? role of olfactory and visual cues. Journal of Insect Science, 15, 5.

Reisig DD, Akin DS, All JN, Bessin RT, Brewer MJ, Buntin DG, Catchot AL, Cook D, Flanders KL, Huang FN, Johnson DW, Leonard BR, McLeod PJ, Porter RP, Reay-Jones FPF, Tindall KV, Stewart SD, Troxclair NN, Youngman RR and Rice ME, 2015. Lepidoptera (*Crambidae, Noctuidae*, and *Pyralidae*) injury to corn containing single and pyramided *Bt* traits, and blended or block refuge, in the Southern United States. Journal of Economic Entomology, 108, 157-165.

**Ecology**

2008 – 2009

Kravchenko, A. N., Hao, X. M. and Robertson, G. P. (2009) Seven years of continuously planted Bt corn did not affect mineralizable and total soil C and total N in surface soil, *Plant and Soil*, **318**, 269‐274.

2011 – 2012

Bell JR, Burkness EC, Milne AE, Onstad DW, Abrahamson M, Hamilton KL and Hutchison WD, 2012. Putting the brakes on a cycle: bottom-up effects damp cycle amplitude. Ecology Letters, 15, 310- 318.

**Other**

2008 – 2009

Achon, M. A. and Alonso‐Duenas, N. (2009) Impact of 9 years of Bt‐maize cultivation on the distribution of maize viruses, *Transgenic Research*, **18**, 387‐397.

Engels, H., Sinha, A., Schuphan, I. and Eber, S. (2008) Small‐scale dispersal of the European corn borer and its relevance for resistance management in *Bt* maize, *Journal of Applied Entomology*, **132**, 675‐680.

Gassmann, A. J., Carriere, Y. and Tabashnik, B. E. (2009) Fitness costs of insect resistance to *Bacillus thuringiensis*, *Annual Review of Entomology*, **54**, 147‐ 163.

Hubert, J., Nesvorna, M., Zemek, R., Stara, J. and Stejskal, V. (2008) Effects of metabolic inhibitors on activity of Cry1Ab toxin to inhibit growth of *Ephestia kuehniella* larvae, *Pest Management Science*, **64**, 1063‐1068.

Rauschen, S., Eckert, J., Schaarschmidt, F., Schuphan, I. and Gathmann, A. (2008) An evaluation of methods for assessing the impacts of *Bt*‐maize MON810 cultivation and pyrethroid insecticide use on *Auchenorrhyncha* (planthoppers and leafhoppers), *Agricultural and Forest Entomology*, **10**, 331‐339.

Sanvido, O., Romeis, J. and Bigler, F. (2009) An approach for post‐market monitoring of potential environmental effects of *Bt*‐maize expressing Cry1Ab on natural enemies, *Journal of Applied Entomology*, **133**, 236‐248.

Sena, J., Hernandez‐Rodriguez, C. and Ferre, J. (2009) Interaction of *Bacillus thuringiensis* Cry1 and Vip3A Proteins with spodoptera *Frugiperda* Midgut Binding Sites, *Applied and Environmental Microbiology*, **75**, 2236‐2237.

Van Wyk, A., Van den Berg, J. and Van Rensburg, J. B. J. (2009) Comparative efficacy of *Bt* maize events MON810 and Bt11 against *Sesamia calamistis* (Lepidoptera: Noctuidae) in South Africa, *Crop Protection*, **28**, 113‐116.
