# Supplementary material for: Results from ten years of post-market environmental monitoring of genetically modified MON 810 maize in the European Union
Source: PLoS One. 2020 Apr 24;15(4):e0217272. doi: 10.1371/journal.pone.0217272 (PMC7182268; doi:10.1371/journal.pone.0217272)
Supplement: S6 File — (DOCX) [file pone.0217272.s006.docx]

**S6 File. Keywords used for the literature searches**

The keywords and keyword combinations that were used are provided below as they appear in the published annual post-market environmental monitoring reports that are also published by the European Commission on their website^[[1]](#footnote-1)^. Over the years, the keywords used for the literature searches were adjusted taking into consideration newly established knowledge and experience.

**2005-2006**

**Search terms**: MON 810 or MON810; Transgenic maize or corn; Bt maize or corn; Genetically modified maize or corn; Cry1Ab and other

**2006-2007**

**Search terms**: MON 810 or MON810; *Bt* maize or corn; insect resistant maize or corn; maize or corn expressing Cry1Ab; maize or corn containing Cry1Ab

**2007-2008**

**Search terms**: ((lepidtoptera*resistan*) or (lepidtoptera* tolerant) or (insect resist*) or (insect toleran*)) and (maize or corn); ((genetically modified or genetically transformed) and (corn or maize)); (GM maize or GM corn or transgenic maize or transgenic corn or Bt maize or Bt corn); Cry1Ab; (MON 810 or MON810 or Bt176 or Bt11))

**2008-2009**

**Search terms**: ((lepidoptera* resistan*) or (lepidoptera* tolerant) or (insect resist*) or (insect toleran*)) and (maize or corn); ((genetically modified or genetically transformed) and (corn or maize)); (GM maize or GM corn or transgenic maize or transgenic corn or Bt maize or BT corn); Cry1Ab; (MON 810 or MON810 or Bt176 or Bt11))

**2009-2010**

**Search terms**: (maize or corn or Zea-mays) and (((toleran* or resistan* or protec*) same (lepidoptera* or corn-borer* or ostrinia* or nubilalis*)) and (genetically-modified or modified-genetically or transgenic* or GM or GMO or Monsanto)); ( Cry1Ab or CryIab or Cry-1Ab or CryI-Ab or Cry1A-B or CryIA-B); (MON810 or MON-810); (Bt-Maize or Bt-corn or Yieldg* or Yield-gard or Yield-guard)

**2010-2011**

| **Set** | **Search criteria** |
| --- | --- |
| #10 | #7 NOT #9  DocType=All document types; Language=All languages; |
| #9 | #8 NOT (#4 OR #5 OR #6)  DocType=All document types; Language=All languages; |
| #8 | TS= (BT176 OR BT11 OR BT-176 OR BT-11 OR CRY1A.105 OR CRY1A105 OR CRYIA105 OR CRYIA 105 OR CRYIA.105 OR CRY2AB2 OR CRYIIAB2 OR CRY2-AB2 OR CRYII-AB2 or Cry1F or Cry1Ac OR Cry3Bb1 OR Cry11* OR Cry4* OR Roundup-ready OR ((Yieldg* OR Yield-g*) SAME (rootworm OR VT OR PLUS OR PRO OR RR OR roundup)) OR (bt SAME (cotton OR soy* OR rape OR potato OR brinjal OR rice)) OR herculex OR MON89034 OR MON89034 OR TC1507 OR 59122 OR MON88017 OR MON-88017 OR MON-863 OR MON863 OR MIR604 OR DBT418 OR 15985)  DocType=All document types; Language=All languages; |
| #7 | #6 OR #5 OR #4 OR #3  DocType=All document types; Language=All languages; |
| #6 | TS= (Bt-Maize OR Bt-corn OR Yieldg* OR Yield-gard OR Yield-guard)  DocType=All document types; Language=All languages; |
| #5 | TS=(MON810 OR MON-810)  DocType=All document types; Language=All languages; |
| #4 | TS=(Cry1Ab OR CryIab OR Cry-1Ab OR CryI-Ab OR Cry1A-B OR CryIA-B)  DocType=All document types; Language=All languages; |
| #3 | #2 AND #1  DocType=All document types; Language=All languages; |
| #2 | TS=(((TOLERAN* OR RESISTAN* OR PROTEC*) SAME (LEPIDOPTERA* OR CORN-BORER* OR Ostrinia* OR nubilalis*)) AND (Genetically-modified OR modified-genetically OR transgenic* OR GM OR GMO OR MONSANTO))  DocType=All document types; Language=All languages; |
| #1 | TS=(MAIZE OR CORN OR ZEA-MAYS)  DocType=All document types; Language=All languages; |

**2011-2012**

| **Set** | **Search criteria** |
| --- | --- |
| #10 | #7 NOT #9  DocType=All document types; Language=All languages; |
| #9 | #8 NOT (#4 OR #5 OR #6)  DocType=All document types; Language=All languages; |
| #8 | TS= (BT176 OR BT11 OR BT-176 OR BT-11 OR CRY1A.105 OR CRY1A105 OR CRYIA105 OR CRYIA 105 OR CRYIA.105 OR CRY2AB2 OR CRYIIAB2 OR CRY2-AB2 OR CRYII-AB2 or Cry1F or Cry1Ac OR Cry3Bb1 OR Cry11* OR Cry4* OR Roundup-ready OR ((Yieldg* OR Yield-g*) SAME (rootworm OR VT OR PLUS OR PRO OR RR OR roundup)) OR (bt SAME (cotton OR soy* OR rape OR potato OR brinjal OR rice)) OR herculex OR MON89034 OR MON89034 OR TC1507 OR 59122 OR MON88017 OR MON-88017 OR MON-863 OR MON863 OR MIR604 OR DBT418 OR 15985)  DocType=All document types; Language=All languages; |
| #7 | #6 OR #5 OR #4 OR #3  DocType=All document types; Language=All languages; |
| #6 | TS= (Bt-Maize OR Bt-corn OR Yieldg* OR Yield-gard OR Yield-guard)  DocType=All document types; Language=All languages; |
| #5 | TS=(MON810 OR MON-810)  DocType=All document types; Language=All languages; |
| #4 | TS=(Cry1Ab OR CryIab OR Cry-1Ab OR CryI-Ab OR Cry1A-B OR CryIA-B)  DocType=All document types; Language=All languages; |
| #3 | #2 AND #1  DocType=All document types; Language=All languages; |
| #2 | TS=(((TOLERAN* OR RESISTAN* OR PROTEC*) SAME (LEPIDOPTERA* OR CORN-BORER* OR Ostrinia* OR nubilalis*)) AND (Genetically-modified OR modified-genetically OR transgenic* OR GM OR GMO OR MONSANTO))  DocType=All document types; Language=All languages; |
| #1 | TS=(MAIZE OR CORN OR ZEA-MAYS)  DocType=All document types; Language=All languages; |

**2012-2013**

| **Set** | **Search criteria** |
| --- | --- |
| #10 | #7 NOT #9  DocType=All document types; Language=All languages; |
| #9 | #8 NOT (#4 OR #5 OR #6)  DocType=All document types; Language=All languages; |
| #8 | TS= (BT176 OR BT11 OR BT-176 OR BT-11 OR CRY1A.105 OR CRY1A105 OR CRYIA105 OR CRYIA 105 OR CRYIA.105 OR CRY2AB2 OR CRYIIAB2 OR CRY2-AB2 OR CRYII-AB2 or Cry1F or Cry1Ac OR Cry3Bb1 OR Cry11* OR Cry4* OR Roundup-ready OR ((Yieldg* OR Yield-g*) SAME (rootworm OR VT OR PLUS OR PRO OR RR OR roundup)) OR (bt SAME (cotton OR soy* OR rape OR potato OR brinjal OR rice)) OR herculex OR MON89034 OR MON89034 OR TC1507 OR 59122 OR MON88017 OR MON-88017 OR MON-863 OR MON863 OR MIR604 OR DBT418 OR 15985)  DocType=All document types; Language=All languages; |
| #7 | #6 OR #5 OR #4 OR #3  DocType=All document types; Language=All languages; |
| #6 | TS= (Bt-Maize OR Bt-corn OR Yieldg* OR Yield-gard OR Yield-guard)  DocType=All document types; Language=All languages; |
| #5 | TS=(MON810 OR MON-810)  DocType=All document types; Language=All languages; |
| #4 | TS=(Cry1Ab OR CryIab OR Cry-1Ab OR CryI-Ab OR Cry1A-B OR CryIA-B)  DocType=All document types; Language=All languages; |
| #3 | #2 AND #1  DocType=All document types; Language=All languages; |
| #2 | TS=(((TOLERAN* OR RESISTAN* OR PROTEC*) SAME (LEPIDOPTERA* OR CORN-BORER* OR Ostrinia* OR nubilalis*)) AND (Genetically-modified OR modified-genetically OR transgenic* OR GM OR GMO OR MONSANTO))  DocType=All document types; Language=All languages; |
| #1 | TS=(MAIZE OR CORN OR ZEA-MAYS)  DocType=All document types; Language=All languages; |

**2013-2014**

| **Set** | **Search criteria** |
| --- | --- |
| #7 | ((#4 OR #5 OR #6))  DocType=All document types; Language=All languages; |
| #6 | (TS=(MON810 OR "MON 810"))  DocType=All document types; Language=All languages |
| #5 | (TS=(Cry1Ab OR "Cry1 Ab" OR "Cry 1 Ab" OR "Cry 1Ab" OR CryIAb OR "CryI Ab" OR "Cry I Ab" OR "Cry IAb"))  DocType=All document types; Language=All languages; |
| #4 | ((#1 and #2) OR (#1 and #3))  DocType=All document types; Language=All languages; |
| #3 | (TS=(Yield Gard OR Yieldg* OR "Bt maize" OR "Bt corn"))  DocType=All document types; Language=All languages; |
| #2 | (TS=((TOLERAN* OR RESISTANT* OR PROTEC*) near/3 (Corn near Borer* OR CornBorer OR Lepidoptera OR Ostrinia OR Sesamia)))  DocType=All document types; Language=All languages; |
| #1 | (TS=(maize* OR corn* OR "zea mays" OR "z mays"))  DocType=All document types; Language=All languages; |

**2014-2015**

| **Set** | **Search criteria** |
| --- | --- |
| #7 | ((#4 OR #5 OR #6))  DocType=All document types; Language=All languages; |
| #6 | (TS=(MON810 OR "MON 810"))  DocType=All document types; Language=All languages; |
| #5 | (TS=(Cry1Ab OR "Cry1 Ab" OR "Cry 1 Ab" OR "Cry 1Ab" OR CryIAb OR "CryI Ab" OR "Cry I Ab" OR "Cry IAb"))  DocType=All document types; Language=All languages; |
| #4 | ((#1 and #2) OR (#1 and #3))  DocType=All document types; Language=All languages; |
| #3 | (TS=(Yield Gard OR Yieldg* OR "Bt maize" OR "Bt corn"))  DocType=All document types; Language=All languages; |
| #2 | (TS=((TOLERAN* OR RESISTANT* OR PROTEC*) near/3 (Corn near Borer* OR CornBorer OR Lepidoptera OR Ostrinia OR Sesamia)))  DocType=All document types; Language=All languages; |
| #1 | (TS=(maize* OR corn* OR "zea mays" OR "z mays"))  DocType=All document types; Language=All languages; |

1. <https://ec.europa.eu/food/plant/gmo/reports_studies_en> [↑](#footnote-ref-1)
